# Infrared Spectra of Protonated Phenanthridine (C_13_H_9_NH^+^) and Isomers of Monohydrogenated Phenanthridine (C_13_H_9_NH and 1‑, 2‑, 7‑, 9‑, and 10-HC_13_H_9_N) Isolated in Solid Para-Hydrogen

**DOI:** 10.1021/acs.jpca.5c06624

**Published:** 2025-11-15

**Authors:** Man-Lin Yang, Yi-Shan Chung, Yuan-Pern Lee

**Affiliations:** † Department of Applied Chemistry and Institute of Molecular Science, National Yang Ming Chiao Tung University, Hsinchu 300093, Taiwan; ‡ Center for Emergent Functional Matter Science, 34914 National Yang Ming Chiao Tung University, Hsinchu 300093, Taiwan

## Abstract

Laboratory infrared (IR) spectra of protonated or hydrogenated polycyclic aromatic nitrogen heterocycles offer valuable insights for identifying potential carriers of interstellar unidentified infrared (UIR) emission. We report the IR spectra of protonated phenanthridine (5-phenanthridinium cation, C_13_H_9_NH^+^), its neutral radical counterpart (5-phenanthridinyl radical, C_13_H_9_NH), and additional monohydrogenated phenanthridine, produced via the electron bombardment of a mixture of phenanthridine (C_13_H_9_N) in excess *para*-hydrogen (*p*-H_2_) during matrix deposition at 3.2 K. Complementary experiments involving ultraviolet/IR irradiation of a C_13_H_9_N/Cl_2_/*p*-H_2_ matrix yielded improved spectra of HC_13_H_9_N, facilitating spectral identification of five monohydrogenated phenanthridine (1-, 2-, 7-, 9-, and 10-HC_13_H_9_N). Additionally, spectra of 3- and 6-HC_13_H_9_N were tentatively assigned because of the limited number of firmly identified lines. Spectral groupings were based on responses to secondary photolysis at varied wavelengths. Vibrational assignments were supported on comparison of experimental results with scaled harmonic vibrational wavenumbers and IR intensities of possible candidates computed using the B3LYP/6-311++G­(d,p) method. Implications of the observed IR bands in relation to the UIR emission features are discussed.

## Graphical abstract

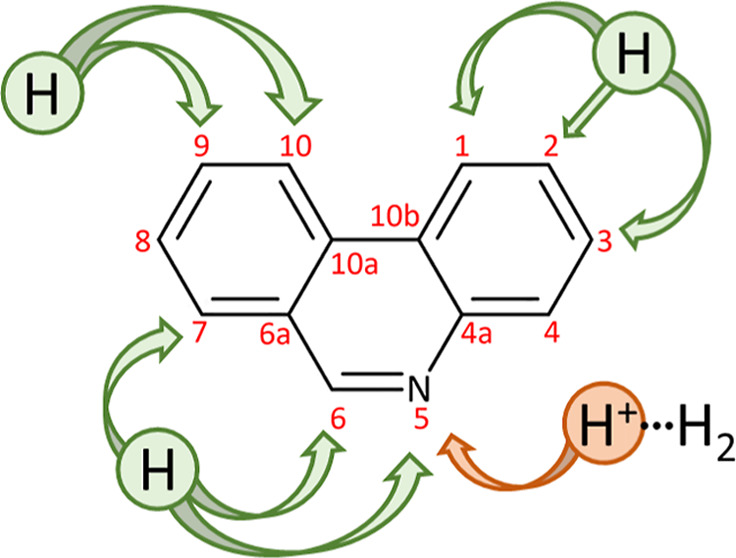

## 1 Introduction

Infrared (laboratory infrared (IR)) emission from polycyclic aromatic hydrocarbons (PAHs) upon ultraviolet (UV) excitation has long been proposed to be responsible for the interstellar unidentified infrared (UIR) emission bands, which feature prominent common emissions near 3.3, 6.2, 7.7, 8.6, and 11.2 μm.^1–6^ These bands are consistent with the CH-stretching, CC-stretching, and CH-bending vibrational modes typical of aromatic compounds. Despite extensive efforts, IR spectra of PAH have not yielded a positive match to the UIR bands. PAH cations and protonated PAH (denoted as H^+^PAH) have emerged as alternative candidates of UIR bands, supported by theoretical predictions indicating enhanced intensities of features near 6.2, 7.7, and 8.6 μm compared to their neutral counterparts, which agree better with the UIR bands.^7,8^ This enhancement has been experimentally validated for species such as protonated coronene (C_24_H_13_^+^),^9^ protonated ovalene (C_32_H_15_^+^),^10^ and nonplanar protonated corannulene (C_20_H_11_^+^).^11^ Furthermore, as the molecular size of H^+^PAH increases, spectral features shift toward the positions of the UIR bands.^12^ However, the correspondence remains unsatisfactory, suggesting that the true carriers might be even larger PAH derivatives.

The C=C stretching band positions in laboratory spectra of PAH and H^+^PAH near 6.3 μm show a slight mismatch with the corresponding feature near 6.22 μm in the class-A UIR spectra,^13^ particularly when accounting for the expected red shift between IR absorption and UV-induced emission spectra.^7,14,15^ Substitution of a CH moiety in PAH by a nitrogen atom yields polycyclic aromatic nitrogen heterocycles (PANHs), which are predicted to cause a blue shift of the C=C stretching bands of PAH, potentially improving agreement with the UIR bands near 6.2 μm.^13,15,16^ The presence of PANH in the interstellar media is plausible, as nitrogen is the fourth most abundant element after H, He, and C.^17,18^ Indeed, small PANHs such as quinoline (C_9_H_7_N), isoquinoline (*iso*-C_9_H_7_N), and related derivatives have been detected in meteorites.^19,20^ Various formation mechanisms for these species have been proposed in the literature.^21^ Because their structures bear structural similarity to nucleobases, PANHs are also considered as potentially important prebiotic compounds linked to the chemical origin of life.^22,23^

PANH exhibits a large proton affinity at the nitrogen site, typically >900 kJ mol^–1^, rendering them susceptible to protonation by H_3_^+^, which is abundant in interstellar environments. The resulting protonated PANH (designated H^+^PANH) is therefore proposed as a viable candidate for the carriers of the UIR bands. Infrared multiphoton dissociation (IRMPD) spectra of certain small PANH^+^ and H^+^PANH have been reported,^24^ but these spectra tend to be broad and have generally been acquired only within a limited spectral region, limiting direct spectral comparisons in the mid-IR range relevant to UIR features.

We have developed two complementary techniques to produce protonated and monohydrogenated PAH (and PANH). In the first approach, *para*-hydrogen (*p*-H_2_) containing a small proportion of PAH was bombarded with electrons during deposition at 3.2 K.^12,25–27^ The electron bombardment induces ionization of the H_2_ molecule, leading to the formation of H_3_^+^ and H atoms, which subsequently yield H^+^PAH and their neutral counterparts, the monohydrogenated PAH (HPAH). Alternatively, we established a photochemical method for efficient production of HPAH. This involves photolysis of a Cl_2_-doped PAH/*p*-H_2_ matrix at 365 nm to generate Cl atoms, followed by IR irradiation to drive the reaction Cl + H_2_ (*v* = 1) → HCl + H, thereby producing H atoms.^12,25–28^ In quantum-solid *p*-H_2_, these H atoms exhibit high mobility via quantum tunneling and diffuse readily throughout the matrix, inducing efficient hydrogen reactions. Our techniques enable the generation of protonated and monohydrogenated species with minimal fragmentation. Because we measured IR absorption spectra of species isolated in *p*-H_2_, the spectra exhibit reliable relative IR intensities, which are critical for comparison with quantum-chemical computations; other techniques such as IRMPD or Ar-tagging measured action spectra rather than direct absorption spectra. Additionally, the sharp spectral lines coupled with their distinct photolytic behaviors enable the identification of various isomers.^12^

Using these techniques, our laboratory has successfully reported the IR spectra of several protonated PANHs isolated in solid *p*-H_2_, including protonated quinoline (1-quinolinium cation, C_9_H_7_NH^+^)^27^ and protonated isoquinoline (2-isoquinolinium cation, *iso*-C_9_H_7_NH^+^).^29^ In addition, five isomers of quinolinyl radicals (C_9_H_7_NH and 3-, 4-, 7-, and 8-HC_9_H_7_N)^27^ and eight isomers of isoquinolinyl radicals (C_9_H_7_NH and 1-, 3-, 4-, 5-, 6-, 7-, and 8-*iso*-HC_9_H_7_N)^29^ were generated and characterized by their IR spectra. For isoquinoline, hydrogenation was observed at the N atom and all accessible C atom sites except the two bridging carbon atoms in the fused ring.

The next members of PANH are phenanthridine and acridine, which possess structures like three-ring PAH, phenanthrene, and anthracene, respectively, with one CH group in the central ring substituted by nitrogen. Analogous to quinoline, phenanthridine has been detected in meteorites and is thought to play a potential role in prebiotic chemistry.^20,30^ Phenanthridine constitutes the core structural unit of DNA-binding fluorescent dyes through intercalation. Despite its biological and astrochemical relevance, studies on protonated and monohydrogenated phenanthridine remain scarce.

In this work, we extended our investigations to phenanthridine and successfully generated protonated and monohydrogenated phenanthridine. IR spectral identification was achieved for protonated phenanthridine C_13_H_9_NH^+^ and six isomers of monohydrogenated phenanthridine (C_13_H_9_NH and 1-, 2-, 7-, 9-, and 10-HC_13_H_9_N) in solid *p*-H_2_. Tentative assignments were also proposed for the 3- and 6-HC_13_H_9_N isomers. The implication for the identification of the UIR bands is discussed.

## 2 Experiments

The procedures used to generate and isolate protonated and monohydrogenated species in solid *p*-H_2_ for IR spectral characterization have been described previously.^12,25–27^ The cryogenic matrix substrate consisted of a gold-plated copper plate cooled to 3.2 K, serving both as a deposition surface and an IR reflector for reflective absorption measurements. IR absorption spectra were recorded with a Fourier-transform infrared spectrometer (Bruker, VERTEX 80 V) equipped with a KBr beam splitter and an MCT (Hg–Cd–Te) detector cooled to 77 K. Typically, 1200 interferograms were acquired at a spectral resolution of 0.25 cm^–1^ after each experimental stage.

Gaseous mixtures of phenanthridine (C_13_H_9_N) in *p*-H_2_ was prepared by flowing *p*-H_2_ gas (flow rate ∼10 mmol h^–1^) over solid phenanthridine heated to ∼300 K. Phenanthridine (98%, Sigma-Aldrich) was used after gentle heating under vacuum to remove volatile impurities. The resulting C_13_H_9_N/*p*-H_2_ vapor mixture was deposited onto the Cu substrate over a period of 7–8 h. During deposition, the matrix was subjected to electron bombardment by using an electron beam with a current of 15 μA and an energy of 300 eV, inducing protonation and hydrogenation reactions. To resolve the various products generated through electron bombardment, the matrix was maintained in the dark for ∼10 h, followed by secondary photolysis to selectively alter photolabile species.

Alternatively, monohydrogenated phenanthridine radicals were generated in C_13_H_9_N/Cl_2_/*p*-H_2_ matrices via UV photolysis at 365 nm, using a light-emitting diode (3 W) for 1 h, followed by IR irradiation with an external globar source for 2 h. To probe the photolytic behaviors and distinguish different groups of vibrational features, secondary photolysis was carried out at 445 nm (1.2 ± 0.2 mJ), 425 nm (1.2 ± 0.2 mJ), 410 nm (1.2 ± 0.2 mJ), 402 nm (1.2 ± 0.2 mJ), 380 nm (0.7 ± 0.1 mJ), 360 nm (0.7 ± 0.1 mJ), and 315 nm (0.7 ± 0.1 mJ), each for 20 min, using an optical parametric oscillator laser (EKSPLA, NT340, 10 Hz). To prevent undesired reactions between Cl and *p*-H_2_ during the acquisition of IR spectra, an IR long-pass filter with a cutoff wavelength of 2.4 μm was employed.

Due to the low vapor pressure of phenanthridine and the mixing procedure employed, the mixing ratio of C_13_H_9_N/*p*-H_2_ could not be accurately determined from partial pressures. Instead, we estimated the mixing ratios using the method of Tam and Fajardo^31^ based on the calculated absorption coefficient ε, the observed integrated absorbance of specific lines, and the IR absorption path length *l* through solid *p*-H_2_. The value of *l* (∼0.7 mm) was derived from the pure rotational transition at 1167 cm^–1^ of the *p*-H_2_ matrix and from the interference fringes in the IR spectra.^32^ The uncertainty in the calculated IR absorption coefficient ε, consequently in the mixing ratio *x*, may be as large as a factor of 2. However, using several lines of the same molecules helped to reduce this error. From five intense bands of phenanthridine observed at 1492.7, 1239.9. 889.7, 768.1, and 720.5 cm^–1^, we estimated the mixing ratio of C_13_H_9_N in *p*-H_2_ to be ∼(132 ± 7) ppm in hydrogenation experiments and ∼(73 ± 8) ppm in electron bombardment experiments. The ratio of Cl_2_/*p*-H_2_ was approximately 1/2700; with higher mixing ratios of Cl_2_, *p*-H_2_ might be destroyed after H atoms were produced because of the large exothermicity of the reaction H + H.

## 3 Quantum-Chemical Calculations

Phenanthridine has 14 distinct sites for protonation or hydrogenation; the labeling of the sites is indicated in Figure 1. The carbon atoms on the fused ring junctions are labeled 4a, 6a, 10a, and 10b, while the remaining carbon atoms are numbered from 1 to 10, except that position 5 is a nitrogen atom. In this study, species protonated or hydrogenated at the N atom site are denoted as C_13_H_9_NH^+^ and C_13_H_9_NH, respectively, while those protonated or hydrogenated at the C atom sites of phenanthridine are designated as *n*-H^+^C_13_H_9_N and *n*-HC_13_H_9_N, respectively, in which *n* refers to the carbon-atom number. When referring to protonated or hydrogenated phenanthridine species without specifying an isomeric form, the generic formulas H^+^C_13_H_9_N and HC_13_H_9_N are used.

**Figure 1:**
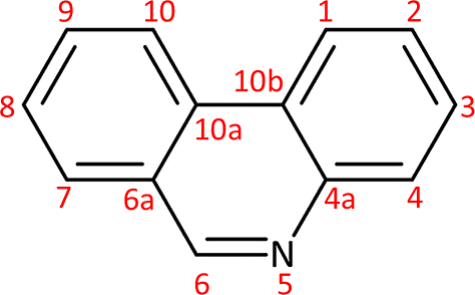
Numbering of carbon and nitrogen atoms in phenanthridine (C_13_H_9_N) according to the IUPAC nomenclature.

Quantum-chemical calculations were carried out using the Gaussian 16 (Rev. B.01) program package.^33^ Geometrical optimizations and vibrational analyses were performed with the B3LYP hybrid density functionals^34,35^ and the 6-311++G­(d,p) basis set.^36^ Harmonic vibrational wavenumbers of all species considered in this work were scaled according to the equations described in Section 4.1. Anharmonic vibrational wavenumbers and IR intensities were computed using second-order vibrational perturbation theory.^37^ For more accurate energetics, single-point energy calculations were performed using the method of coupled cluster with single, double, and perturbative triple excitations, CCSD­(T),^38^ at geometries optimized at the B3LYP/6-311++G­(d,p) level. Zero-point vibrational energies (ZPVEs) were corrected based on the harmonic vibrational wavenumbers calculated with the B3LYP method. Electronic excitation energies and oscillator strengths were evaluated by using time-dependent density-functional theory at the B3LYP/6-311++G­(d,p) level of theory.

### 3.1 Protonated Phenanthridine (Phenanthridinium Cations)

The geometries and Cartesian coordinates of isomers of protonated phenanthridine were predicted with the B3LYP/6-311++G­(d,p) method, and the relative energies were predicted with the CCSD­(T)/6-311++G­(d,p)//B3LYP/6-311++G­(d,p) method, as presented in Figure S1 and Table S1, respectively. The potential energy scheme for proton transfer to the neighboring sites is presented in Figure S2; energies from B3LYP calculations are listed in parentheses for comparison. Among all isomers, the isomer protonated at the N atom site of phenanthridine, 5-phenanthridinium (C_13_H_9_NH^+^), was predicted to have the least energy. The proton-transfer reactions from H_3_^+^ to phenanthridine, H_3_^+^ + C_13_H_9_N → H_2_ + HC_13_H_9_N^+^, are barrierless. The energy of C_13_H_9_NH^+^ + H_2_ is smaller than that of C_13_H_9_NH + H_3_^+^ by 555 kJ mol^–1^, implying a proton affinity of 977 kJ mol^–1^ for C_13_H_9_N at the N atom site according to CCSD­(T) calculations. In contrast, the energies of isomers protonated at the C atom sites of phenanthridine are at least 150 kJ mol^–1^ greater in energy than C_13_H_9_NH^+^; those protonated at the bridging C atom sites (4a-, 6a, 10a, and 10b) are ≥200 kJ mol^–1^ greater than C_13_H_9_NH^+^ (Table 1). For all isomers except 4a-, 6a, 10a, and 10b-H^+^C_13_H_9_N, all carbon and nitrogen atoms retain a planar ring structure with the *C*_s_ point-group symmetry; the ring skeleton of 4a-, 6a, 10a, and 10b-H^+^C_13_H_9_N is nonplanar with the *C*_1_ point-group symmetry. Although the energies of transition states for isomerization, 222–310 kJ mol^–1^ above C_13_H_9_NH^+^, are smaller than those of H_3_^+^ + C_13_H_9_N (555 kJ mol^–1^ relative to those of H_2_ + C_13_H_9_NH^+^), isomerization is unlikely once a product is stabilized in solid *p*-H_2_.

**Table 1:** Relative Energies of Various Isomers of Protonated Phenanthridine (*n*-H^+^C_13_H_9_N) and Transition States (TS) for Proton Transfer Calculated Using Two Methods

*n*-H^+^C_13_H_9_N		proton transfer		
site *n* ^a^	relative energy^b^ (kJ mol^–1^)	transfer	TS energy^c^ (kJ mol^–1^)	barrier (kJ mol^–1^)
1	155 (153)	1 → 2	226 (236)	71 (83)
2	158 (154)	2 → 3	248 (226)	90 (72)
3	160 (158)	3 → 4	222 (211)	62 (53)
4	150 (145)	4 → 4a	245 (237)	95 (92)
4a	214 (221)	4a → 5	310 (287)	96 (66)
5	0^d^ (0)^d^	5 → 6	273 (262)	273 (262)
6	197 (194)	6 → 6a	287 (275)	90 (81)
6a	215 (221)	6a → 7	270 (259)	55 (38)
7	186 (179)	7 → 8	245 (233)	59 (54)
8	168 (166)	8 → 9	270 (248)	102 (104)
9	194 (187)	9 → 10	248 (236)	54 (49)
10	164 (162)	10 → 10a	274 (260)	110 (98)
10a	237 (240)	10a → 10b	^e^	^e^
10b	202 (208)	10b → 1	235 (239)	33 (31)

a See Figure 1 for protonation sites.

b Energies relative to C_13_H_9_NH^+^ were calculated using the CCSD­(T)/6-311++G­(d,p)//B3LYP/6-311++G­(d,p) method; those obtained using the B3LYP/6-311++G­(d,p) method are listed in parentheses. ZPVEs are corrected using unscaled harmonic vibrational wavenumbers calculated with the B3LYP/6-311++G­(d,p) method.

c Energy of the transition state relative to that of C_13_H_9_NH^+^.

d The energy of C_13_H_9_NH^+^+H_2_ is smaller than that of H_3_^+^ + C_13_H_9_N by 555 or 556 kJ mol^–1^ according to CCSD­(T) or B3LYP calculations, respectively.

e The transition-state structure for the transfer 10a → 10b could not be located.

The scaled harmonic and anharmonic vibrational wavenumbers and IR intensities for C_13_H_9_N (Table S2), 1- to 4-H^+^C_13_H_9_N and C_13_H_9_NH^+^ (Table S3), 6- to 10-H^+^C_13_H_9_N (Table S4), 4a-, 6a-, 10a-, and 10b-H^+^C_13_H_9_N (Table S5) are provided. The scaling factors for harmonic vibrational wavenumbers are discussed in Section 4.1.

### 3.2 Monohydrogenated Phenanthridine (Phenanthridinyl Radicals)

The geometries and Cartesian coordinates of isomers of monohydrogenated phenanthridine (HC_13_H_9_N, phenanthridinyl radicals) were predicted using the B3LYP/6-311++G­(d,p) method and the relative energies using the CCSD­(T)/6-311++G­(d,p)//B3LYP/6-311++G­(d,p) method, as presented in Figure S3 and Table S6, respectively. The potential energy scheme depicting the formation of these isomers of HC_13_H_9_N from H + C_13_H_9_N (with transition states in blue) and hydrogen migration to the neighboring site (with transition states in red) among isomers is presented in Figure S4; energies from B3LYP calculations are listed in parentheses for comparison. The isomer with hydrogenation at the N atom site, the 5-phenanthridinyl radical (C_13_H_9_NH), was predicted to have the least energy, −117 kJ mol^–1^ relative to H + C_13_H_9_N according to CCSD­(T) calculations. Other isomers, 1- to 10-HC_13_H_9_N, are predicted to lie 25–45 kJ mol^–1^ above C_13_H_9_NH. Energies of 4a-, 6a-, 10a-, and 10b-HC_13_H_9_N are much greater, ranging from 79 to 89 kJ mol^–1^. All isomers, except for 4a-, 6a-, 10a-, and 10b-HC_13_H_9_N, exhibit planar ring structures and belong to the *C*_s_ point-group symmetry; 4a-, 6a-, 10a-, and 10b-HC_13_H_9_N have a nonplanar ring skeleton and belong to point group *C*_1_. All barrier heights for reactions H + C_13_H_9_N → HC_13_H_9_N fall within 24–33 kJ mol^–1^, except those for the formation of 4a-, 6a-, 10a-, and 10b-HC_13_H_9_N, which have barriers 40–63 kJ mol^–1^ (Table 2). Because the energies of TS for isomerization are greater than that of H + C_13_H_9_N, isomerization is unlikely to occur during hydrogenation of phenanthridine in the low-temperature solid *p*-H_2_ environment.

**Table 2:** Relative Energies of Various Isomers of Hydrogenated Phenanthridine (*n*-HC_13_H_9_N) and Transition States (TS) for Hydrogen Transfer Calculated Using Two Methods

*n*-HC_13_H_9_N			H transfer		
site *n* ^a^	relative energy^b^ (kJ mol^–1^)	barrier (kJ mol^–1^)	transfer	TS energy^c^ (kJ mol^–1^)	barrier (kJ mol^–1^)
1	26 (42)	26 (11)	1 → 2	198 (220)	172 (178)
2	39 (55)	31 (14)	2 → 3	189 (208)	150 (153)
3	45 (60)	32 (15)	3 → 4	199 (221)	154 (161)
4	27 (39)	27 (12)	4 → 4a	254 (259)	227 (220)
4a	87 (118)	45 (34)	4a → 5	228 (239)	141 (121)
5	0^d^ (0)^d^	25 (8)	5 → 6	190 (206)	190 (206)
6	28 (43)	24 (14)	6 → 6a	190 (203)	147 (160)
6a	89 (119)	63 (33)	6a → 7	250 (258)	161 (139)
7	29 (43)	29 (13)	7 → 8	226 (225)	197 (182)
8	44 (60)	33 (15)	8 → 9	207 (208)	163 (148)
9	41 (55)	32 (15)	9 → 10	212 (219)	171 (164)
10	25 (40)	26 (12)	10 → 10a	209 (220)	184 (180)
10a	87 (113)	43 (32)	10a → 10b	^e^	^e^
10b	79 (106)	40 (30)	10b → 1	217 (225)	138 (119)

a See Figure 1 for hydrogenation sites.

b Energies relative to C_13_H_9_NH were calculated using the CCSD­(T)/6-311++G­(d,p)//B3LYP/6-311++G­(d,p) method; those obtained using the B3LYP/6-311++G­(d,p) method are listed in parentheses. ZPVEs are corrected using unscaled harmonic vibrational wavenumbers calculated with the B3LYP/6-311++G­(d,p) method.

c Energy of the transition state relative to that of C_13_H_9_NH.

d The energy of C_13_H_9_NH is smaller than that of H + C_13_H_9_N by 117 or 161 kJ mol^–1^ according to CCSD­(T) or B3LYP methods, respectively.

e The transition-state structure for 10a → 10b could not be located.

The scaled harmonic and anharmonic vibrational wavenumbers and IR intensities for 1- to 4-HC_13_H_9_N and C_13_H_9_NH (Table S7), 6- to 10-HC_13_H_9_N (Table S8), 4a-, 6a-, 10a-, and 10b-HC_13_H_9_N (Table S9) are provided.

## 4 Experimental Results

### 4.1 IR Spectrum of C_13_H_9_N/*p*-H_2_ Matrices and Scaling of Vibrational Wavenumbers

An IR spectrum of a C_13_H_9_N/*p*-H_2_ matrix is shown in Figure S5a. Prominent features were observed at 1595.2, 1492.7, 1239.9, 889.7, 768.1, 748.7, and 720.5 cm^–1^, with the line at 748.7 cm^–1^ being the most intense. The observed line positions agree well with those reported for C_13_H_9_N in solid Ar (Table S2).^16^ The observed wavenumbers were plotted against harmonic vibrational wavenumbers predicted with the B3LYP/6-311++G­(d,p) method in Figure S6a. A linear regression yielded a scaling equation *y* = (0.9510 ± 0.0021) *x* + (35.9 ± 3.5), in which *y* is the scaled harmonic vibrational wavenumber and *x* is the computed harmonic vibrational wavenumber; errors present one standard deviation in fitting. For improved accuracy in the region below 2000 cm^–1^, a second regression was performed using only data in region 600–2000 cm^–1^ (Figure S6b), yielding *y* = (0.9778 ± 0.0019) *x* + (6.3 ± 2.3). Accordingly, for all species considered in this work, this refined equation was applied to scale harmonic vibrational wavenumbers below 2000 cm^–1^, while the former equation *y* = 0.9510 *x* + 35.9 was used for wavenumbers above 2000 cm^–1^.

Simulated stick spectra based on harmonic, scaled harmonic, and anharmonic vibrational wavenumbers are illustrated in Figures S5b–d, respectively. A summary of the observed and computed wavenumbers, along with relative IR intensities, is provided in Table S2. The mean absolute deviation between observed and scaled harmonic vibrational wavenumbers is 4.4 ± 5.8 cm^–1^, while that for anharmonic vibrational predictions is 7.9 ± 8.3 cm^–1^, indicating satisfactory agreement between the experiment and theory.

### 4.2 IR Spectra of Electron-Bombarded C_13_H_9_N/*p*-H_2_ Matrices

Figure 2 shows IR spectra after various experimental steps of an electron-bombarded C_13_H_9_N/*p*-H_2_ matrix in representative spectral regions. Figure 2a presents the spectrum of C_13_H_9_N in solid *p*-H_2_ as a reference. Figure 2b shows the spectrum acquired after 7 h of matrix deposition under electron bombardment in a separate experiment. Figure 2c shows the difference spectrum after maintaining this matrix in darkness for 14 h; positive features indicate generation, while negative ones indicate depletion. It has been demonstrated that after maintenance of the matrix in darkness for a prolonged period, the intensities of lines associated with protonated species decreased due to neutralization of the cations by electrons trapped in the matrix.^12^ Concurrently, the intensities of hydrogenated species increased because of the neutralization of protonated species and reactions between residual hydrogen atoms and C_13_H_9_N. Following this step, the matrix was irradiated sequentially at 547, 525, 457, 420, and 336 nm for 20 min each. Difference spectra after each secondary irradiation are shown in Figure 2d–h.

**Figure 2:**
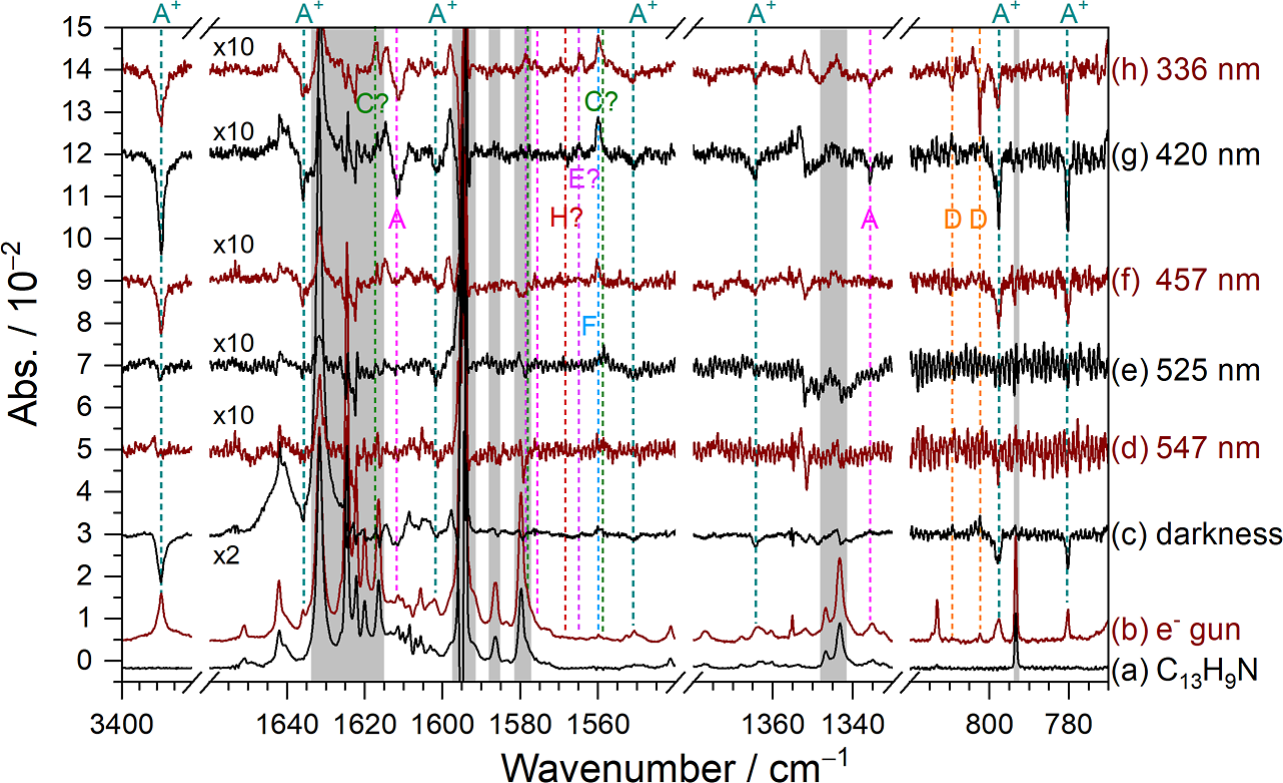
Representative IR spectra of an electron-bombarded C_13_H_9_N/*p*-H_2_ matrix at various stages of the experiment. (a) Spectrum of a C_13_H_9_N/*p*-H_2_ mixture. (b) Spectrum of a C_13_H_9_N/*p*-H_2_ mixture upon electron bombardment during deposition at 3.2 K for 7 h in a separate experiment. (c) Difference spectra of the electron-bombarded C_13_H_9_N/*p*-H_2_ matrix after maintenance in darkness at 3.2 K for 14 h. Difference spectra of the matrix upon sequential secondary irradiation at 547 nm (d), 525 nm (e), 457 nm (f), 420 nm (g), and 336 nm (h), each for 20 min. Lines in group A^+^ (C_13_H_9_NH) are indicated with green dashed lines and labels. Other lines attributable to isomers of HC_13_H_9_N (groups A–H) are also indicated. Spectral regions subjected to interference from the intense absorption of C_13_H_9_N are shaded gray. Baselines were shifted for clarity.

Based on the photolytic behavior, several groups of lines (groups A^+^ and A–H) were identified. The intensities of features in group A^+^ decreased after the 14 h darkness period (Figure 2c) and further attenuated by ∼0, 2, 9, 14, and 9% after irradiation at 547, 525, 457, 420, and 336 nm, respectively (Table 3); the percentage variation is relative to the spectrum measured before each step. Lines in group A^+^ include prominent features observed at 3388.9, 797.6, and 780.2 cm^–1^ and weaker ones at 1635.9, 1601.9, 1550.9, and 1364.2 cm^–1^. These vibrational wavenumbers with integrated relative intensities are listed in Table 4. As discussed in Section 5.1, these lines in group A^+^ are assigned to the protonated species C_13_H_9_NH^+^ (5-phenanthridinium cation).

**Table 3:** Mixing Ratios (in ppb) of C_13_H_9_NH^+^ after Secondary Photolysis at Various Wavelengths

group	assignment	deposition	547 nm	525 nm	457 nm	420 nm	336 nm
A^+^	C_13_H_9_NH^+^	560 ± 184	560 ± 180 (∼0%)^a^	550 ± 180 (−2%)	500 ± 160 (−9%)	430 ± 135 (−14%)*^b^	390 ± 130 (−9%)

a Percentage variations in parentheses are relative to the mixing ratios measured in the previous stage.

b The most significant percentage variation among all wavelengths is marked with *.

**Table 4:** Comparison of Observed Vibrational Wavenumbers (in cm^–1^) and Relative IR Intensities of Lines in Group A^+^ with the Scaled Harmonic and Anharmonic Vibrational Wavenumbers and IR Intensities of C_13_H_9_NH^+^ Predicted Using the B3LYP/6-311++G­(d,p) Method

		C_13_H_9_NH^+^ (group A^+^)		
mode^a^	sym	scaled harmonic^b^	anharmonic	*p*-H_2_
ν_1_	a′	3420 (149)^c^	3395 (127)^c^	3388.9 (100)^d^
ν_11_	a′	1632 (52)	1622 (18)	1635.9 (39)
ν_12_	a′	1620 (93)	1614 (29)	^e^
ν_13_	a′	1610 (14)	1608 (5)	1601.9 (15)
ν_15_	a′	1557 (26)	1549 (15)	1550.9 (13)
ν_17_	a′	1479 (25)	1477 (9)	^e^
ν_19_	a′	1446 (18)	1444 (4)	^e^
ν_21_	a′	1371 (46)	1363 (34)	^f^
ν_22_	a′	1362 (45)	1352 (15)	1364.2^f^ (25)
ν_26_	a′	1247 (21)	1246 (16)	^e^
ν_53_	a″	792 (127)	838 (4)	797.6 (75)
ν_54_	a″	777 (33)	776 (105)	780.2 (77)
ν_55_	a″	754 (17)	760 (18)	^e^
ν_57_	a″	706 (20)	688 (24)^e^	

a Modes with harmonic IR intensities less than 15 km mol^–1^ are not listed unless it was observed.

b Harmonic vibrational wavenumbers scaled according to 0.9510*x* + 35.9 for wavenumbers >2000 cm^–1^ and 0.9778*x* + 6.3 for wavenumbers <2000 cm^–1^.

c IR intensities in km mol^–1^.

d IR intensities as a percentage of the most intense line observed at 3388.9 cm^–1^.

e Interference due to absorption of the parent.

f Interaction of ν_21_ and ν_22_ might be extensive.

Lines in group A–H increased in intensity after prolonged dark storage; they are attributed to hydrogenated species C_13_H_9_NH (phenanthridinyl radicals). Because these features were weak in electron-bombardment experiments and more distinct in experiments with C_13_H_9_N/Cl_2_/*p*-H_2_ matrices, the corresponding data in the latter experiments (Section 4.3) are used for spectral assignments elaborated on in Sections 5.2–5.6.

### 4.3 IR Spectra of UV/IR-Irradiated C_13_H_9_N/Cl_2_/*p*-H_2_ Matrices

The IR spectrum recorded after deposition of a C_13_H_9_N/Cl_2_/*p*-H_2_ matrix for 7 h is shown in Figure S7a. Spectra obtained following UV photolysis of the matrix at 365 nm for 1 h and subsequent IR irradiation for 2 h are presented in Figure S7b,c, respectively. Photolysis at 365 nm generated Cl atoms, as evidenced by the spin–orbit transition of Cl at 943.8 cm^–1^ (ref 39) and a ClOO feature at 1441.1 cm^–1^,^40^ originating from the reaction of Cl with a trace O_2_ impurity. No significant reaction between Cl and phenanthridine was observed. In contrast, subsequent IR irradiation produced numerous new features, including absorptions from HCl, HCl clusters, and HO_2_.

Representative IR spectra in the 772–670 cm^–1^ region after each experimental step are shown in Figure 3; corresponding spectra across 3500–3450, 3150–2780, and 1650–550 cm^–1^ regions are provided in Figure S8. The spectra after irradiation at 365 nm are presented in Figures 3a and S8a, while the difference spectra following IR irradiation are presented in Figures 3b and S8b. Newly appearing positive features are attributed to products of H + C_13_H_9_N. To classify these newly observed features, secondary irradiation was performed sequentially at 445, 425, 410, 402, 380, 360, and 315 nm, each for 20 min. The resulting difference spectra after each step are shown in Figures 3c–i and S8c–i.

**Figure 3:**
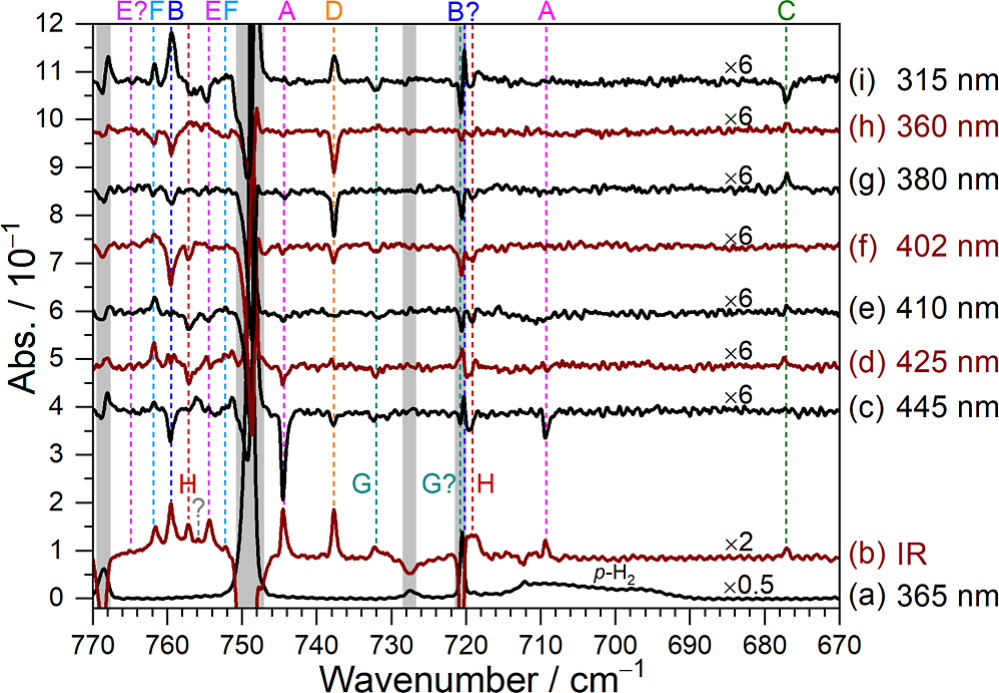
IR spectra of a C_13_H_9_N/Cl_2_/*p*-H_2_ matrix in regions 670–772 cm^–1^ at various stages of the experiment. (a) Spectrum of a C_13_H_9_N/Cl_2_/*p*-H_2_ matrix deposited at 3.2 K for 7 h and irradiated at 365 nm for 1 h. (b) Difference spectrum of the matrix after subsequent irradiation with IR light for 2 h. Difference spectrum of the matrix after further sequential irradiation at 445 nm (c), 425 nm (d), 410 nm (e), 402 nm (f), 380 nm (g), 360 nm (h), and 315 nm (i); each step is 20 min. Lines in groups A (C_13_H_9_NH), B (9-HC_13_H_9_N), C (1-HC_13_H_9_N), D (2-C_13_H_9_N), E (10-C_13_H_9_N), F (7-C_13_H_9_N), G (3-C_13_H_9_N), and H (6-C_13_H_9_N) are indicated with color-coded dashed lines and labels. Spectral regions subjected to interference from the intense absorption of C_13_H_9_N are shaded gray. Baselines were shifted for clarity.

Based on the distinct responses during secondary photolysis at varied wavelengths, the newly observed features following IR irradiation were categorized into eight groups, designated as A to H. The mixing ratios were derived from the integrated absorbance of lines listed in Table S10, and the average mixing ratios of each group across experimental steps are summarized in Table 5; percentage changes listed parenthetically indicate variations relative to the preceding step. For group A, lines decreased at all wavelengths, except 315 nm. Lines in groups B and D showed comparable trends; they showed no change at 425 and 410 nm, decreased at 445, 402, 380, and 360 nm with different proportional changes, but increased at 315 nm. Lines in group C increased at 425, 380, and 360 nm but decreased at 315 nm, while lines in group E decreased only at 315 nm and increased at 360 nm. Lines in group F increased at most wavelengths except 380 and 360 nm, but a nearly inverse behavior was observed for lines in groups G and H, except that they remained nearly unchanged at 380 nm.

**Table 5:** Mixing Ratios of Various Isomers of HC_13_H_9_N after Secondary Photolysis at Various Wavelengths

group	assignment	mixing ratio^a^	445 nm	425 nm	410 nm	402 nm	380 nm	360 nm	315 nm
A	C_13_H_9_NH	2.7 ± 0.4	1.00 ± 0.11	0.59 ± 0.08	0.35 ± 0.08	0.24 ± 0.08	0.22 ± 0.05	0.11 ± 0.08	0.11 ± 0.08
			(−63%)*^b^ ^,^ ^c^	(−41%)	(−41%)	(−31%)	(−11%)	(−50%)	(∼0%)
B	9-HC_13_H_9_N	2.2 ± 0.1	1.50 ± 0.13	1.50 ± 0.13	1.50 ± 0.13	0.68 ± 0.13	0.46 ± 0.09	0.04 ± 0.09	1.32 ± 0.13
			(−32%)	(∼0%)	(∼0%)	(−54%)	(−32%)	(−90%)*	[2900%]
C	1-HC_13_H_9_N	2.3 ± 0.2	2.3 ± 0.2	3.2 ± 0.3	3.2 ± 0.3	3.2 ± 0.3	4.4 ± 0.4	4.8 ± 0.4	3.3 ± 0.3
			(∼0%)	[40%]	(∼0%)	(∼0%)	[35%]	[11%]	(−31%)*
D	2-HC_13_H_9_N	2.3 ± 0.5	2.0 ± 0.4	2.0 ± 0.4	2.0 ± 0.4	1.6 ± 0.3	0.76 ± 0.21	0.16 ± 0.12%	0.64 ± 0.07
			(−13%)	(∼0%)	(∼0%)	(−20%)	(−53%)	(−79%)*	[300%]
E	10-HC_13_H_9_N	1.9 ± 1.0	2.0 ± 1.0	2.0 ± 1.0	2.0 ± 1.0	2.0 ± 1.0	2.0 ± 1.0	3.1 ± 1.4	1.2 ± 0.6
			[5%]	(∼0%)	(∼0%)	(∼0%)	(∼0%)	[57%]	(−62%)*
F	7-HC_13_H_9_N	2.3 ± 0.3	2.7 ± 0.3	3.7 ± 0.4	4.3 ± 0.5	4.9 ± 0.5	4.4 ± 0.5	4.0 ± 0.4	5.5 ± 0.6
			[19%]	[35%]	[16%]	[14%]	(−10%)*	(−8%)	[37%]
G	3-HC_13_H_9_N ?^d^	1.6 ± 0.6	1.28 ± 0.48	0.98 ± 0.35	0.86 ± 0.36	0.24 ± 0.08	0.24 ± 0.08	0.67 ± 0.28	0.03 ± 0.05
			(−20%)	(−24%)	(−11%)	(−72%)	(∼0%)	[180%]	(−95%)*
H	6-HC_13_H_9_N ?^d^	1.4	1.4	0.87	0.43	0.11	0.11	0.32	0.00
			(0%)	(−38%)	(−50%)	(−74%)	(∼0%)	[188%]	(−100%)*

a In ppm; see the text.

b The percentage variations are relative to the mixing ratios measured in the previous stage. Decreases are in parentheses, and increases are in square brackets.

c For each species, the most significant percentage decrease among all wavelengths is marked with *.

d Tentative assignments; see the text.

These group-specific features are highlighted using color-coded labels and dashed lines in Figures 3 and S8. Observed wavenumbers and relative intensities of lines in groups A–H are listed in Tables 6–9. These spectral features in groups A–H are assigned to C_13_H_9_NH and 9-, 1-, 2-, 10-, 7-, 3-, and 6-HC_13_H_9_N, respectively, to be discussed in Sections 5.2–5.5. The estimated mixing ratios are [C_13_H_9_N] = (132 ± 7) ppm, [C_13_H_9_NH] = (2.7 ± 0.4) ppm, [9-HC_13_H_9_N] = (2.2 ± 0.1) ppm, [1-HC_13_H_9_N] = (2.3 ± 0.2) ppm, [2-HC_13_H_9_N] = (2.3 ± 0.5) ppm, [10-HC_13_H_9_N] = (1.9 ± 1.0) ppm, [7-HC_13_H_9_N] = (2.3 ± 0.3) ppm, [3-HC_13_H_9_N] = (1.6 ± 0.6) ppm, and [6-HC_13_H_9_N] ≈ 1.4 ppm; listed errors represent one standard deviation among measurements from various lines.

## 5 Discussion

### 5.1 Assignment of Lines in Group A^+^ to 5-Phenanthridinium Cation (C_13_H_9_NH^+^)

The intensities of the lines in group A^+^ decayed during maintenance in darkness, suggesting that the most likely carrier of lines in this group is a phenanthridinium cation (protonated phenanthridine) (Figure 2g,h). IR spectra of the electron-bombarded matrix recorded after irradiation at 420 and 336 nm, respectively, are reproduced in Figure 4a. At these two wavelengths, features in group A^+^ revealed the most significant reduction in the intensities. These features are annotated with dashed lines and labels; spectral regions subjected to interference from the intense absorption of the precursor (C_13_H_9_N) are shaded gray. To identify the carrier, stick spectra of ten isomers of H^+^C_13_H_9_N, simulated based on scaled harmonic vibrational wavenumbers and IR intensities, are presented in Figure 4b–k. Comparative analysis reveals that the observed A^+^ features align, in terms of wavenumbers and relative intensities, most closely with predicted bands for C_13_H_9_NH^+^, as detailed in Table 4. In contrast, poor agreement is found with the spectra simulated for isomers of H^+^C_13_H_9_N protonated at a carbon site (Tables S3–S5).

**Figure 4:**
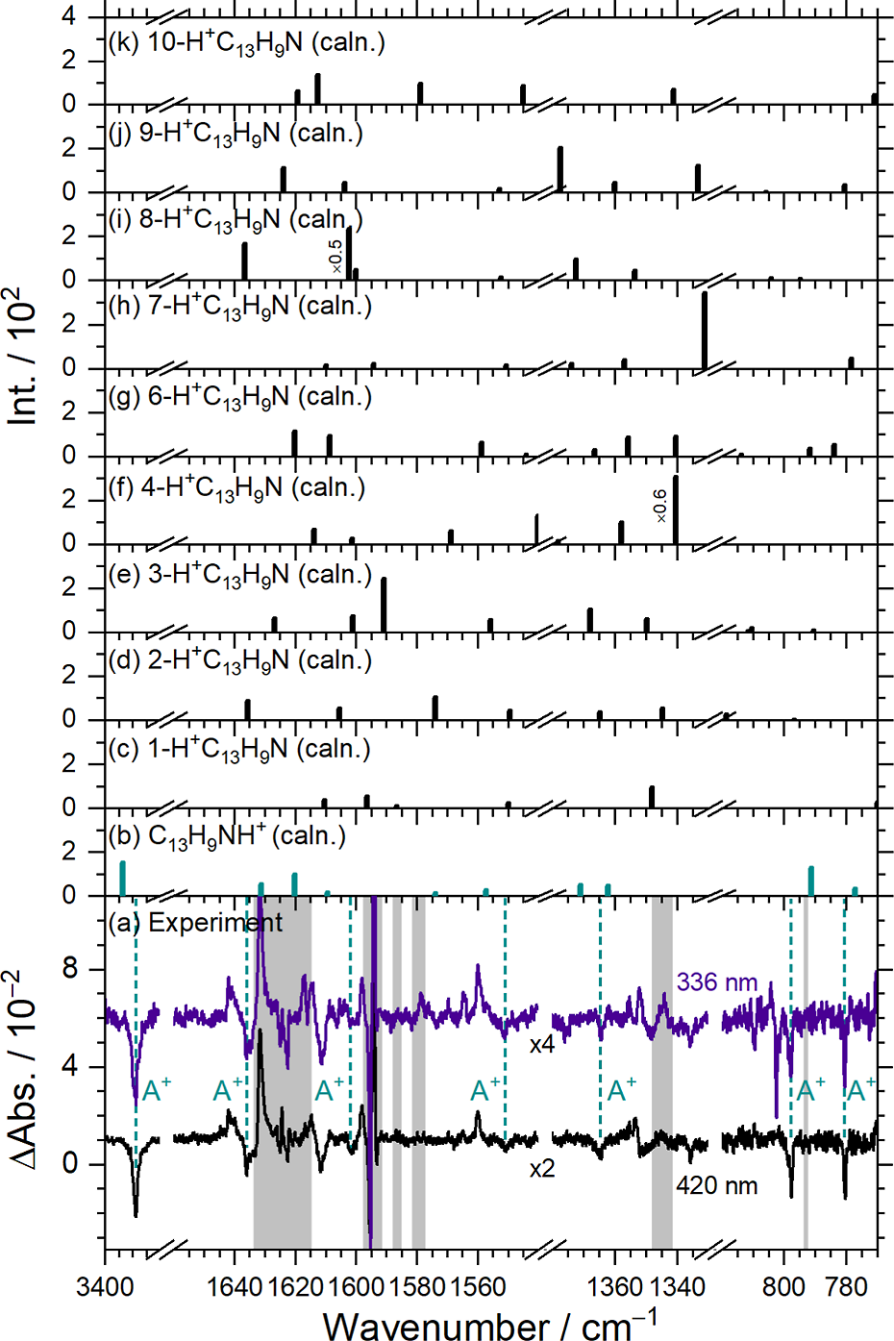
Comparison of observed lines in group A^+^ with the theoretically predicted stick spectra of isomers of protonated phenanthridine (HC_13_H_9_N^+^). (a) Difference spectra after secondary photolysis at 420 nm (lower trace) and 336 nm (upper trace); lines in group A^+^ are indicated with green dashed lines and labels. Baselines were shifted for clarity. IR stick spectra of C_13_H_9_NH^+^ (b), 1-H^+^C_13_H_9_N (c), 2-H^+^C_13_H_9_N (d), 3-H^+^C_13_H_9_N (e), 4-H^+^C_13_H_9_N (f), 6-H^+^C_13_H_9_N (g), 7-H^+^C_13_H_9_N (h), 8-H^+^C_13_H_9_N (i), 9-H^+^C_13_H_9_N (j), and 10-H^+^C_13_H_9_N (k), simulated according to scaled harmonic wavenumbers and IR intensities predicted with the B3LYP/6-311++G­(d,p) method. Spectral regions subjected to interference from the intense absorption of C_13_H_9_N are shaded gray.

The most intense feature observed at 3388.9 cm^–1^ is characteristic of the N–H stretching mode and match with the scaled harmonic vibrational wavenumber 3420 cm^–1^ (with intensity 149 km mol^–1^) and the anharmonic vibrational wavenumber at 3395 cm^–1^ for the N–H stretching mode of C_13_H_9_NH^+^. This mode serves as a clear vibrational signature of the N-protonated species. Characteristic for protonated PAH and PANH, the C–H stretching modes of C_13_H_9_NH^+^ exhibit low IR intensities (<5 km mol^–1^) and are masked by much more intense precursor absorptions, complicating direct spectral assignment. Among the C-protonated isomers, except 4a-, 6a-, 10a-, and 10b-H^+^C_9_H_7_N, the C–H stretching modes of C_13_H_9_NH^+^ are weak and only the CH_2_ symmetric stretching mode, predicted in the region 2847–2873 cm^–1^, has intensity 16–64 km mol^–1^. However, the absence of new features in the 2650–2900 cm^–1^ region that were induced by electron bombardment and decayed after maintenance in darkness supports the exclusion of these C-protonated isomers from contributing to group A^+^ features.

In the spectral region below 1700 cm^–1^, two intense lines were observed at 797.6 and 780.2 cm^–1^, near the scaled harmonic wavenumbers predicted for C_13_H_9_NH^+^ at 792 and 777 (ν_53_ and ν_54_, both out-of-plane C–H and N–H bend) cm^–1^, respectively. Although the observed intensities of these two lines are comparable, harmonic calculations suggested that ν_53_ should be about four times more intense than ν_54_. In contrast, anharmonic vibrational calculations predict a significant blue shift of ν_54_ by 46 cm^–1^ but with its IR intensity 1/26 of ν_53_, suggesting a strong vibrational coupling between these two modes, even though the anharmonic interaction might have been overestimated. Additional weaker lines were observed at 1635.9, 1601.9, 1550.9, and 1364.2 cm^–1^, corresponding well to 1632 (ν_11_), 1610 (ν_13_, both C=C and C=N stretch), 1557 (ν_15_, C=C stretch), and 1362 (ν_22_, ring deform) cm^–1^, respectively. Two other vibrational modes of C_13_H_9_NH^+^ near 1620 (ν_12_) and 1371 (ν_21_) cm^–1^ were predicted to have relatively large IR intensities (93 and 46 km mol^–1^, respectively), yet could not be definitively identified due to overlapping absorption from the parent molecule, C_13_H_9_N. The mean absolute deviation between observed and scaled harmonic vibrational wavenumbers is 8.6 ± 10.1 cm^–1^, whereas the deviation between observed and anharmonic vibrational wavenumbers is 12 ± 13 cm^–1^ because of the large deviation of ν_53_. If we take out the large deviation of ν_1_ (typical for the NH-stretching mode), the mean absolute deviation between observed and scaled harmonic vibrational wavenumbers becomes 4.9 ± 2.2 cm^–1^. Overall, the spectral correlations affirm the assignment of group A^+^ features to the N-protonated phenanthridine isomer, C_13_H_9_NH^+^.

### 5.2 Assignments of Lines in Groups A and F to C_13_H_9_NH and 7-HC_13_H_9_N Radicals

As discussed in Section 4.3, the IR lines that emerged after IR irradiation of a UV-irradiated C_13_H_9_N/Cl_2_/*p*-H_2_ matrix were classified into eight distinct groups based on their photolytic behavior upon irradiation at various wavelengths. After UV/IR irradiation, H atoms were expected to be produced and subsequently reacted with C_13_H_9_N to form hydrogenated species rather than protonated species. These features were also produced upon electron bombardment and intensified with prolonged maintenance of an electron-bombarded C_13_H_9_N/*p*-H_2_ matrix in darkness because of the neutralization of H^+^C_13_H_9_N by trapped electrons and reactions of residual H atoms with C_13_H_9_N. However, the intensities of these features are weak. We hence used data from the UV/IR-irradiated C_13_H_9_N/Cl_2_/*p*-H_2_ matrix to discuss the spectral assignments of each group as follows.

The difference spectra for lines in groups A and F, obtained following irradiation at 425 and 445 nm, are presented in Figure 5b over the region 970–670 cm^–1^. These are compared with the predicted IR stick spectra of C_13_H_9_NH and 7-HC_13_H_9_N, shown in Figure 5a,c, respectively. IR features in group A are indicated with pink dashed lines and labels, while those in group F are marked with light blue dashed lines and labels. Additional similar comparisons across other spectral regions are shown in Figure S9, and comparisons of observed lines in Groups A and F with predicted IR stick spectra of other isomers of HC_13_H_9_N are presented in Figure S10.

**Figure 5:**
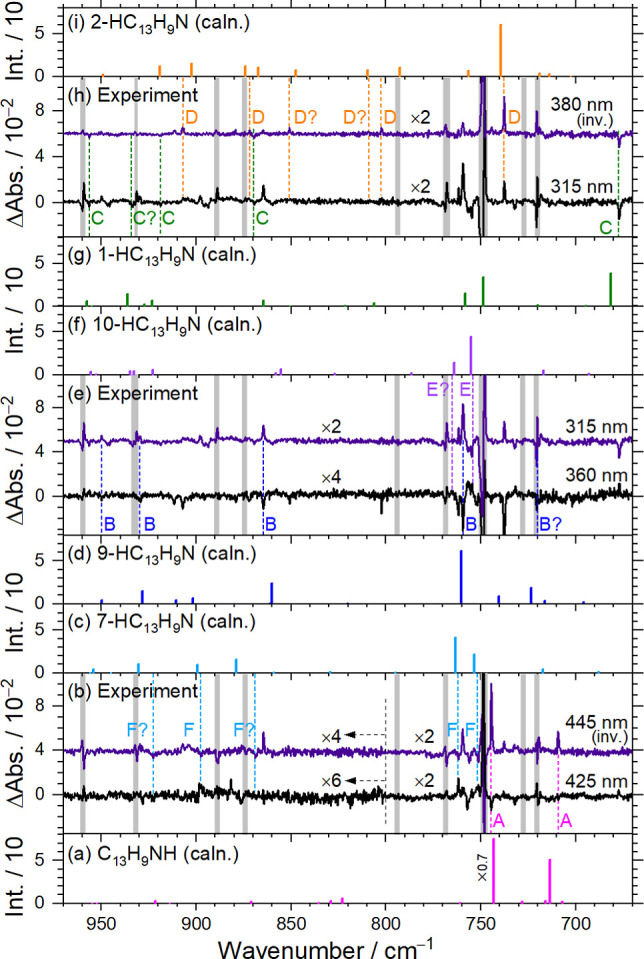
Comparison of observed lines of groups A–F in the C_13_H_9_N/Cl_2_/*p*-H_2_ experiments in region 670–970 cm^–1^ with theoretically predicted stick spectra of assigned isomers of hydrogenated phenanthridine (HC_13_H_9_N). (a) IR stick spectrum of 7-HC_13_H_9_NH. (b) Difference spectra after secondary photolysis at 425 nm (lower trace, from Figure 3d) and after secondary photolysis at 445 nm (upper trace, from Figure 3c, inverted); lines of groups A and F are indicated with pink and light blue dashed lines and labels. (C) IR stick spectrum of C_13_H_9_NH. (d) IR stick spectrum of 9-HC_13_H_9_NH. (e) Difference spectra after secondary photolysis at 360 nm (lower trace, from Figure 3h) and after secondary photolysis at 315 nm (Figure 3i); lines of groups B and E are indicated with blue and purple dashed lines and labels. (f) IR stick spectrum of 10-HC_13_H_9_N. (g) IR stick spectrum of 1-HC_13_H_9_NH. (h) Difference spectra after secondary photolysis at 315 nm (lower trace, from Figure 3i) and after secondary photolysis at 380 nm (Figure 3g); lines of groups C and D are indicated with green and orange dashed lines and labels. (i) IR stick spectrum of 2-HC_13_H_9_N. All IR stick spectra are based on the scaled harmonic vibrational wavenumbers and IR intensities predicted using the B3LYP/6-311++G­(d,p) method. The predicted IR intensities are in km mol^–1^. Spectral regions subjected to interference by the intense absorption of C_13_H_9_N are shaded gray (single column).

Features in group A with significant intensity were observed at 3473.8, 1508.2, 1473.4, 1443.2, 1414.9, 1306.0, 1283.9, 992.4, 744.6, and 709.4 cm^–1^, in satisfactory agreement with the predicted spectrum of C_13_H_9_NH, which shows intense lines at 3500 (ν_1_, NH stretch), 1510 (ν_16_), 1468 (ν_17_), 1437 (ν_19_), 1415 (ν_20_), 1306 (ν_22_), 1282 (ν_24_), 989 (ν_35_), 743 (ν_53_), and 714 cm^–1^ (ν_55_), shown in Figures 5c and S9c. In addition to these prominent features, ten weaker lines in group A were also assigned; five further lines were tentatively attributed to C_13_H_9_NH but suffer from severe interference by the parent or other products, as indicated in Table 6, which lists vibrational modes having predicted harmonic IR intensities greater than 10 km mol^–1^. Simulated spectra of other isomers of phenanthridinyl radicals failed to reproduce the wavenumbers and relative intensities of lines in group A satisfactorily (Figures 5 and S9, S10), reinforcing the assignment of group A features to C_13_H_9_NH. Most lines predicted for C_13_H_9_NH have been observed, including its characteristic N–H stretching mode (ν_1_). A weaker line at 3476.1 cm^–1^ was also observed, likely due to the matrix-site effect arising from the varied interactions between the NH moiety in C_13_H_9_NH and H_2_. The mean absolute deviation between the observed wavenumbers and scaled harmonic vibrational wavenumbers was 3.9 ± 5.1 cm^–1^, while that for the anharmonic calculations was 5.0 ± 3.5 cm^–1^, both indicating satisfactory agreement between the experiment and theory.

Assignment of lines in group F to the 7-HC_13_H_9_N radical is discussed as follows. Group F lines observed in the spectral region below 1650 cm^–1^ include prominent features at 1560.0, 1489.0, 1291.6, 1275.9, 897.5, 761.7, 752.3, and 663.8 cm^–1^, in satisfactory agreement with lines of 7-HC_13_H_9_N predicted near 1567 (ν_11_), 1491 (ν_14_), 1296 (ν_22_), 1281 (ν_23_), 899 (ν_51_), 763 (ν_54_), 754 (ν_55_), and 662 (ν_56_). Nine additional, weaker features were tentatively assigned to 7-HC_13_H_9_N but suffer from the interference from the parent C_13_H_9_N or other products; they are marked with ? in Table 7. In regions 600–1600 cm^–1^, all lines of 7-HC_13_H_9_N predicted to have IR intensities >10 km mol^–1^ have been observed in group F. In contrast, mode assignments in the CH-stretching region proved challenging due to two compounding factors: severe overlap with intense absorption bands of the parent species and significant anharmonic coupling. Nonetheless, one feature at 2832.3 cm^–1^ was tentatively attributed to the characteristic symmetric CH_2_-stretching (ν_9_) mode of 7-HC_13_H_9_N. The line predicted at 2841 cm^–1^ (2786 cm^–1^ from anharmonic vibrational calculations) for the anti-symmetric CH_2_-stretching (ν_45_) mode could not be discerned, owing to its weak intensity and interference from the intense absorption of HCl and its complexes.

**Table 6:** Comparison of Observed Vibrational Wavenumbers (in cm^–1^) and Relative IR Intensities of Lines in Groups A and B with Scaled Harmonic and Anharmonic Vibrational Wavenumbers and IR Intensities of C_13_H_9_NH and 9-HC_13_H_9_N Predicted Using the B3LYP/6-311++G­(d,p) Method

		C_13_H_9_NH (group A)						9-HC_13_H_9_N (group B)					
mode^a^	sym	harmonic^b^		anharmonic		experiment		harmonic^b^		anharmonic		experiment	
ν_1_	a′	3500	(60)^c^	3468	(43)^c^	3473.8	(38)^d^	3076	(15)^c^	3128	(5)^c^	^f^	
						3476.1	(8)						
ν_2_	a′	3100	(7)	3097	(7)	3100.8?	^e^	3071	(20)	3061	(16)	3071.1?	^e^
ν_3_	a′	3080	(18)	3084	(15)	3086.0?	^e^						
ν_4_	a′	3072	(19)	3073	(33)	3076.0?	^e^						
ν_5_	a′							3053	(31)	3040	(1)	3046.9?	^e^
ν_6_	a′	3058	(28)	3053	(25)	3058.3?	^e^						
ν_7_	a′	3053	(15)	3047	(0)	^f^							
ν_8_	a′							3001	(26)	2989	(20)	^f^	
ν_9_	a′							2824	(41)	2814	(16)	2799.1?	(82)^d^
ν_11_	a′	1608	(25)	1597	(5)	1612.0	(3)						
ν_13_	a′	1580	(25)	1575	(8)	1576.3?	(5)						
ν_14_	a′							1478	(21)	1471	(14)	1476.8	(29)
ν_16_	a′	1510	(41)	1502	(32)	1508.2	(51)						
ν_17_	a′	1468	(56)	1465	(30)	1473.4	(41)						
ν_19_	a′	1437	(26)	1434	(18)	1443.2	(21)						
ν_20_	a′	1415	(18)	1410	(13)	1414.9	(26)						
ν_21_	a′	1338	(19)	1329	(11)	1336.0	(3)	1332	(13)	1321	(3)	(A)^f^	
ν_22_	a′	1306	(64)	1303	(7)	1306.0	(49)	1321	(7)	1309	(6)	1321.4	(35)
ν_23_	a′							1290	(6)	1290	(6)	1290.5?	(65)
ν_24_	a′	1282	(29)	1281	(22)	1283.9	(38)						
ν_25_	a′							1252	(13)	1248	(9)	1249.7	(24)
ν_27_	a′	1203	(17)	1198	(13)	1200.0	(10)						
ν_28_	a′	1165	(11)	1171	(1)	1161.6	(13)						
ν_29_	a′	1152	(10)	1158	(10)	1148.7	(15)						
ν_30_	a′	1137	(19)	1139	(4)	1139.8	(5)	1112	(4)	1115	(3)	1111.2	(12)
ν_31_	a′	1132	(7)	1130	(17)	1127.1	(13)	1036	(5)	1036	(4)	1033.3?	(18)
ν_33_	a′	1049	(6)	1047	(4)	1045.9	(10)						
ν_35_	a′	989	(18)	995	(7)	992.4	(21)	860	(24)	860	(20)	864.8	(65)
ν_45_	a″							2813	(12)	2753	(17)	(HCl)^f^	
ν_49_	a″							950	(4)	971	(2)	949.9	(18)
ν_50_	a″							928	(15)	937	(17)	929.7	(18)
ν_53_	a″	743	(110)	737	(34)	744.6	(100)						
ν_54_	a″	728	(2)	723	(162)			760	(62)	760	(49)	759.5	(100)
ν_55_	a″	714	(51)	705	(4)	709.4	(36)						
ν_56_	a″	605	(10)	611	(6)			724	(19)	724	(10)	720.3?	^e^
ν_57_	a″							655	(24)	652	(22)	651.1	(41)

a Modes with IR intensities less than 10 km mol^–1^ are not listed unless observed.

b Harmonic vibrational wavenumbers scaled according to 0.9510 *x* + 35.9 for wavenumbers >2000 cm^–1^ and 0.9778 *x* + 6.3 for wavenumbers <2000 cm^–1^.

c IR intensities in km mol^–1^.

d Percentage IR intensities relative to that of the most intense line in each species.

e Severe interference of other absorption lines prevents estimation of the observed IR intensity.

f Interference due to absorption of the parent or products, in which the interfering group number or HCl is indicated in parentheses.

**Table 7:** Comparison of Observed Vibrational Wavenumbers (in cm^–1^) and Relative IR Intensities of Lines in Groups E and F with Scaled Harmonic and Anharmonic Vibrational Wavenumbers and IR Intensities of 10-HC_13_H_9_N and 7-HC_13_H_9_N Predicted Using the B3LYP/6-311++G­(d,p) Method

		10-HC_13_H_9_N (group E)						7-HC_13_H_9_N (group F)					
mode^a^	sym	harmonic^b^		anharmonic		experiment		harmonic^b^		anharmonic		experiment	
ν_1_	a′	3075	(13)^c^	3090	(7)^c^	^d^		3091	(12)^c^	3112	(5)^c^	3083.3?	^e^
ν_2_	a′	3070	(15)	3084	(10)	^d^		3075	(13)	3108	(9)	3072.2?	^e^
ν_3_	a′	3066	(19)	3070	(7)	^d^		3070	(9)	3064	(9)	3065.1?	e
ν_5_	a′	3054	(18)	3049	(2)	^d^		3057	(32)	3035	(3)	3052.3?	^e^
ν_8_	a′	3001	(23)	2985	(19)	3011.1?	^e^	2995	(28)	2974	(22)	3009.3?	^e^
ν_9_	a′	2838	(24)	2837	(6)	2827.3?	(1)^f^	2842	(25)	2831	(4)	2832.3?	(13)^f^
ν_11_	a′	1579	(8)	1566	(2)	1578.2?	^e^	1567	(17)	1556	(1)	1560.0	(27)
ν_12_	a′	1562	(7)	1554	(3)	1564.9?	(3)	1562	(11)	1553	(9)		
ν_14_	a′	1509	(27)	1502	(19)	1511.3	(13)	1491	(21)	1483	(18)	1489.0	(33)
ν_22_	a′							1296	(19)	1290	(4)	1291.6	(B)^e^
ν_23_	a′							1281	(19)	1273	(29)	1275.9	(58)
ν_27_	a′	1167	(14)	1165	(7)	1151.9?	(6)						
ν_32_	a′							1031	(6)	1031	(3)	1028.5?	(7)
ν_34_	a′							930	(11)	929	(6)	922.8?	(7)
ν_35_	a′							879	(16)	890	(1)	868.8?	(13)
ν_45_	a″	2836	(12)	2784	(15)	(HCl)^d^		2841	(10)	2786	(14)	(HCl)^d^	
ν_51_	a″							899	(10)	905	(3)	897.5	(20)
ν_54_	a″	764	(14)	763	(4)	764.8	(6)	763	(41)	775	(5)	761.7	(93)
ν_55_	a″	755	(44)	754	(52)	754.8	(100)	754	(22)	753	(54)	752.3	(2)0
ν_56_	a″	664	(64)	663	(55)	661.4	(25)	662	(60)	665	(42)	663.6	(100)
ν_57_	a″	647	(5)	640	(7)	641.2	(3)						

a Modes with IR intensities less than 10 km mol^–1^ are not listed unless observed.

b Harmonic vibrational wavenumbers scaled according to 0.9510*x* + 35.9 for wavenumbers >2000 cm^–1^ and 0.9778*x* + 6.3 for wavenumbers <2000 cm^–1^.

c IR intensities in km mol^–1^.

d Interference due to absorption of the parent or products, in which the interfering group number or HCl is indicated in parentheses.

e Severe interference of parent or product absorption lines prevents estimation of the observed IR intensities; in the latter case, the group number of the interfering line is indicated in parentheses.

f Percentage IR intensities relative to that of the most intense line in each species.

Simulated spectra of alternative isomers of phenanthridinyl radicals failed to reproduce the observed wavenumbers and relative intensities of lines in group F satisfactorily (Figures 5 and S9, S10), thereby supporting the assignment to 7-HC_13_H_9_N. For the 17 lines in group F listed in Table 7, the mean absolute deviation between observed and scaled harmonic vibrational wavenumbers is 5.2 ± 3.7 cm^–1^ and that for the anharmonic counterpart is 11 ± 12 cm^–1^, indicating satisfactory agreement between experimental data and theoretical predictions for 7-HC_13_H_9_N.

### 5.3 Assignments of Lines in Groups B and E to 9-HC_13_H_9_N and 10-HC_13_H_9_N Radicals

The difference spectra after irradiation at 360 nm (lower trace) and 315 nm (upper trace) in region 970–670 cm^–1^ are presented in Figure 5e to compare to predicted IR stick spectra of 9- and 10-HC_13_H_9_N, shown in Figure 5df, respectively. Lines in group B are indicated with blue dashed lines and labels, while those in group E are indicated with violet. Similar comparisons for other spectral regions are shown in Figure S9. Comparisons of lines in group B with predicted IR stick spectra of other isomers of HC_13_H_9_N are shown in Figure S10.

As shown in these figures, the spectral pattern of observed lines in group B agrees satisfactorily with the predicted IR stick spectrum of the 9-phenanthridinyl radical, 9-HC_13_H_9_N, but agree poorly with IR stick spectra of other monohydrogenated radicals. Table 6 compares the observed wavenumber and relative IR intensities of lines in group B with both scaled harmonic and anharmonic vibrational wavenumbers and IR intensities predicted for 9-HC_13_H_9_N. Intense lines observed at 2799.1, 1476.8, 1321.4, 1290.5, 1249.7, 864.8, 759.5, and 651.1 cm^–1^ correlate well with those predicted near 2824 (ν_9_, symmetric CH_2_ stretch), 1478 (ν_14_), 1321 (ν_22_), 1290 (ν_23_), 1252 (ν_25_), 860 (ν_35_), 760 (ν_54_), and 655 (ν_57_) cm^–1^. Additionally, five weaker lines with intensity <20% of the most intense feature at 759.5 cm^–1^ were also observed, while six lines are marked with question marks due to interference from parent molecule or other products.

The simulated spectra for other isomers of phenanthridinyl radicals failed to reproduce satisfactorily both the wavenumbers and the relative intensities of lines in group B (Figures 5 and S9, S10). Consequently, these lines of group B are assigned to 9-HC_13_H_9_N. As listed in Table 6, among the 15 lines in group B, all listed modes with harmonic IR intensity >10 km mol^–1^ were observed except for four that were interfered by absorption from the parent, HCl, and a feature of group A at 1306.0 cm^–1^. The mean absolute deviation between observed and scaled harmonic vibrational wavenumbers is 3.6 ± 6.2 cm^–1^, while that for anharmonic vibrational wavenumbers is 6.5 ± 5.9 cm^–1^.

Similarly, lines in group E are assigned to the 10-phenanthridinyl radical, 10-HC_13_H_9_N. Three intense lines observed at 1511.3, 754.8, and 661.4 cm^–1^ agree satisfactorily with three most intense lines predicted at 1509 (ν_14_), 755 (ν_55_), and 664 (ν_56_) cm^–1^, respectively. Additionally, two weak lines at 764.8 and 641.2 cm^–1^ were located near the predicted scaled harmonic vibrational wavenumber predicted at 764 (ν_54_) and 647 (ν_57_) cm^–1^, respectively. Five additional features were marked with ? marks due to spectral interference from other species. The simulated spectra of other isomers of phenanthridinyl radical failed to reproduce the wavenumbers and relative intensities of lines in group E (Figures 5 and S9, S10). Table 7 presents a comparison of observed wavenumber and relative IR intensities for 10 lines in group E, with both scaled harmonic and anharmonic vibrational wavenumbers and relative IR intensities predicted for 10-HC_13_H_9_N. All vibrational modes with calculated IR intensity above 10 km mol^–1^ were observed, except for the five CH-stretching modes that were interfered with by parent absorptions. The average absolute deviation between observed and scaled harmonic (anharmonic) vibrational wavenumbers is 5.1 ± 5.1 (8.7 ± 7.9) cm^–1^.

### 5.4 Assignments of Lines in Groups C and D to 1-HC_13_H_9_N and 2-HC_13_H_9_N Radicals

The difference spectra after irradiation at 315 nm (lower trace) and 380 nm (upper trace) in region 970–670 cm^–1^ are presented in Figure 5g to compare with the predicted IR stick spectra of 1- and 2-HC_13_H_9_N, shown in Figure 5h,i, respectively. Lines in group C are indicated with green dashed lines and labels, and those in group E are indicated with orange. Similar comparisons in other spectral regions are shown in Figure S9. Comparisons with predicted IR stick spectra of alternative isomers of HC_13_H_9_N are depicted in Figure S10.

As illustrated, the observed spectral pattern of group C aligns well with the predicted IR stick spectrum of the 1-phenanthridinyl radical, 1-HC_13_H_9_N, but disagrees with those of other monohydrogenated radicals. Table 8 compares the observed wavenumber and relative IR intensities of lines in group B with both scaled harmonic and anharmonic vibrational wavenumbers and relative IR intensities predicted for 1-HC_13_H_9_N. Major lines observed at 1558.9, 1502.7, 1292.7, 1232.0, 677.1, and 654.9 cm^–1^ correspond closely with predicted transitions near 1558 (ν_12_), 1504 (ν_13_), 1292 (ν_23_), 1235 (ν_25_), 682 (ν_56_), and 661 (ν_57_) cm^–1^. Six weaker lines with intensity <10% of the most intense line at 677.1 cm^–1^ were also observed; four additional lines are marked with ? due to interference from absorption of parent or other products. The simulated spectra of other isomers of phenanthridinyl radicals failed to adequately reproduce the wavenumbers and relative intensities of lines in group C (Figures 5 and S9, S10). We hence assigned lines in group C to 1-HC_13_H_9_N. Of the 16 lines listed in Table 8 for group C, all vibrational modes with harmonic IR intensity >10 km mol^–1^ have been observed, except in the CH-stretching region, in which interference by the parent absorption is severe and modes ν_54_ (∼758 cm^–1^) and ν_55_ (∼749 cm^–1^) due to interference from lines of group B at 759.5 cm^–1^ and of the parent at 748.7 cm^–1^, respectively. The mean absolute deviation between observed and scaled harmonic vibrational wavenumbers is 4.5 ± 5.6 cm^–1^, and the mean absolute deviation is 9.5 ± 9.1 cm^–1^ for anharmonic computations.

**Table 8:** Comparison of Observed Vibrational Wavenumbers (in cm^–1^) and Relative IR Intensities of Lines in Groups C and D with Scaled Harmonic and Anharmonic Vibrational Wavenumbers and IR Intensities of 1-HC_13_H_9_N and 2-HC_13_H_9_N Predicted Using the B3LYP/6-311++G­(d,p) Method

		1-HC_13_H_9_N (group C)						2-HC_13_H_9_N (group D)					
mode^a^	sym	harmonic^b^		anharmonic		experiment		harmonic^b^		anharmonic		experiment	
ν_1_	a′							3075	(17)^c^	3085	(9)^c^	^d^	
ν_2_	a′	3074	(11)	3075	(16)	^d^		3065	(23)	3078	(30)	3070.7?	^e^
ν_3_	a′	3066	(19)	3054	(0)	^d^		3065	(12)	3065	(1)	^d^	
ν_4_	a′	3056	(25)	3046	(32)	^d^							
ν_5_	a′							3049	(12)	3047	(8)	^d^	
ν_8_	a′	2999	(25)	2991	(11)	^d^		3016	(15)	3002	(23)	^d^	
ν_9_	a′	2831	(27)	2771	(9)	2807.6?	(7)	2823	(55)	2752	(20)	2823.5?	(7)^f^
ν_10_	a′	1616	(29)	1607	(8)	1617.5?	^e^						
ν_11_	a′	1584	(27)	1575	(4)	1578.4?	^e^						
ν_12_	a′	1558	(18)	1550	(3)	1558.9?	(33)						
ν_14_	a′	1504	(16)	1496	(9)	1502.7	(40)						
ν_16_	a′	1421	(9)	1421	(5)	1427.1	(7)	1430	(10)	1429	(2)	1431.4	(7)
ν_17_	a′	1415	(8)	1408	(4)	1418.3	(7)						
ν_22_	a′							1303	(21)	1297	(4)	1297.5?	(15)
ν_23_	a′	1292	(36)	1279	(34)	1292.7	(20)						
ν_25_	a′	1235	(24)	1231	(19)	1232.0	(33)						
ν_26_	a′							1214	(7)	1211	(3)	1212.7?	(11)
ν_31_	a′							1037	(4)	1037	(3)	1033.1	(7)
ν_32_	a′	1030	(8)	1030	(7)	1027.1?	(13)						
ν_34_	a′	936	(15)	942	(49)	933.7	^e^	902	(15)	910	(8)	907.3	(26)
ν_35_	a′	865	(7)	877	(4)	869.6	(13)	867	(10)	867	(9)	(B)^d^	
ν_36_	a′							810	(7)	839	(0)	809.1?	(7)
ν_45_	a″	2828	(15)	2770	(19)	(HCl)^d^		2812	(15)	2746	(38)	(HCl)^d^	
ν_48_	a′	957	(6)	977	(5)	956.3	(7)						
ν_50_	a″							919	(12)	928	(1)	(C)^d^	
ν_51_	a″	923	(7)	918	(2)	918.9	(7)	874	(12)	902	(14)	872.4	(11)
ν_52_	a″							848	(8)	866	(17)	851.0?	(15)
ν_53_	a″							793	(10)	810	(4)	802.4	(11)
ν_54_	a″	758	(15)	783	(2)	(B)^d^							
ν_55_	a″	749	(34)	743	(39)	^d^		739	(60)	747	(23)	737.8	(100)
ν_56_	a″	682	(38)	687	(6)	677.1	(100)						
ν_57_	a″	661	(35)	650	(58)	654.9?	(93)	659	(18)	661	(10)	659.8	(15)

a Modes with IR intensities less than 10 km mol^–1^ are not listed unless observed.

b Harmonic vibrational wavenumbers scaled according to 0.9510*x* + 35.9 for wavenumbers >2000 cm^–1^ and 0.9778*x* + 6.3 for wavenumbers <2000 cm^–1^.

c IR intensities in km mol^–1^.

d Interference due to absorption of the parent or products, in which the interfering group number or HCl is indicated in parentheses.

e Severe interference of other absorption lines prevents estimation of the observed IR intensities.

f Percentage IR intensities relative to that of the most intense line in each species.

The observed spectral features in group D show good correspondence with the predicted IR stick spectrum of 2-phenanthridinyl radical, 2-HC_13_H_9_N, but deviate from those of other monohydrogenated radicals. Table 8 summarizes the observed and predicted wavenumbers and relative IR intensities for 2-HC_13_H_9_N. The most intense lines observed at 737.8 cm^–1^ were predicted at 739 (ν_55_) cm^–1^. Four additional medium-intensity lines were observed at 907.3, 872.4, 802.4, and 659.8 cm^–1^, consistent with the scaled harmonic vibrational wavenumber predicted near 902 (ν_34_), 874 (ν_51_), 793 (ν_53_), and 659 (ν_57_) cm^–1^. Six lines were indicated with ? marks due to interference from the parent or other species. Outside the CH-stretching region, only two vibrational modes of 2-HC_13_H_9_N with predicted IR intensities greater than 10 km mol^–1^, 867 (ν_35_) and 919 cm^–1^ (ν_50_), remain unobserved due to a spectral overlap; the former might be obscured by a line of group B at 864.5 cm^–1^ and the latter by a line of group C at 918.9 cm^–1^. In the CH-stretching region, a line observed at 2823.5 cm^–1^ may be tentatively assigned to the symmetric CH_2_ stretch, predicted at 2823 cm^–1^; however, the anharmonic vibrational calculation predicts a much smaller value of 2752 cm^–1^. Taken together, these 13 lines in group D are assigned to 2-HC_13_H_9_N. The mean absolute deviation between observed and scaled harmonic (anharmonic) vibrational wavenumbers is 3.1 ± 2.7 (14 ± 20) cm^–1^, indicating reasonable agreement within computational uncertainties.

### 5.5 Tentative Assignments of Lines in Groups G and H to 3-HC_13_H_9_N and 6-HC_13_H_9_N Radicals

In addition to the features assigned to groups A–F, ten lines in group G and eight in group H were observed. Difference spectra after irradiation at 425 nm (lower trace) and 315 nm (upper trace) are shown in Figure S10d to compare with predicted IR stick spectra for various isomers, 3-, 4-, 6-, 8-, 4a-, 6a-, 10a-, and 10b-HC_13_H_9_N, shown in Figure S10e–l, respectively. Lines tentatively attributed to group G are indicated with olive dashed lines and labels, while those in group H appear in red. Among them, only the most intense lines at 732.3 (group G) and 757.3 (group H) cm^–1^ were unambiguously assigned. The remaining lines could not be definitively identified and indicated with ? marks because of interference with absorption lines of other species; consequently, their photolytic behaviors are not consistently observed across different irradiation wavelengths.

The most intense feature at 732.3 cm^–1^ in group G corresponds to the ν_55_ of 3-HC_13_H_9_N predicted near 732 cm^–1^ (ν_55_ mode); interactions between ν_55_ and ν_56_ induce intensity variation and wavenumber shifts, as contrasted in harmonic and anharmonic vibrational analyses. However, we did not observe such an effect. Table 9 presents a comparison of the observed wavenumber and relative IR intensities of group G with both scaled harmonic and anharmonic values predicted for 3-HC_13_H_9_N. While the ten lines in group G are tentatively attributed to this species, they align well with predicted lines of 3-HC_13_H_9_N with IR intensities greater than 10 km mol^–1^ in region 600–1700 cm^–1^, except four lines predicted near 1600, 1586, 1500, and 1038 cm^–1^ (ν_11_, ν_12_, ν_14_, and ν_31_, respectively), which are interfered with by the parent absorption, and one line predicted near 872 cm^–1^, which is interfered with by a line of group D at 872.4 cm^–1^. Likewise, the CH-stretching region remains unresolved due to severe interference from the parent and products. The average absolute deviation between tentatively observed and scaled harmonic (anharmonic) vibrational wavenumbers is 5.0 ± 7.3 (7.0 ± 5.6) cm^–1^.

**Table 9:** Comparison of Observed Vibrational Wavenumbers (in cm^–1^) and Relative IR Intensities of Lines in Groups G and H with Scaled Harmonic and Anharmonic Vibrational Wavenumbers and IR Intensities of 3-HC_13_H_9_N and 6-HC_13_H_9_N Predicted Using the B3LYP/6-311++G­(d,p) Method

		3-HC_13_H_9_N (group G)						6-HC_13_H_9_N (group H)					
mode^a^	sym	harmonic^b^		anharmonic		experiment		harmonic^b^		anharmonic		experiment	
ν_1_	a′	3079	(18)^c^	3088	(12)^c^	^d^		3080	(18^c^	3110	(11)^c^	3083.3?	^e^
ν_2_	a′	3068	(24)	3064	(9)	^d^		3076	(17)	3085	(9)	3072.2?	^e^
ν_3_	a′							3069	(10)	3079	(9)	3065.1?	^e^
ν_5_	a′	3049	(14)	3038	(5)	^d^		3058	(19)	3043	(7)	^d^	
ν_8_	a′	2991	(26)	2996	(3)	^d^	^e^						
ν_9_	a′	2819	(60)	2811	(25)	2794.7?	(22)^f^	2842	(25)	2831	(4)	(HCl)^d^	^e^
ν_11_	a′	1600	(23)	1591	(7)	^d^							
ν_12_	a′	1586	(27)	1580	(21)	^d^		1575	(11)	1566	(4)	1568.4?	(20)^f^
ν_13_	a′	1539	(15)	1528	(8)	1538.8?	(22)						
ν_14_	a′	1500	(16)	1493	(11)	^d^							
ν_16_	a′							1451	(17)	1447	(9)	^d^	
ν_17_	a′							1417	(10)	1403	(2)	(A&C)^d^	^e^
ν_18_	a′							1404	(9)	1398	(3)	1399.4?	(80)
ν_19_	a′							1358	(11)	1348	(8)^d^	^d^	
ν_20_	a′	1360	(8)	1358	(3)	1350.7?	(11)						
ν_22_	a′	1334	(12)	1319	(4)	1331.0?	(11)						
ν_25_	a′	1228	(11)	1227	(6)	1224.7?	(6)						
ν_26_	a′	1221	(16)	1219	(0)	1218.6?	(22)						
ν_31_	a′	1038	(12)	1039	(3)	^d^							
ν_34_	a′	898	(20)	897	(13)	899.3?	(11)						
ν_35_	a′	872	(14)	896	(1)	(D)^d^							
ν_45_	a″	2806	(14)	2743	(19)	(HCl)^d^	^e^						
ν_54_	a″	755	(15)	759	(15)	753.4?	(E)^e^	771	(20)	776	(18)	770.7?	^e^
ν_55_	a″	732	(33)	745	(0)	732.3	(100)	756	(58)	767	(21)	757.3	(100)
ν_56_	a″	723	(64)	720	(87)	719.0?	^e^	721	(60)	722	(74)	719.8?	^e^

a Modes with IR intensities less than 10 km mol^–1^ are not listed unless observed.

b Harmonic vibrational wavenumbers scaled according to 0.9510*x* + 35.9 for wavenumbers >2000 cm^–1^ and 0.9778*x* + 6.3 for wavenumbers <2000 cm^–1^.

c IR intensities in km mol^–1^.

d Interference due to absorption of the parent or products, in which the interfering group number or HCl is indicated in parentheses.

e Severe interference of other absorption lines prevents estimation of the observed IR intensities.

f Percentage IR intensities relative to that of the most intense line in each species.

Eight lines were identified in group H, of which seven remain tentative due to interference with the absorption of other species. The most intense line at 757.3 cm^–1^ corresponds to a line predicted near 756 cm^–1^ for the ν_55_ mode; anharmonic computational analysis also revealed interactions between ν_55_ and ν_56,_ which induce intensity variations and wavenumber shifts, but we did not observe such an effect. Table 9 compares the observed wavenumber and relative IR intensities of group H with both scaled harmonic and anharmonic vibrational predictions for 6-HC_13_H_9_N. These eight lines in group G show a satisfactory correspondence, with transitions of 6-HC_13_H_9_N having predicted IR intensities greater than 10 km mol^–1^ in region 1700–600 cm^–1^, except three lines predicted at 1451, 1417, and 1385 cm^–1^ (ν_16_, ν_17_, and ν_19_, respectively) that are interfered with by the parent absorption. Similarly, the CH-stretching region could not be reliably analyzed due to severe interference from both the parent and products. The mean absolute deviation between tentatively assigned and scaled harmonic (anharmonic) vibrational wavenumbers is 3.1 ± 2.1 (9.3 ± 8.6) cm^–1^.

### 5.6 Representative Lines of Phenanthridinyl Radicals in Region 715–775 cm^–1^

Eight isomers of phenanthridinyl radicals were identified in a single C_13_H_9_N/Cl_2_/*p*-H_2_ matrix experiment. To illustrate the advantages of using *p*-H_2_ matrix isolation to identify all these isomers, we present in Figure 6 the spectra recorded in the representative region 670–775 cm^–1^, where the most intense lines of most isomers are predicted. The difference spectrum obtained after sequential UV/IR irradiation of a C_13_H_9_N/Cl_2_/*p*-H_2_ matrix is shown in Figure 6a, in which all positive features are attributed to various isomers of monohydrogenated phenanthridine and negative features are due to the loss of the parent, C_13_H_9_N. Figure 6b–d presents difference spectra recorded after irradiation at 445, 402, and 315 nm, respectively, highlighting the distinct photolytic behavior associated with each isomeric group. Spectral regions affected by absorption from the parent species are shaded gray. Each isomeric group is denoted using color-coded dashed lines and labels. Predicted stick spectra of ten isomers of HC_13_H_9_N are shown in Figure 6e–n, following the same color scheme; the prominent IR features are circled in thin orange (or gray for species not detected) to aid identification. These active modes primarily correspond to various types of out-of-plane CH-bending vibrations; for C_13_H_9_NH, the NH-bending motion is also included.

**Figure 6:**
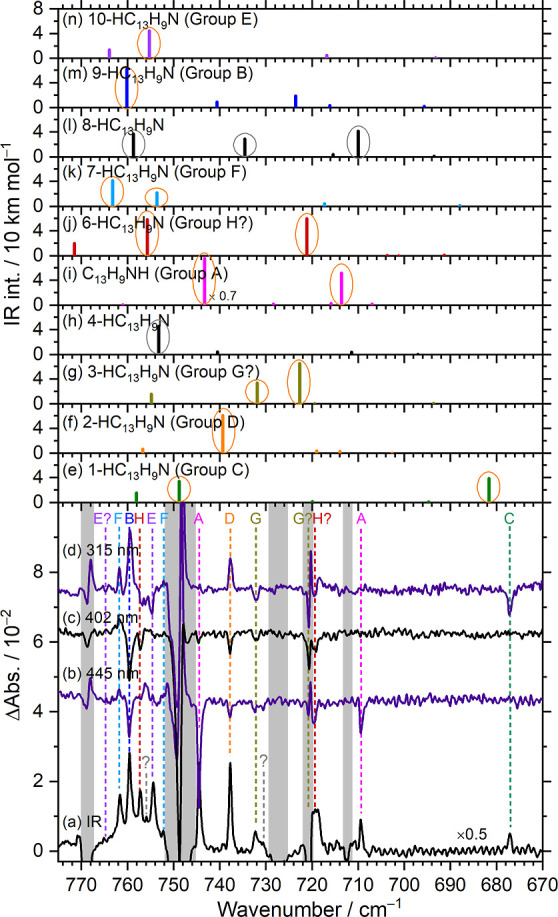
Comparison of the observed lines of groups A-H in region 670–775 cm^–1^ with the theoretically predicted stick spectra of ten isomers of hydrogenated phenanthridine (HC_13_H_9_N). (a) Difference spectrum of the matrix after irradiation with UV/IR light. Difference spectrum of the matrix after sequential secondary photolysis at 445 nm (b), 402 nm (c), and 315 nm (d). Lines of groups A–H are indicated with color-coded dashed lines and labels. IR stick spectra of 1-HC_13_H_9_N (e), 2-HC_13_H_9_N (f), 3-HC_13_H_9_N (g), 4-HC_13_H_9_N (h), C_13_H_9_NH (i), 6-HC_13_H_9_N (j), 7-HC_13_H_9_N (k), 8-HC_13_H_9_N (l), 9-HC_13_H_9_N (m), and 10-HC_13_H_9_N (n) simulated based on scaled harmonic wavenumbers and IR intensities predicted with the B3LYP/6-311++G­(d,p) method. Intense features are marked with orange or gray circles, the latter correspond to unobserved species. Spectral regions subjected to interference from the intense absorption of C_13_H_9_N are shaded gray.

Starting from the low wavenumber side, the first observed feature at 677.1 cm^–1^ (group C, green) is confidently assigned to 1-HC_13_H_9_N, as no other isomers are predicted to have absorption near this region. The subsequent feature at 709.4 cm^–1^ (group A, pink), accompanied by a more intense feature at 744.6 cm^–1^, matches well with those predicted for C_13_H_9_NH. Although 8-HC_13_H_9_N was predicted to exhibit a line near 710 cm^–1^ (black), its other intense features predicted near 735 and 759 cm^–1^ were not detected. The region near 720 cm^–1^ is interfered with by parent absorption. Nevertheless, two intense features at 719.8 cm^–1^ (group H, red) and 719.0 cm^–1^ (group G, olive) are tentatively assigned to 6-HC_13_H_9_N and 3-HC_13_H_9_N, respectively. A second feature for group G (3-HC_13_H_9_N) at 732.3 cm^–1^ is clearly identified. Likewise, the intense features at 737.8 cm^–1^ (group D, orange) and 744.6 cm^–1^ (group A, pink) are distinctly assigned to 2-C_13_H_9_NH and C_13_H_9_NH, respectively. In region 750–762 cm^–1^, four prominent features and two weak ones were observed. The prominent features at 754.8 (group E, purple), 757.3 (group H, red), 759.5 (group B, blue), and 761.7 (group F, light blue) cm^–1^ are unambiguously attributed to 10-, 6-, 9-, and 7-C_13_H_9_NH, respectively, consistent with predicted values near 755, 756, 760, and 763 cm^–1^. A weak feature at 752.3 cm^–1^ is assigned to 9-C_13_H_9_NH (group F, light blue, predicted near 754 cm^–1^). Another weak feature, observed at 756.0 cm^–1^, cannot be confidently attributed to known members of groups C, D, or G, despite predictions of weak lines near 758, 757, and 755 cm^–1^ for 1-, 2-, and 3-C_13_H_9_NH because the photolytic behavior of this line does not match that of other lines in groups C, D, and G. One possible assignment of this line would be to the unobserved 4-C_13_H_9_NH, predicted near 753 cm^–1^. However, due to the absence of other features to be associate with this line, no definitive assignment is made. Remarkably, the relative ordering of these five lines agrees well with theoretical predictions, even though the differences for each line are ∼2 cm^–1^. Although only one line from each of groups G and H was definitively identified, their respective assignments are considered reasonable.

### 5.7 Mechanism of Formation in *p*-H_2_

The formation of 5-phenanthridinium (C_13_H_9_NH^+^) is expected to proceed via a mechanism analogous to those observed in protonated PAH.^22,25^ During matrix deposition of *p*-H_2_ under electron bombardment, H_2_ molecules are ionized to produce H_2_^+^, which promptly react with another H_2_ molecule to generate H and H_3_^+^. Proton transfer from H_3_^+^ to phenanthridine occurs readily, owing to the significantly larger proton affinity of phenanthridine. The proton affinity is 977 kJ mol^–1^ for the formation of C_13_H_9_NH^+^ and >827 kJ mol^–1^ for the formation of H^+^C_13_H_9_N, much greater than that of H_2_, 422 kJ mol^–1^.^20^ The exclusive observation of C_13_H_9_NH^+^ in our experiments supports that C_13_H_9_NH^+^ has a much lower energy than other isomers, which have energies ≥150 kJ mol^–1^ higher than C_13_H_9_NH^+^. This result parallels our previous findings on the protonation of quinoline^27^ and isoquinoline,^29^ in which only the lowest-energy N-protonated PAHs were detected.

For H-addition to phenanthridine, the barriers for the hydrogenation on nonbridging carbon sites, 24–33 kJ mol^–1^, are much smaller than those on bridging carbon sites, 40–64 kJ mol^–1^, explaining the absence of 4a-, 6a-, 10a-, and 10b-HC_13_H_9_N. Among the ten nonbridging sites, eight monohydrogenated species were observed. The estimated mixing ratios for C_13_H_9_NH, 1-, 2-, 3-, 6-, 7-, 9-, and 10-HC_13_H_9_N are 2.7 ± 0.4, 2.3 ± 0.2, 2.3 ± 0.5, 1.6 ± 0.6, 1.4, 2.3 ± 0.3, 2.2 ± 0.1, and 1.9 ± 0.1 ppm, respectively. Due to uncertainties in the calculated IR intensities, no clear correlation was found between the observed mixing ratios and either the calculated barriers for formation or relative energies of phenanthridinyl radicals as computed using the CCSD­(T) method. To draw definitive conclusions regarding site-selective hydrogenation is hence challenging. The reason for the absence of 4- and 8-HC_13_H_9_N is unclear; likely interference from the parent and eight observed isomers of HC_13_H_9_N hampers the definitive identification of sufficient lines in a group.

### 5.8 Secondary Irradiation on C_13_H_9_NH^+^ and HC_13_H_9_N

Only one protonated phenanthridine, C_13_H_9_NH^+^, was observed. Vertical excitation wavelengths of C_13_H_9_NH^+^ were predicted at 405 and 362 nm, in line with the observation of the most prominent decrease of features in group A^+^ at 420 nm. The decrease in C_13_H_9_NH^+^ at 336 nm might be associated with absorption to the second excited states with vertical excitation near 362 nm. A similar decrease at 457 nm might indicate that the excitation to the first excited state is extended to this wavelength.

For HC_13_H_9_N, several isomers were involved. The vertical excitation spectra in the range 200–900 nm of the parent C_13_H_9_N and 14 isomers of HC_13_H_9_N, predicted using the TD-B3LYP/6-311++G­(d,p) method, are illustrated in Figure S11. Detailed excitation wavelengths and oscillator strengths are summarized in Table S11. The wavelengths for secondary irradiation were selected to exceed 315 nm to avoid photolysis of the parent molecule, whose UV absorption onset is predicted near 311 nm. Hydrogen transfer to a neighboring site (except the bridging-C sites) requires less than 226 kJ mol^–1^, which corresponds to irradiation of ∼529 nm. The secondary photoirradiation was employed mainly for the grouping of observed features; understanding the photolytic behavior of each isomer of HC_13_H_9_N is beyond the scope of this work.

Interpreting the observed photolytic behavior across all eight detected species at all wavelengths remains challenging due to substantial spectral overlap and the occurrence of hydrogen transfer at multiple sites for each wavelength. In addition, the large uncertainties in computed IR intensities hinder the reliability of absolute mixing ratio comparisons between species; only relative variations within one species are considered to be dependable. The decrease in mixing ratio depends on the original mixing ratio, so that their percentage decreases after each step, listed in parentheses in Table 5, are expected to offer insight into UV/vis absorption, although they might be convoluted by hydrogen transfer from adjacent sites.

For each species, the most pronounced percentage decrease is marked with an asterisk (*) in Table 5. The percentage decreases in mixing ratios following each irradiation step generally correlate with the predicted electronic absorption profiles. For instance, 9-HC_13_H_9_N exhibits its largest percentage decrease at 360 nm, consistent with a strong absorption band predicted near 376 nm, while smaller decreases at 402 and 380 nm align with a weaker absorption band predicted near 420 nm; the decrease at 445 nm corresponds to a weaker band near 467 nm. Similarly, for 2-HC_13_H_9_N, the most significant decrease occurs at 360 nm, in agreement with its strong absorption band predicted near 351 nm, while a smaller decrease occurs at 380 nm, corresponding to a weaker absorption band predicted near 365 nm. 1-, 10-, 3-, and 6-HC_13_H_9_N all show the largest percentage decrease at 315 nm, consistent with their predicted absorption bands near 324, 323, 328, and 349 nm, respectively. However, for 1-HC_13_H_9_N, the most intense vertical absorption band is near 380 nm, but its abundance increases by ∼35%, likely because 2-HC_13_H_9_N decreases significantly at this wavelength, transferring hydrogen to site 1. Notably, 3- and 6-HC_13_H_9_N also exhibited substantial percentage decreases at 402 nm, aligning with several predicted absorption bands in the ranges 369–438 and 372–406 nm, respectively. Although the most intense absorption band of C_13_H_9_NH is predicted near 345 nm, the observed percentage decrease at 360 nm was 50%, slightly smaller than the 63% decrease at 445 nm. This discrepancy may be attributed to the small mixing ratio of C_13_H_9_NH (0.22 ppm) prior to 360 nm irradiation, which results in large uncertainties (∼70%) in percentage decrease. In contrast, 7-HC_13_H_9_N possesses two intense bands and one medium band predicted near 289, 418, and 358 nm, respectively, yet it showed an increase in the mixing ratio at most wavelengths, with only slight decreases at 380 and 360 nm. This lack of a decrease in the mixing ratio for 7-HC_13_H_9_N might result from enhanced hydrogen transfer from 6-HC_13_H_9_N; 6-HC_13_H_9_N shows nearly no decrease or a significant increase at 380 and 360 nm, respectively, indicating the lack of hydrogen transfer from 6-HC_13_H_9_N to 7-HC_13_H_9_N at these two wavelengths.

At each irradiation wavelength, certain isomers decreased in abundance, while others increased, a trend generally explained by site-to-site hydrogen transfer. For instance, irradiation at 380 nm led to a significant decrease in 2-HC_13_H_9_N accompanied by a substantial increase in 1-HC_13_H_9_N. Similarly, 6-HC_13_H_9_N decreased at 410 nm, while 7-HC_13_H_9_N increased, suggesting that hydrogen transfer can occur between sites 6 and 7, even though they are separated by a bridging carbon (site 6a). A more complex pattern emerged upon irradiation at 315 nm: while the mixing ratios of 10-, 1-, 3-, and 6-HC_13_H_9_N decreased, those of 7-, 9-, and 2-HC_13_H_9_N increased. These changes can be rationalized by the following hydrogen transfers: 10 → 9, 3 → 2, 1 → 2, and 6 → 7. At 445 nm, 7-HC_13_H_9_N increased, while C_13_H_9_NH decreased, indicating a possible multistep hydrogen transfer of 5 → 6 → 7, supported by the intense absorption band of 6-HC_13_H_9_N predicted near 406 nm. In general, experimental observations support the idea that secondary irradiation induces hydrogen transfer to a neighboring site.

### 5.9 Implication to Astrochemistry

The UIR emission spectrum observed from the PDR^41^ is compared in Figure S12 with the stick IR spectra of phenanthridinium (C_13_H_9_NH^+^) and six phenanthridinyl (C_13_H_9_NH and 9-, 1-, 2-, 10-, 7-HC_13_H_9_N) radicals. Experimental spectra convoluted with a full width at half-maximum (fwhm) of 10 cm^–1^ are shown as dark lines. To account for unidentified bands due to spectral interference, light lines representing convoluted spectra based on scaled harmonic vibrational wavenumbers and IR intensities predicted using the B3LYP/6-311++G­(d,p) method are also presented. The UIR bands are UV-induced IR emission spectra, which are expected to be red-shifted by about 10–20 cm^–1^ from IR absorption bands of gaseous species observed at ambient temperature in laboratories because of anharmonicity.

In the vicinity of 6.2 μm, similar to other H^+^PANH such as protonated quinoline^27^ and isoquinoline,^29^ the most intense line of the H^+^PAH, 9-phenenthrenium cation (9-H^+^C_14_H_10_) in solid *p*-H_2_, was observed at 1611.5 cm^–1^ (∼6.21 μm).^42^ However, the most intense line predicted for the corresponding H^+^PANH, 5-phenenthridinium cation (C_13_H_9_NH^+^) at 1620 cm^–1^, was not observed due to spectral interference. The observed line at 1635.9 cm^–1^ (ν_11_, ∼ 6.11 μm) is blue-shifted from that of 9-H^+^C_14_H_10_. After consideration of the anharmonicity associated with the vibrationally excited emitting states produced upon UV irradiation in UIR bands,^14,43^ the C=C stretching bands of C_13_H_9_NH^+^ are arguably more consistent with the observed 6.2 μm UIR band than those of 9-H^+^C_14_H_10_. In contrast, isomers of HC_13_H_9_N radicals exhibit C=C stretching bands that absorb below 1618 cm^–1^ and a prominent band in the range 13.1–13.6 μm, suggesting that HPANH species are less likely contributors to the UIR bands.

Relatively intense lines of out-of-plane CH-bending modes were observed at 797.6 and 780.2 cm^–1^ (12.5 and 12.8 μm, respectively) in C_13_H_9_NH^+^, closely matching the 12.8 μm band of the UIR emission. In comparison, the most intense bands of various isomers of HC_13_H_9_N appear in the range 738–762 cm^–1^ (13.1–13.5 μm), slightly red-shifted relative to the 12.8 μm UIR band. Other prominent UIR bands near 7.7, 8.6, and 11.2 μm (∼1299, 1163, and 893 cm^–1^) are either absent or weak in the absorption spectra of C_13_H_9_NH^+^ and HC_13_H_9_N, supporting the expectation that phenanthridine is too small to withstand the intense UV radiation fields in interstellar environments, hence contributing insignificantly to the UIR bands.

## 6 Conclusion

Electron bombardment during matrix deposition of a mixture of phenanthridine (C_13_H_9_N) and *p*-H_2_ yielded C_13_H_9_NH^+^ and various isomers of HC_13_H_9_N. Spectral features of C_13_H_9_NH^+^ were identified based on diminished intensities after prolonged maintenance of the matrix in darkness and comparison with vibrational wavenumbers and IR intensities predicted at the B3LYP/6-311++G­(d,p) level of theory.

To enhance the hydrogenation reactions, a C_13_H_9_N/Cl_2_/*p*-H_2_ matrix was irradiated at 365 nm to generate Cl atoms, followed by IR-induced production of H atoms via Cl + H_2_ (*v* = 1). This enabled subsequent quantum-tunneling reactions of H atoms throughout the *p*-H_2_ matrix, leading to efficient reactions between H atoms and C_13_H_9_N to produce eight isomers of HC_13_H_9_N singly hydrogenated on nonring-sharing carbon atoms of phenanthridine. Observed IR lines were classified unambiguously into six groups and assigned to C_13_H_9_NH, 1-, 2-, 7-, 9-, and 10-HC_13_H_9_N based on their behavior upon secondary photolysis at 445, 425, 410, 402, 380, 360, and 315 nm and comparison with computations using the B3LYP/6-311++G­(d,p) method. Most lines with predicted IR intensities >10 km mol^–1^ were observed. In addition, some features were tentatively assigned to 3- and 6-HC_13_H_9_N, although interference from parent species and other isomers limited definitive identification of sufficient lines. The IR spectra of C_13_H_9_NH^+^ and isomers of HC_13_H_9_N reported here have been previously unobserved and offer valuable reference data for identifying related radicals in combustion and astrophysical environments. The unique characteristics of the H reactions in solid *p*-H_2_, using Cl atoms and IR light to induce hydrogenation reactions, include the high efficiency of the H-tunneling reaction in solid *p*-H_2_, absence of multiple hydrogenations, and simplified spectra with narrow lines, enabling identification of various isomers of HC_13_H_9_N.

The C=C stretching band of 5-phenenthridinium cation (C_13_H_9_NH^+^) at 1635.9 cm^–1^ provides improved correspondence to the UIR bands near 6.2 μm compared to H^+^PAH, after considering a possible red-shift in emission due to anharmonicity. Nonetheless, other IR features of C_13_H_9_NH^+^ and HC_13_H_9_N fail to match the UIR bands, rendering these molecules unlikely candidates as carriers.

## Supplementary Material



## Notes

Cartesian coordinates of optimized geometries of isomers of H^+^C_13_H_9_N and HC_13_H_9_N; comparison of observed vibrational wavenumbers of C_13_H_9_N with literature and computation values; scaled harmonic and anharmonic vibrational wavenumbers and IR intensities of various isomers of C_13_H_9_NH^+^ and HC_13_H_9_N; integrated lines and estimates of mixing ratios of each species in the C_13_H_9_N/Cl_2_/*p*-H_2_ matrix experiments; vertical excitation wavelengths and oscillator strengths of electronic excitation of various isomers of C_13_H_9_N (11 tables); geometries and relative energies of isomers of H^+^C_13_H_9_N and HC_13_H_9_N; potential-energy scheme for the formation and isomerization of various isomers of H^+^C_13_H_9_N and HC_13_H_9_N; comparison of experimental and predicted IR spectra of C_13_H_9_N; scaling curve for observed and calculated harmonic vibrational wavenumbers of C_13_H_9_N; spectra of a C_13_H_9_N/Cl_2_/*p*-H_2_ matrix before and after irradiation with UV/IR light and at various stages of the experiments; comparison of the observed lines in groups A–H with theoretically predicted stick spectra of isomers of HC_13_H_9_N; predicted UV spectra of C_13_H_9_N and various isomers of HC_13_H_9_N; and comparison of UIR bands with observed spectra of hydrogenated phenanthridine (12 figures) (PDF)
