# Supplementary material for: Infrared Spectra of Protonated Phenanthridine (C13H9NH+) and Isomers of Monohydrogenated Phenanthridine (C13H9NH and 1‑, 2‑, 7‑, 9‑, and 10-HC13H9N) Isolated in Solid Para-Hydrogen
Source: J Phys Chem A. 2025 Nov 15;129(47):10987–1002. doi: 10.1021/acs.jpca.5c06624 (PMC12670388; doi:10.1021/acs.jpca.5c06624)
Supplement: Supplementary file 1 [file jp5c06624_si_001.pdf]

Supporting Information

Infrared Spectra of Protonated Phenanthridine ($C_{13}H_9NH^+$) and Isomers of Mono-Hydrogenated Phenanthridine ($C_{13}H_9NH$ and 1-, 2-, 7-, 9-, and 10-$HC_{13}H_9N$) Isolated in Solid *Para*-Hydrogen

Man-Lin Yang,[a] Yi-Shan Chung,[a] and Yuan-Pern Lee*[ab]

[a] Department of Applied Chemistry and Institute of Molecular Science, National Yang Ming Chiao Tung Yang Ming University, Hsinchu 300093, Taiwan
[b] Center for Emergent Functional Matter Science, National Yang Ming Chiao Tung University, Hsinchu 300093, Taiwan

E-mail: yplee@nctu.edu.tw.

Table of Contents

Table S1. Cartesian coordinates of optimized geometries of isomers of protonated phenanthridine ($H^+C_{13}H_9N$) predicted with the B3LYP/6-311++G(d,p) method...........S1

Table S2. Comparison of observed vibrational wavenumbers of $C_{13}H_9N$ with literature values and scaled harmonic and anharmonic vibrational wavenumbers predicted with the B3LYP/6-311++G(d,p) method S5

Table S3. Scaled harmonic and anharmonic vibrational wavenumbers and IR intensities of 1-, 2-, 3-, 4-$H^+C_{13}H_9N$, and $C_{13}H_9NH^+$ calculated with the B3LYP/6-311++G(d,p) method...S7

Table S4. Scaled harmonic and anharmonic vibrational wavenumbers and IR intensities of 6-, 7-, 8-, 9-, and 10- $H^+C_{13}H_9N$ calculated with the B3LYP/6-311++G(d,p) method S9

Table S5. Scaled harmonic and anharmonic vibrational wavenumbers and IR intensities of 4a-, 6a-, 10a-, and 10b-$H^+C_{13}H_9N$ calculated with the B3LYP/6-311++G(d,p) method S11

Table S6. Cartesian coordinates of optimized geometries of isomers of hydrogenated phenanthridine ($HC_{13}H_9N$) predicted with the B3LYP/6-311++G(d,p) method S13

Table S7. Scaled harmonic and anharmonic vibrational wavenumbers and IR intensities of 1-, 2-, 3-, 4-$C_{13}H_9N$, and $C_{13}H_9NH$ calculated with the B3LYP/6-311++G(d,p) method S17

Table S8. Table S8. Scaled harmonic and anharmonic vibrational wavenumbers and IR intensities of 6-, 7-, 8-, 9-, and 10-$C_{13}H_9N$ calculated with the B3LYP/6-311++G(d,p) method...S19

Table S9. Scaled harmonic and anharmonic vibrational wavenumbers and IR intensities of 4a-, 6a-, 10a-, and 10b-$C_{13}H_9N$ calculated with the B3LYP/6-311++G(d,p) method S21

Table S10. Integrated lines and estimated mixing ratios of each species in the UV/IR-irradiated $C_{13}H_9N/Cl_2/p$-H_2 matrix experiments..S23

Table S11. Vertical excitation wavelengths and oscillator strengths of electronic excitation of $C_{13}H_9N$, 1-, 2-, 3-, 4-$HC_{13}H_9N$, $C_{13}H_9NH$, 6-, 7-, 8-, 9-, and 10-$HC_{13}H_9N$ predicted with the TD-B3LYP/6-311++G(d,p) method...S24

Figure S1. Geometries and relative energies of isomers of protonated phenanthridine ($H^+C_{13}H_9N$) S27
Figure S2. Potential-energy scheme for the formation and isomerization of various isomers of protonated phenanthridine ($H^+C_{13}H_9N$, indicated as *n*-H^+) S31
Figure S3. Geometries and relative energies of isomers of hydrogenated phenanthridine ($HC_{13}H_9N$) S32
Figure S4. Potential-energy scheme for the formation and isomerization of various isomers of hydrogenated phenanthridine ($HC_{13}H_9N$, indicated as *n*-HPh) S36
Figure S5. Comparison of experimental and predicted IR spectra of $C_{13}H_9N$ S37
Figure S6. Linear fit of observed and calculated harmonic vibrational wavenumbers of $C_{13}H_9N$ in two spectral ranges S38
Figure S7. Spectra of a $C_{13}H_9N/Cl_2/p$-H_2 matrix before and after irradiation with UV/IR light S39
Figure S8. Spectra of a $C_{13}H_9N/Cl_2/p$-H_2 matrix in regions 3500−3450, 3150−2780, and 1650−550 cm^{-1} at various stages of the experiment S41
Figure S9. Comparison of the observed lines in groups A−F in regions 3500−3450, 3150−2780, and 1650−550 cm^{-1} with theoretically predicted stick spectra of assigned isomers of hydrogenated phenanthridine ($HC_{13}H_9N$) S45
Figure S10. Comparison of the observed lines of groups A−H in regions 3500−3450, 3150−2780, and 1650−550 cm^{-1} with theoretically predicted stick spectra of unassigned isomers of hydrogenated phenanthridine ($HC_{13}H_9N$) S52
Figure S11. UV spectra of $C_{13}H_9N$ and various isomers of $HC_{13}H_9N$ predicted with the TD-B3LYP/6-311++G(d,p) method S59
Figure S12. Comparison of UIR bands with observed spectra of protonated and hydrogenated phenanthridine. S60

Table S1. Cartesian coordinates of optimized geometries of isomers of protonated phenanthridine ($H^+C_{13}H_9N$) predicted with the B3LYP/6-311++G(d,p) method

	x	*y*	*z*	*x*	*y*	*z*
	1-$H^+C_{13}H_9N$			2-$H^+C_{13}H_9N$		
C1	1.906338	-3.007568	0.000000	-2.022956	-2.985882	0.000000
C2	0.527017	-2.920997	0.000000	-0.644558	-2.922126	0.000000
C3	-0.098863	-1.662831	0.000000	-0.000638	-1.665788	0.000000
C4	0.694640	-0.472913	0.000000	-0.757770	-0.462264	0.000000
C5	2.108076	-0.594306	0.000000	-2.153920	-0.553920	0.000000
C6	2.696760	-1.839127	0.000000	-2.771976	-1.798309	0.000000
C7	-1.530623	-1.540236	0.000000	1.418976	-1.574594	0.000000
C8	0.000000	0.765499	0.000000	0.000000	0.789857	0.000000
C9	-1.415761	0.742279	0.000000	1.452992	0.711409	0.000000
C10	-2.129606	1.953647	0.000000	2.254421	1.901007	0.000000
C11	-1.512877	3.223377	0.000000	1.683041	3.125739	0.000000
C12	-0.153095	3.297420	0.000000	0.213993	3.277364	0.000000
C13	0.694241	2.087067	0.000000	-0.572346	2.033341	0.000000
H1	-3.212272	1.873432	0.000000	3.328459	1.762793	0.000000
H2	-2.131905	-2.446654	0.000000	1.998183	-2.496159	0.000000
H3	2.388186	-3.978015	0.000000	-2.528377	-3.943515	0.000000
H4	-0.080973	-3.818354	0.000000	-0.046460	-3.826291	0.000000
H5	2.735384	0.288457	0.000000	-2.773090	0.334077	0.000000
H6	3.776580	-1.925372	0.000000	-3.854412	-1.851370	0.000000
H7	-2.123517	4.117441	0.000000	2.289106	4.024561	0.000000
H8	0.351424	4.257854	0.000000	-0.103774	3.901122	0.855273
H9	1.379790	2.155672	0.860647	-1.651268	2.142822	0.000000
H10	1.379790	2.155672	-0.860647	-0.103774	3.901122	-0.855273
N	-2.168568	-0.409713	0.000000	2.121407	-0.457738	0.000000
	3-$H^+C_{13}H_9N$			4-$H^+C_{13}H_9N$		
C1	-2.021752	-2.946427	0.000000	2.034882	-2.972125	0.000000
C2	-0.636115	-2.900068	0.000000	0.658724	-2.915726	0.000000
C3	0.023767	-1.665517	0.000000	0.005619	-1.661341	0.000000
C4	-0.730537	-0.451886	0.000000	0.761187	-0.452576	0.000000
C5	-2.145467	-0.531145	0.000000	2.162811	-0.540801	0.000000
C6	-2.774860	-1.758891	0.000000	2.782356	-1.779901	0.000000
C7	1.467301	-1.595918	0.000000	-1.409848	-1.573974	0.000000
C8	0.000000	0.776687	0.000000	0.000000	0.784087	0.000000
C9	1.443929	0.695850	0.000000	-1.423363	0.703254	0.000000
C10	2.186171	1.852579	0.000000	-2.241126	1.947442	0.000000
C11	1.569288	3.182266	0.000000	-1.518524	3.228456	0.000000
C12	0.093447	3.207829	0.000000	-0.156731	3.266269	0.000000
C13	-0.632759	2.065649	0.000000	0.579866	2.058100	0.000000
H1	3.267632	1.763917	0.000000	-2.939212	1.911615	0.853925
H2	2.029026	-2.527827	0.000000	-1.992977	-2.492144	0.000000
H3	-2.530420	-3.903244	0.000000	2.544881	-3.927414	0.000000
H4	-0.056232	-3.815820	0.000000	0.065207	-3.822855	0.000000
H5	-2.748463	0.366557	0.000000	2.779158	0.349070	0.000000
H6	-3.856750	-1.809967	0.000000	3.864849	-1.831159	0.000000
H7	1.961724	3.763964	0.854390	-2.100957	4.143502	0.000000
H8	-0.399602	4.173255	0.000000	0.376277	4.208704	0.000000
H9	-1.712301	2.126553	0.000000	1.661486	2.139023	0.000000
H10	1.961724	3.763964	-0.854390	-2.939212	1.911615	-0.853925
N	2.147028	-0.498200	0.000000	-2.104947	-0.448136	0.000000

	x	*y*	*z*	*x*	*y*	*z*
		$C_{13}H_9NH^+$			$6\text{-}H^+C_{13}H_9N$	
C1	2.049244	2.930236	0.000000	-2.111096	2.856634	0.000000
C2	0.676157	2.894049	0.000000	-0.725098	2.847371	0.000000
C3	0.004402	1.644808	0.000000	-0.033458	1.634927	0.000000
C4	0.736676	0.414733	0.000000	-0.752364	0.406900	0.000000
C5	2.141513	0.494957	0.000000	-2.170298	0.443404	0.000000
C6	2.777309	1.722852	0.000000	-2.834475	1.649053	0.000000
C7	-1.399524	1.599880	0.000000	1.455684	1.612815	0.000000
C8	0.000000	-0.833795	0.000000	0.000000	-0.813797	0.000000
C9	-1.416377	-0.796261	0.000000	1.475193	-0.740689	0.000000
C10	-2.195252	-1.963056	0.000000	2.258748	-1.964117	0.000000
C11	-1.564971	-3.189389	0.000000	1.642783	-3.174234	0.000000
C12	-0.159934	-3.258850	0.000000	0.218917	-3.228755	0.000000
C13	0.604315	-2.109772	0.000000	-0.573396	-2.095185	0.000000
H1	-3.277834	-1.894555	0.000000	3.335729	-1.851192	0.000000
H2	-2.002777	2.499606	0.000000	1.859499	2.183139	0.854917
H3	2.573572	3.877546	0.000000	-2.643335	3.800553	0.000000
H4	0.096450	3.810039	0.000000	-0.176136	3.782659	0.000000
H5	2.740989	-0.404940	0.000000	-2.742686	-0.474081	0.000000
H6	3.860566	1.757442	0.000000	-3.917225	1.671103	0.000000
H7	-2.152969	-4.098695	0.000000	2.213576	-4.094194	0.000000
H8	0.327530	-4.226011	0.000000	-0.263694	-4.200293	0.000000
H9	1.682303	-2.195311	0.000000	-1.647842	-2.214360	0.000000
H10	-3.059194	0.454711	0.000000	1.859499	2.183139	-0.854917
N	-2.044283	0.445402	0.000000	2.145111	0.372509	0.000000
		$7\text{-}H^+C_{13}H_9N$			$8\text{-}H^+C_{13}H_9N$	
C1	-1.468936	3.255969	0.000000	1.607710	3.174384	0.000000
C2	-2.218157	1.989660	0.000000	2.205587	1.831424	0.000000
C3	-1.416882	0.734827	0.000000	1.444799	0.695567	0.000000
C4	0.000000	0.786730	0.000000	0.000000	0.783003	0.000000
C5	0.610287	2.054202	0.000000	-0.615740	2.082096	0.000000
C6	-0.104186	3.271207	0.000000	0.128525	3.211179	0.000000
C7	-2.046966	-0.524490	0.000000	2.066494	-0.616854	0.000000
C8	0.743155	-0.456052	0.000000	-0.723713	-0.436598	0.000000
C9	-0.030144	-1.663724	0.000000	0.023568	-1.667660	0.000000
C10	0.619420	-2.922666	0.000000	-0.659822	-2.889978	0.000000
C11	1.993460	-2.988835	0.000000	-2.044057	-2.915891	0.000000
C12	2.754114	-1.803357	0.000000	-2.789427	-1.719055	0.000000
C13	2.144565	-0.560251	0.000000	-2.145751	-0.504374	0.000000
H1	-0.000889	-3.810023	0.000000	-0.074086	-3.800343	0.000000
H2	-3.132320	-0.589318	0.000000	3.153510	-0.673753	0.000000
H3	-2.034431	4.181824	0.000000	2.005455	3.748816	0.855990
H4	-2.914488	1.994786	0.857242	3.289179	1.753093	0.000000
H5	1.692841	2.113597	0.000000	-1.693900	2.160056	0.000000
H6	0.442011	4.206188	0.000000	-0.351560	4.183300	0.000000
H7	2.495059	-3.948718	0.000000	-2.562955	-3.867457	0.000000
H8	3.836015	-1.863673	0.000000	-3.871558	-1.759262	0.000000
H9	2.770496	0.323328	0.000000	-2.735408	0.402379	0.000000
H10	-2.914488	1.994786	-0.857242	2.005455	3.748816	-0.855990
N	-1.388313	-1.663157	0.000000	1.406690	-1.722730	0.000000

	x	*y*	*z*	*x*	*y*	*z*
		$9\text{-}H^+C_{13}H_9N$			$10\text{-}H^+C_{13}H_9N$	
C1	1.670493	3.142448	0.000000	-1.530658	3.219967	0.000000
C2	2.245098	1.915825	0.000000	-2.142247	1.942793	0.000000
C3	1.443837	0.732397	0.000000	-1.419527	0.747964	0.000000
C4	0.000000	0.795977	0.000000	0.000000	0.771277	0.000000
C5	-0.582804	2.040660	0.000000	0.689958	2.098232	0.000000
C6	0.201500	3.283365	0.000000	-0.173267	3.301132	0.000000
C7	2.054318	-0.548037	0.000000	-2.098282	-0.524663	0.000000
C8	-0.746478	-0.458559	0.000000	0.685379	-0.460268	0.000000
C9	0.020048	-1.666842	0.000000	-0.106297	-1.664239	0.000000
C10	-0.629108	-2.923149	0.000000	0.538988	-2.913813	0.000000
C11	-2.005952	-2.987634	0.000000	1.917069	-2.984581	0.000000
C12	-2.759421	-1.801670	0.000000	2.704276	-1.809121	0.000000
C13	-2.142139	-0.557052	0.000000	2.105445	-0.574473	0.000000
H1	-0.010022	-3.811412	0.000000	-0.077898	-3.803410	0.000000
H2	3.138507	-0.620943	0.000000	-3.186351	-0.539872	0.000000
H3	2.271726	4.044205	0.000000	-2.145854	4.110760	0.000000
H4	3.323644	1.806811	0.000000	-3.227395	1.885105	0.000000
H5	-1.661820	2.146113	0.000000	1.373938	2.171577	0.860837
H6	-0.122328	3.908682	0.853206	0.322395	4.266311	0.000000
H7	-2.509430	-3.946404	0.000000	2.406264	-3.951618	0.000000
H8	-3.841690	-1.855798	0.000000	3.784409	-1.888526	0.000000
H9	-2.764166	0.329443	0.000000	2.723181	0.315251	0.000000
H10	-0.122328	3.908682	-0.853206	1.373938	2.171577	-0.860837
N	1.383079	-1.673680	0.000000	-1.481664	-1.662628	0.000000
		$4a\text{-}H^+C_{13}H_9N$			$6a\text{-}H^+C_{13}H_9N$	
C1	-3.529182	-0.291193	-0.181684	-3.529182	-0.291193	-0.181684
C2	-2.854317	0.860002	0.049188	-2.854317	0.860002	0.049188
C3	-1.399190	0.836440	0.332562	-1.399190	0.836440	0.332562
C4	-0.680040	-0.448766	0.124408	-0.680040	-0.448766	0.124408
C5	-1.450536	-1.609896	-0.063179	-1.450536	-1.609896	-0.063179
C6	-2.820387	-1.529221	-0.211666	-2.820387	-1.529221	-0.211666
C7	-0.586055	2.074887	0.016746	-0.586055	2.074887	0.016746
C8	0.747481	-0.405870	0.115252	0.747481	-0.405870	0.115252
C9	1.382035	0.867860	-0.058704	1.382035	0.867860	-0.058704
C10	2.770723	0.936819	-0.186783	2.770723	0.936819	-0.186783
C11	3.536665	-0.218685	-0.116753	3.536665	-0.218685	-0.116753
C12	2.929195	-1.471348	0.072493	2.929195	-1.471348	0.072493
C13	1.556770	-1.566324	0.177396	1.556770	-1.566324	0.177396
H1	3.223876	1.908912	-0.333812	3.223876	1.908912	-0.333812
H2	-1.113720	3.027337	0.009046	-1.113720	3.027337	0.009046
H3	-4.598482	-0.283891	-0.351995	-4.598482	-0.283891	-0.351995
H4	-3.365228	1.815594	0.100829	-3.365228	1.815594	0.100829
H5	-0.967873	-2.572517	-0.164559	-0.967873	-2.572517	-0.164559
H6	-3.380201	-2.441660	-0.387015	-3.380201	-2.441660	-0.387015
H7	4.614594	-0.153931	-0.207311	4.614594	-0.153931	-0.207311
H8	3.539893	-2.363209	0.138226	3.539893	-2.363209	0.138226
H9	1.103708	-2.536011	0.339076	1.103708	-2.536011	0.339076
H10	-1.403540	0.914898	1.453865	-1.403540	0.914898	1.453865
N	0.675427	2.068036	-0.144574	0.675427	2.068036	-0.144574

	x	*y*	*z*	*x*	*y*	*z*
	10a-$H^+C_{13}H_9N$			10b-$H^+C_{13}H_9N$		
C1	-3.516373	-0.225412	-0.210649	3.548417	-0.238935	-0.212546
C2	-2.788349	0.946226	-0.095541	2.798510	0.920871	-0.139663
C3	-1.400846	0.898929	0.076514	1.400536	0.844226	0.027834
C4	-0.733503	-0.406231	0.317751	0.761339	-0.414931	0.131212
C5	-1.559218	-1.611570	0.073115	1.527917	-1.571336	0.070920
C6	-2.898610	-1.504286	-0.142053	2.909530	-1.479968	-0.112572
C7	-0.587378	2.069743	0.059929	0.604997	2.033161	0.078259
C8	0.757630	-0.414275	0.101656	-0.729352	-0.395188	0.392787
C9	1.398930	0.854840	0.020370	-1.389136	0.914023	0.089123
C10	2.803464	0.924288	-0.110490	-2.774503	0.947403	-0.150026
C11	3.553053	-0.234258	-0.162826	-3.484704	-0.225538	-0.283598
C12	2.914089	-1.478969	-0.082890	-2.871206	-1.508777	-0.153682
C13	1.529377	-1.569279	0.060892	-1.550977	-1.608244	0.133523
H1	3.257314	1.905987	-0.164022	-3.242548	1.914671	-0.283743
H2	-1.068304	3.044134	0.023813	1.113462	2.995532	0.084211
H3	-4.587185	-0.170482	-0.373471	4.621873	-0.190591	-0.345514
H4	-3.280360	1.907432	-0.191172	3.273206	1.893148	-0.209903
H5	-1.093122	-2.585379	0.153435	1.075737	-2.551478	0.149852
H6	-3.502383	-2.393812	-0.273609	3.494003	-2.390009	-0.181841
H7	4.629778	-0.184701	-0.266707	-4.544953	-0.177317	-0.507677
H8	3.500256	-2.388612	-0.139510	-3.470683	-2.399935	-0.292529
H9	1.074795	-2.550075	0.122201	-1.089708	-2.578987	0.265050
H10	-0.781465	-0.402081	1.447626	-0.780611	-0.371480	1.515342
N	0.716728	2.045587	0.032393	-0.708284	2.079408	0.082475

Table S2. Comparison of observed vibrational wavenumbers of $C_{13}H_9N$ with literature values and scaled harmonic and anharmonic vibrational wavenumbers predicted with the B3LYP/6-311++G(d,p) method

mode	sym.	calculations harmonic / cm^{-1}	calculations scaled[a] / cm^{-1}	calculations anharmonic / cm^{-1}	experiment Ar / cm^{-1}	experiment p-H_2 / cm^{-1}
1	A'	3200	3079 (16)[b]	3119 (14)[b]	3114.8	3091.7 (1)[c]
2	A'	3196	3075 (18)	3079 (71)	3078.7	3082.8 (7)
3	A'	3190	3069 (15)	3065 (15)	3069.0	3071.1 (6)
4	A'	3186	3065 (6)	3058 (1)	3120－3000	3058.9 (1)
5	A'	3178	3058 (13)	3054 (10)	3044.5	3047.2 (5)
6	A'	3170	3051 (10)	3035 (5)	3034.3	3031.1 (3)
7	A'	3166	3046 (0)	3031 (1)		
8	A'	3162	3043 (3)	3023 (1)		
9	A'	3113	2997 (22)	2949 (14)	2966.3	2964.8 (3)
10	A'	1655	1625 (12)	1616 (7)	1623.8	1624.7 (3)
11	A'	1646	1616 (7)	1606 (3)	1615.0	1616.4 (2)
12	A'	1629	1600 (24)	1594 (18)	1582.0	1595.2 (18)
13	A'	1608	1578 (7)	1570 (0)		1580.0 (4)
14	A'	1562	1533 (8)	1527 (1)	1530.1	1530.4 (4)
15	A'	1519	1492 (13)	1487 (7)	1492.7	1492.7 (10)
16	A'	1488	1462 (8)	1457 (3)	1463.2	1462.2 (6)
17	A'	1478	1451 (8)	1448 (2)	1449.3	1449.4 (4)
18	A'	1435	1410 (2)	1406 (1)	1418.2	1416.8 (1)
19	A'	1422	1397 (1)	1396 (1)	1397.3	1397.1 (1)
20	A'	1377	1353 (3)	1343 (1)		
21	A'	1363	1339 (2)	1334 (2)	1343.5	1343.3 (2)
22	A'	1318	1295 (2)	1293 (2)	1295.9	1295.2 (2)
23	A'	1304	1282 (0)	1281 (0)		1280.3 (0)
24	A'	1263	1241 (17)[b]	1239 (13)	1240.8	1239.9 (19)
25	A'	1249	1228 (1)	1229 (1)		1228.6 (1)
26	A'	1216	1196 (2)	1199 (1)	1194.7	1192.7 (2)
27	A'	1188	1168 (0)	1173 (0)		
28	A'	1170	1151 (2)	1151 (1)	1145.3	1144.9 (2)
29	A'	1157	1138 (4)	1140 (3)	1137.4	1136.4 (2)
30	A'	1122	1103 (1)	1104 (0)	1101.3	1100.5 (0)
31	A'	1056	1039 (6)	1041 (2)	1038.5	1038.3 (4)
32	A'	1055	1037 (3)	1038 (4)	1035.0	1035.5 (0)

33	A'	1019	1003 (2)	1021 (0)	1005.2	1005.4 (2)
34	A'	900	886 (12)	928 (8)	892.7	889.7 (12)
35	A'	844	831 (0)	833 (0)		834.6 (0)
36	A'	740	730 (6)	729 (4)	727.2	727.5 (4)
37	A'	723	713 (1)	714 (1)		
38	A'	629	622 (6)	624 (5)	615.4	615.9 (6)
39	A'	558	552 (0)	553 (0)		
40	A'	511	506 (1)	508 (1)		
41	A'	454	450 (2)	448 (2)		
42	A'	419	416 (0)	415 (1)		
43	A'	242	243 (1)	239 (1)		
44	A"	997	981 (0)	1013 (0)		
45	A"	994	979 (0)	1005 (2)		
46	A"	975	960 (7)	984 (0)	958.1	960.2 (7)
47	A"	965	950 (0)	978 (4)		951.2 (0)
48	A"	944	929 (7)	944 (2)	930.7	932.3 (6)
49	A"	878	865 (1)	888 (11)		874.7 (1)
50	A"	871	858 (0)	867 (0)		
51	A"	797	786 (2)	810 (0)	793.0	793.7 (2)
52	A"	780	769 (11)	771 (9)	767.2	768.1 (12)
53	A"	760	749 (110)	753 (95)	747.3	748.7 (100)
54	A"	733	723 (13)	718 (17)	720.6	720.5 (11)
55	A"	613	606 (1)	605 (1)		601.1 (1)
56	A"	548	542 (1)	550 (1)		
57	A"	504	499 (2)	505 (2)		
58	A"	436	433 (5)	434 (8)		
59	A"	413	410 (1)	414 (0)		
60	A"	246	247 (1)	243 (1)		
61	A"	227	229 (0)	225 (0)		
62	A"	101	105 (1)	102 (1)		
63	A"	96	100 (1)	95 (1)		
Reference			This work		Bernstein[d]	This work

[a]Harmonic vibrational wavenumbers scaled according to 0.9510 x + 35.9 for wavenumbers > 2000 cm^{-1} and 0.9778 x + 6.3 for wavenumbers < 2000 cm^{-1}. [b]IR intensities in km mol^{-1} are listed in parentheses. [c]Percentage IR intensities relative to that of the most intense line at 748.7 cm^{-1} are listed in parentheses. [d]Bernstein, M. P.; Mattioda, A. L.; Sandford, S. A.; Hudgins, D. M. *Astrophys. J.* **2005**, *626*, 909–918.

Table S3. Scaled harmonic and anharmonic vibrational wavenumbers and IR intensities of 1-, 2-, 3-, 4-$H^+C_{13}H_9N$, and $C_{13}H_9NH^+$ calculated with the B3LYP/6-311++G(d,p) method

ν_i	1-$H^+C_{13}H_9N$		2-$H^+C_{13}H_9N$		3-$H^+C_{13}H_9N$		4-$H^+C_{13}H_9N$		$C_{13}H_9NH^+$	
	scaled[a]	anharmonic	scaled[a]	anharmonic	scaled[a]	anharmonic	scaled[a]	anharmonic	scaled[a]	anharmonic
1	3087 (2)[b]	3084 (1)[b]	3088 (2)[b]	3082 (1)[b]	3100 (0)[b]	3108 (0)[b]	3086 (0)[b]	3079 (0)[b]	3420 (149)[b]	3395 (127)[b]
2	3085 (0)	3084 (2)	3086 (0)	3075 (2)	3088 (0)	3124 (0)	3085 (0)	3096 (1)	3100 (0)	3104 (1)
3	3077 (1)	3081 (0)	3079 (1)	3088 (0)	3081 (0)	3085 (3)	3079 (2)	3104 (1)	3090 (0)	3119 (0)
4	3068 (3)	3072 (2)	3070 (1)	3045 (0)	3073 (0)	3067 (0)	3069 (0)	3068 (1)	3086 (5)	3046 (1)
5	3068 (0)	3074 (2)	3069 (0)	3063 (1)	3071 (6)	3072 (2)	3067 (2)	3055 (0)	3085 (1)	3081 (0)
6	3064 (3)	3060 (1)	3065 (0)	3052 (1)	3070 (1)	3059 (0)	3064 (0)	3046 (0)	3083 (0)	3086 (3)
7	3063 (0)	3035 (1)	3062 (0)	3035 (0)	3064 (0)	3038 (1)	3061 (2)	3029 (1)	3073 (0)	3057 (0)
8	3031 (3)	3031 (2)	3030 (2)	3020 (3)	3027 (3)	3026 (3)	3036 (1)	3012 (0)	3069 (0)	3052 (3)
9	2873 (16)	2846 (6)	2855 (44)	2826 (42)	2853 (64)	2825 (40)	2871 (56)	2840 (37)	3063 (0)	3044 (0)
10	1610 (35)	1601 (20)	1636 (84)	1627 (30)	1627 (60)	1618 (39)	1614 (64)	1609 (11)	3055 (1)	3031 (0)
11	1596 (53)	1589 (14)	1605 (51)	1599 (12)	1601 (71)	1594 (14)	1601 (23)	1591 (15)	1632 (55)	1622 (18)
12	1587 (7)	1582 (12)	1574 (102)	1563 (22)	1591 (238)	1582 (118)	1569 (57)	1561 (44)	1620 (93)	1614 (29)
13	1550 (22)	1542 (12)	1549 (41)	1539 (33)	1556 (52)	1546 (24)	1540 (129)	1532 (28)	1610 (14)	1608 (5)
14	1512 (40)	1507 (25)	1512 (166)	1504 (104)	1523 (27)	1515 (14)	1517 (139)	1508 (51)	1574 (11)	1565 (4)
15	1476 (251)	1472 (96)	1497 (97)	1491 (47)	1473 (88)	1468 (64)	1503 (134)	1499 (37)	1557 (26)	1549 (15)
16	1450 (33)	1445 (38)	1453 (95)	1451 (22)	1467 (80)	1461 (43)	1441 (7)	1440 (3)	1531 (14)	1524 (1)
17	1420 (250)	1419 (55)	1441 (80)	1440 (35)	1442 (11)	1440 (7)	1428 (19)	1421 (3)	1479 (25)	1477 (9)
18	1412 (52)	1407 (9)	1396 (366)	1395 (283)	1404 (28)	1400 (1)	1423 (10)	1416 (21)	1456 (14)	1455 (6)
19	1403 (99)	1400 (21)	1388 (94)	1380 (34)	1392 (16)	1384 (14)	1378 (13)	1376 (10)	1446 (18)	1444 (4)
20	1381 (26)	1376 (8)	1365 (32)	1348 (26)	1368 (102)	1362 (41)	1358 (97)	1354 (1)	1420 (13)	1410 (0)
21	1348 (93)	1338 (79)	1345 (50)	1333 (6)	1350 (57)	1340 (25)	1340 (506)	1332 (313)	1371 (46)	1363 (34)
22	1323 (42)	1306 (21)	1310 (161)	1289 (79)	1326 (59)	1322 (21)	1310 (21)	1307 (0)	1362 (45)	1352 (15)
23	1309 (0)	1303 (2)	1297 (26)	1300 (16)	1299 (143)	1279 (112)	1296 (171)	1276 (144)	1327 (6)	1327 (4)
24	1273 (2)	1270 (3)	1285 (8)	1285 (7)	1281 (12)	1283 (12)	1280 (52)	1278 (54)	1297 (0)	1299 (0)
25	1241 (32)	1239 (9)	1267 (19)	1265 (11)	1244 (24)	1242 (16)	1256 (29)	1255 (8)	1267 (8)	1265 (1)
26	1225 (4)	1224 (1)	1235 (36)	1232 (14)	1233 (12)	1232 (4)	1226 (31)	1228 (6)	1247 (21)	1246 (16)
27	1179 (5)	1187 (5)	1215 (189)	1215 (98)	1181 (3)	1189 (5)	1210 (86)	1210 (65)	1235 (7)	1236 (4)
28	1174 (6)	1179 (3)	1178 (25)	1185 (29)	1176 (43)	1174 (38)	1180 (16)	1185 (10)	1201 (10)	1200 (8)
29	1162 (0)	1159 (0)	1149 (26)	1154 (15)	1160 (0)	1162 (0)	1166 (9)	1168 (5)	1182 (2)	1189 (0)
30	1130 (1)	1129 (1)	1126 (4)	1127 (2)	1123 (2)	1123 (0)	1131 (2)	1133 (1)	1173 (9)	1177 (10)
31	1063 (13)	1064 (11)	1042 (0)	1045 (0)	1043 (4)	1046 (2)	1072 (4)	1073 (4)	1153 (14)	1160 (4)
32	1034 (1)	1037 (1)	1035 (7)	1033 (0)	1028 (20)	1027 (14)	1039 (1)	1041 (1)	1113 (2)	1112 (0)
33	996 (5)	998 (2)	992 (5)	995 (4)	995 (6)	997 (6)	995 (4)	996 (4)	1043 (0)	1040 (0)

34	953 (23)	957 (13)	915 (30)	915 (31)	924 (27)	925 (27)	950 (15)	955 (7)	1039 (1)	1039 (2)
35	864 (7)	865 (5)	876 (2)	880 (2)	872 (14)	874 (13)	884 (8)	881 (5)	1000 (1)	1002 (0)
36	824 (1)	823 (0)	819 (2)	820 (2)	810 (14)	810 (13)	826 (6)	826 (4)	886 (1)	887 (1)
37	724 (6)	726 (5)	723 (5)	724 (4)	719 (0)	720 (0)	714 (1)	715 (1)	829 (0)	832 (0)
38	692 (10)	694 (10)	699 (0)	700 (0)	697 (5)	698 (4)	698 (0)	699 (0)	729 (3)	730 (3)
39	600 (1)	601 (1)	601 (6)	603 (2)	602 (6)	603 (5)	605 (2)	606 (2)	710 (1)	711 (1)
40	537 (13)	538 (11)	544 (3)	542 (2)	540 (7)	539 (6)	541 (10)	539 (10)	620 (4)	622 (3)
41	497 (0)	498 (0)	501 (5)	501 (4)	496 (2)	497 (2)	497 (5)	496 (4)	551 (1)	552 (1)
42	447 (2)	445 (1)	445 (1)	442 (0)	445 (4)	443 (3)	445 (2)	444 (2)	499 (6)	500 (6)
43	412 (10)	410 (9)	408 (1)	406 (1)	414 (4)	413 (4)	408 (2)	406 (2)	445 (1)	445 (1)
44	231 (2)	231 (2)	238 (1)	234 (1)	246 (2)	241 (2)	236 (2)	231 (2)	414 (1)	413 (1)
45	2882 (7)	2835 (6)	2853 (7)	2807 (7)	2851 (10)	2805 (9)	2873 (11)	2827 (10)	243 (0)	239 (0)
46	1160 (0)	1149 (0)	1147 (0)	1134 (0)	1142 (0)	1127 (0)	1143 (2)	1129 (2)	1012 (0)	1007 (0)
47	1019 (0)	1032 (0)	1010 (0)	1027 (0)	1014 (0)	1021 (0)	1013 (0)	1006 (0)	1003 (0)	1001 (0)
48	1011 (0)	1026 (0)	1007 (0)	1022 (0)	1001 (1)	1012 (1)	1008 (0)	1017 (1)	979 (1)	982 (1)
49	995 (3)	995 (3)	987 (7)	1011 (11)	983 (5)	994 (5)	995 (7)	995 (6)	964 (1)	968 (1)
50	983 (3)	993 (4)	964 (4)	986 (0)	956 (14)	980 (4)	981 (0)	976 (0)	944 (1)	953 (0)
51	952 (9)	971 (3)	938 (15)	944 (24)	934 (5)	939 (14)	960 (2)	967 (0)	873 (0)	912 (1)
52	888 (4)	911 (9)	873 (0)	885 (0)	874 (1)	867 (0)	881 (1)	896 (1)	860 (1)	867 (2)
53	863 (5)	861 (3)	818 (21)	959 (5)	812 (3)	963 (0)	867 (1)	864 (3)	792 (127)	838 (4)
54	770 (23)	754 (25)	797 (1)	789 (2)	791 (6)	790 (3)	767 (56)	756 (41)	777 (32)	776 (105)
55	754 (22)	844 (0)	765 (59)	767 (50)	760 (55)	759 (50)	749 (5)	803 (21)	754 (17)	760 (18)
56	712 (46)	712 (47)	732 (2)	776 (0)	729 (16)	748 (5)	730 (41)	719 (15)	730 (11)	726 (32)
57	657 (11)	658 (14)	644 (12)	657 (19)	663 (20)	668 (19)	630 (4)	630 (4)	706 (20)	688 (24)
58	518 (4)	534 (5)	514 (0)	540 (1)	547 (1)	559 (2)	499 (1)	489 (5)	576 (0)	582 (0)
59	489 (0)	499 (0)	462 (16)	486 (15)	487 (0)	489 (0)	469 (9)	467 (7)	529 (0)	525 (0)
60	439 (0)	441 (0)	412 (1)	417 (1)	422 (4)	424 (6)	426 (0)	418 (0)	483 (7)	470 (9)
61	405 (0)	407 (1)	386 (4)	398 (6)	379 (6)	381 (6)	397 (0)	386 (0)	428 (11)	420 (11)
62	284 (9)	291 (11)	283 (0)	298 (0)	297 (1)	304 (1)	293 (11)	284 (11)	400 (0)	393 (0)
63	218 (0)	223 (0)	231 (1)	226 (0)	225 (1)	224 (1)	219 (2)	214 (1)	244 (1)	238 (1)
64	180 (4)	188 (4)	184 (3)	186 (4)	195 (2)	195 (3)	178 (3)	174 (3)	224 (13)	223 (12)
65	109 (2)	108 (2)	95 (2)	92 (2)	99 (1)	100 (1)	99 (3)	94 (3)	105 (1)	102 (1)
66	86 (0)	82 (0)	78 (1)	75 (1)	82 (4)	86 (4)	84 (0)	72 (0)	102 (0)	99 (0)

[a]Harmonic vibrational wavenumbers scaled according to 0.9510 x + 35.9 for wavenumbers > 2000 cm^{-1} and 0.9778 x + 6.3 for wavenumbers < 2000 cm^{-1}. [b]IR intensities in km mol^{-1} are listed in parentheses.

Table S4. Scaled harmonic and anharmonic vibrational wavenumbers and IR intensities of 6-, 7-, 8-, 9-, and 10- $H^+C_{13}H_9N$ calculated with the B3LYP/6-311++G(d,p) method

ν_i	6-$H^+C_{13}H_9N$		7-$H^+C_{13}H_9N$		8-$H^+C_{13}H_9N$		9-$H^+C_{13}H_9N$		10-$H^+C_{13}H_9N$	
	scaled[a]	anharmonic	scaled[a]	anharmonic	scaled[a]	anharmonic	scaled[a]	anharmonic	scaled[a]	anharmonic
1	3094 (0)[b]	3090 (1)[b]	3091 (1)[b]	3091 (0[b])	3102 (0)[b]	3059 (0)[b]	3091 (1)[b]	3063 (1)[b]	3090 (1)[b]	3080 (1)[b]
2	3090 (1)	3096 (0)	3087 (1)	3084 (0)	3091 (1)	3071 (0)	3081 (0)	3116 (2)	3087 (1)	3086 (1)
3	3085 (0)	3072 (1)	3081 (1)	3123 (2)	3087 (0)	3118 (3)	3079 (1)	3074 (2)	3083 (0)	3105 (2)
4	3084 (1)	3131 (3)	3076 (2)	3070 (2)	3078 (0)	3090 (0)	3076 (1)	3101 (1)	3071 (1)	3073 (0)
5	3075 (0)	3059 (0)	3070 (1)	3056 (1)	3069 (1)	3105 (0)	3070 (1)	3051 (1)	3065 (0)	3044 (1)
6	3068 (0)	3050 (0)	3065 (4)	3057 (4)	3068 (1)	3056 (0)	3064 (2)	3092 (0)	3064 (1)	3062 (1)
7	3066 (0)	3062 (0)	3061 (1)	3042 (1)	3051 (1)	3035 (0)	3062 (1)	3033 (3)	3050 (1)	3034 (0)
8	3061 (1)	3074 (0)	3031 (3)	3021 (4)	3021 (1)	3019 (1)	3040 (2)	3042 (2)	3024 (2)	3019 (2)
9	3018 (1)	2984 (0)	2858 (49)	2829 (4)	2856 (56)	2830 (39)	2847 (58)	2815 (42)	2876 (17)	2851 (6)
10	2699 (42)	2648 (26)	1610 (11)	1603 (9)	1636 (164)	1630 (53)	1624 (111)	1613 (79)	1619 (57)	1609 (14)
11	1642 (39)	1634 (7)	1594 (20)	1585 (20)	1602 (466)	1598 (185)	1604 (42)	1595 (4)	1613 (134)	1604 (117)
12	1621 (73)	1610 (29)	1551 (12)	1542 (5)	1600 (46)	1590 (74)	1553 (15)	1545 (13)	1579 (93)	1574 (54)
13	1601 (189)	1594 (134)	1536 (124)	1526 (7)	1552 (13)	1545 (2)	1540 (61)	1531 (45)	1545 (84)	1538 (32)
14	1550 (2)	1543 (2)	1499 (25)	1492 (2)	1532 (5)	1529 (3)	1489 (111)	1482 (80)	1521 (178)	1512 (81)
15	1523 (28)	1516 (1)	1496 (119)	1488 (135)	1479 (110)	1474 (28)	1478 (7)	1473 (1)	1479 (99)	1476 (61)
16	1472 (156)	1467 (47)	1457 (73)	1455 (47)	1469 (134)	1467 (47)	1463 (42)	1459 (29)	1455 (331)	1450 (113)
17	1462 (99)	1459 (5)	1426 (26)	1414 (1)	1437 (36)	1437 (27)	1442 (43)	1439 (3)	1427 (84)	1422 (60)
18	1445 (284)	1437 (21)	1424 (25)	1421 (8)	1410 (6)	1407 (2)	1410 (38)	1405 (4)	1407 (10)	1399 (8)
19	1414 (17)	1412 (5)	1374 (19)	1372 (14)	1388 (1)	1385 (2)	1377 (202)	1371 (73)	1399 (6)	1396 (3)
20	1387 (3)	1380 (1)	1357 (35)	1344 (5)	1372 (92)	1367 (19)	1360 (41)	1343 (21)	1387 (101)	1384 (50)
21	1345 (34)	1339 (3)	1331 (341)	1325 (236)	1353 (40)	1343 (8)	1333 (119)	1329 (101)	1341 (66)	1330 (18)
22	1342 (102)	1336 (53)	1312 (218)	1307 (58)	1324 (50)	1326 (17)	1306 (60)	1304 (2)	1329 (67)	1308 (30)
23	1303 (49)	1298 (9)	1303 (81)	1279 (80)	1310 (140)	1291 (36)	1294 (112)	1278 (72)	1311 (2)	1303 (19)
24	1271 (7)	1267 (11)	1274 (35)	1270 (36)	1284 (9)	1284 (8)	1282 (21)	1282 (34)	1278 (6)	1275 (5)
25	1226 (18)	1224 (7)	1254 (111)	1251 (53)	1249 (30)	1253 (13)	1262 (39)	1258 (17)	1233 (26)	1232 (14)
26	1200 (47)	1199 (5)	1218 (29)	1215 (17)	1219 (21)	1217 (6)	1220 (40)	1216 (30)	1222 (23)	1220 (15)
27	1183 (25)	1187 (15)	1201 (75)	1203 (2)	1190 (27)	1190 (21)	1206 (253)	1208 (155)	1182 (7)	1186 (8)
28	1176 (14)	1181 (18)	1180 (10)	1187 (9)	1175 (5)	1182 (4)	1168 (66)	1172 (17)	1169 (2)	1174 (3)
29	1147 (18)	1146 (12)	1147 (53)	1147 (45)	1159 (20)	1160 (11)	1160 (64)	1165 (71)	1169 (19)	1171 (12)
30	1105 (11)	1090 (10)	1126 (1)	1128 (0)	1117 (11)	1122 (6)	1119 (20)	1119 (18)	1121 (7)	1121 (5)
31	1104 (17)	1106 (1)	1073 (6)	1072 (5)	1037 (6)	1039 (4)	1039 (0)	1039 (1)	1067 (20)	1070 (16)
32	1040 (2)	1044 (2)	1035 (2)	1035 (1)	1024 (17)	1025 (12)	1036 (4)	1034 (2)	1028 (1)	1028 (0)
33	1034 (10)	1016 (3)	996 (11)	997 (8)	993 (12)	997 (10)	992 (11)	993 (7)	996 (10)	1002 (6)
34	1022 (8)	1015 (9)	944 (18)	946 (13)	917 (28)	919 (30)	914 (33)	918 (8)	950 (7)	954 (7)

35	1014 (7)	1018 (10)	875 (10)	878 (6)	858 (12)	863 (8)	869 (16)	869 (14)	853 (9)	856 (8)
36	1012 (1)	1024 (8)	829 (2)	830 (2)	820 (4)	822 (3)	823 (2)	825 (2)	825 (1)	825 (1)
37	1007 (11)	987 (17)	716 (1)	718 (1)	714 (3)	715 (2)	717 (19)	717 (17)	720 (13)	720 (9)
38	984 (0)	996 (1)	687 (4)	687 (3)	692 (8)	695 (7)	697 (1)	698 (1)	687 (16)	687 (14)
39	979 (3)	976 (2)	604 (1)	604 (1)	601 (11)	606 (6)	603 (4)	605 (3)	600 (0)	599 (0)
40	969 (18)	953 (5)	539 (25)	539 (24)	541 (3)	542 (3)	544 (15)	544 (13)	539 (5)	539 (5)
41	900 (6)	893 (2)	497 (2)	497 (1)	495 (4)	496 (4)	499 (4)	499 (3)	497 (4)	499 (2)
42	893 (7)	900 (5)	445 (8)	443 (8)	445 (1)	446 (1)	443 (4)	441 (4)	444 (2)	442 (2)
43	853 (24)	859 (5)	406 (4)	403 (4)	413 (3)	415 (3)	409 (5)	407 (5)	412 (10)	410 (10)
44	836 (23)	821 (35)	236 (3)	229 (2)	246 (1)	244 (1)	238 (1)	234 (1)	232 (1)	227 (1)
45	813 (3)	814 (2)	2861 (9)	2813 (9)	2856 (8)	2811 (7)	2843 (10)	2795 (9)	2886 (6)	2841 (6)
46	782 (18)	785 (6)	1150 (1)	1137 (1)	1151 (0)	1138 (0)	1139 (0)	1123 (0)	1167 (0)	1158 (0)
47	778 (39)	778 (36)	1014 (1)	1036 (1)	1013 (0)	1028 (2)	1008 (0)	1021 (0)	1018 (0)	1024 (0)
48	741 (66)	742 (62)	1008 (0)	1012 (0)	1004 (2)	1019 (0)	1003 (0)	1014 (0)	1010 (0)	1025 (1)
49	705 (25)	706 (15)	989 (3)	994 (4)	979 (3)	991 (2)	977 (3)	989 (3)	1000 (2)	996 (2)
50	684 (29)	686 (29)	977 (1)	981 (1)	952 (17)	978 (13)	944 (28)	968 (13)	980 (1)	987 (2)
51	669 (1)	669 (1)	929 (6)	952 (1)	911 (1)	928 (0)	916 (1)	920 (1)	925 (15)	945 (12)
52	606 (2)	607 (1)	874 (4)	924 (5)	876 (4)	874 (1)	873 (0)	946 (2)	887 (0)	923 (1)
53	566 (0)	565 (0)	855 (8)	856 (3)	812 (7)	1007 (6)	806 (1)	866 (1)	862 (6)	863 (3)
54	536 (1)	536 (1)	778 (44)	763 (62)	795 (3)	796 (1)	781 (32)	792 (9)	771 (41)	761 (42)
55	509 (3)	507 (2)	754 (24)	811 (1)	766 (59)	766 (29)	769 (46)	763 (46)	767 (8)	821 (13)
56	491 (4)	491 (4)	714 (24)	722 (17)	743 (24)	752 (50)	738 (0)	752 (1)	721 (50)	717 (41)
57	459 (2)	457 (2)	627 (12)	634 (15)	634 (5)	657 (8)	634 (22)	640 (27)	627 (0)	630 (0)
58	444 (5)	441 (3)	523 (0)	536 (0)	556 (2)	577 (4)	525 (0)	543 (0)	537 (0)	546 (1)
59	414 (7)	413 (5)	484 (1)	485 (1)	474 (1)	497 (1)	503 (1)	521 (2)	482 (0)	498 (0)
60	387 (3)	384 (1)	430 (4)	441 (3)	424 (11)	428 (14)	416 (4)	419 (4)	439 (5)	448 (4)
61	366 (3)	363 (3)	391 (1)	391 (3)	378 (0)	383 (0)	394 (2)	399 (3)	405 (5)	403 (8)
62	251 (4)	246 (4)	281 (2)	288 (4)	302 (5)	311 (4)	278 (4)	294 (2)	277 (2)	279 (2)
63	210 (2)	205 (2)	220 (4)	218 (4)	224 (0)	227 (0)	229 (1)	229 (1)	225 (1)	218 (0)
64	196 (1)	192 (1)	169 (3)	172 (2)	188 (0)	196 (0)	191 (5)	184 (6)	172 (8)	179 (9)
65	97 (0)	90 (0)	100 (3)	92 (3)	97 (0)	105 (0)	100 (1)	97 (2)	106 (0)	107 (0)
66	84 (2)	78 (2)	83 (1)	76 (1)	82 (5)	81 (5)	79 (0)	81 (0)	84 (0)	80 (0)

[a]Harmonic vibrational wavenumbers scaled according to 0.9510 x + 35.9 for wavenumbers > 2000 cm^{-1} and 0.9778 x + 6.3 for wavenumbers < 2000 cm^{-1}. [b]IR intensities in km mol^{-1} are listed in parentheses.

Table S5. Scaled harmonic and anharmonic vibrational wavenumbers and IR intensities of 4a-, 6a-, 10a-, and 10b-$H^+C_{13}H_9N$ calculated with the B3LYP/6-311++G(d,p) method

ν_i	4a-$H^+C_{13}H_9N$		6a-$H^+C_{13}H_9N$		10a-$H^+C_{13}H_9N$		10b-$H^+C_{13}H_9N$	
	scaled[a]	anharmonic	scaled[a]	anharmonic	scaled[a]	anharmonic	scaled[a]	anharmonic
1	3092 (1)[b]	3107 (0)[b]	3094 (0)[b]	3090 (1)[b]	3092 (1)[b]	3073 (1)[b]	3091 (1)[b]	3124 (1)[b]
2	3089 (2)	3055 (1)	3090 (1)	3096 (0)	3090 (1)	3086 (1)	3088 (2)	3083 (0)
3	3085 (0)	3119 (0)	3085 (0)	3072 (1)	3082 (0)	3107 (0)	3086 (1)	3082 (0)
4	3079 (1)	3102 (1)	3084 (1)	3131 (3)	3079 (2)	3115 (2)	3081 (1)	3104 (0)
5	3076 (3)	3093 (0)	3075 (0)	3059 (0)	3074 (1)	3063 (0)	3074 (0)	3054 (1)
6	3071 (0)	3083 (1)	3068 (0)	3050 (0)	3072 (0)	3081 (2)	3068 (0)	3065 (1)
7	3064 (0)	3042 (0)	3066 (0)	3062 (0)	3065 (0)	3039 (0)	3065 (1)	3051 (0)
8	3063 (0)	3067 (3)	3061 (1)	3074 (0)	3063 (1)	3045 (0)	3063 (0)	3028 (1)
9	3015 (4)	2960 (1)	3018 (1)	2984 (0)	3034 (1)	3022 (2)	3030 (1)	3025 (1)
10	2655 (46)	2589 (35)	2699 (42)	2648 (26)	2647 (36)	2584 (26)	2696 (31)	2638 (21)
11	1641 (26)	1629 (13)	1642 (39)	1634 (7)	1610 (17)	1600 (0)	1623 (40)	1614 (7)
12	1613 (51)	1605 (0)	1621 (73)	1610 (29)	1605 (74)	1597 (53)	1608 (95)	1600 (1)
13	1599 (84)	1592 (24)	1602 (189)	1594 (134)	1557 (10)	1550 (2)	1579 (71)	1571 (62)
14	1559 (6)	1551 (3)	1550 (2)	1543 (2)	1542 (23)	1532 (8)	1551 (126)	1542 (39)
15	1525 (10)	1517 (5)	1523 (28)	1516 (1)	1497 (6)	1495 (2)	1506 (38)	1500 (10)
16	1472 (7)	1468 (4)	1472 (156)	1467 (47)	1494 (65)	1487 (15)	1498 (101)	1493 (1)
17	1466 (112)	1461 (1)	1462 (99)	1459 (5)	1462 (9)	1459 (2)	1458 (41)	1457 (13)
18	1457 (514)	1450 (100)	1445 (284)	1437 (21)	1427 (20)	1426 (2)	1427 (47)	1424 (0)
19	1408 (11)	1405 (5)	1414 (17)	1412 (5)	1396 (22)	1391 (0)	1419 (70)	1411 (18)
20	1381 (9)	1375 (1)	1387 (3)	1380 (1)	1389 (453)	1387 (29)	1396 (601)	1392 (1)
21	1351 (17)	1339 (2)	1346 (34)	1339 (3)	1360 (23)	1346 (15)	1344 (108)	1330 (5)
22	1339 (97)	1332 (56)	1342 (102)	1336 (53)	1325 (46)	1316 (3)	1317 (19)	1309 (8)
23	1302 (79)	1296 (14)	1303 (49)	1298 (9)	1290 (3)	1290 (2)	1291 (11)	1292 (2)
24	1272 (20)	1268 (27)	1271 (7)	1267 (11)	1269 (11)	1264 (1)	1271 (11)	1266 (4)
25	1223 (31)	1219 (5)	1226 (18)	1224 (7)	1223 (21)	1219 (23)	1244 (46)	1241 (7)
26	1216 (38)	1215 (18)	1200 (47)	1199 (5)	1215 (21)	1214 (2)	1212 (48)	1213 (10)
27	1181 (13)	1185 (13)	1183 (25)	1187 (15)	1202 (92)	1200 (18)	1207 (45)	1201 (18)
28	1180 (9)	1189 (1)	1176 (14)	1181 (18)	1178 (15)	1185 (8)	1179 (5)	1185 (6)
29	1151 (10)	1150 (7)	1147 (18)	1146 (12)	1161 (154)	1161 (130)	1160 (99)	1162 (68)
30	1114 (2)	1109 (3)	1105 (11)	1090 (10)	1137 (40)	1139 (21)	1144 (6)	1148 (2)
31	1095 (1)	1088 (0)	1104 (17)	1106 (1)	1096 (11)	1094 (9)	1103 (3)	1096 (1)
32	1057 (25)	1034 (5)	1040 (2)	1044 (2)	1042 (9)	1046 (0)	1065 (21)	1056 (3)
33	1042 (6)	1040 (2)	1034 (10)	1016 (3)	1029 (4)	1027 (2)	1040 (3)	1032 (3)
34	1029 (1)	1021 (1)	1022 (8)	1015 (9)	1022 (28)	1015 (7)	1031 (2)	1025 (3)

35	1021 (14)	1029 (4)	1014 (7)	1018 (10)	1015 (18)	1002 (17)	1024 (11)	1010 (3)
36	1013 (0)	1012 (1)	1012 (1)	1024 (8)	1008 (0)	1016 (12)	1011 (0)	1018 (1)
37	1011 (1)	1003 (5)	1007 (11)	987 (17)	1004 (6)	994 (1)	1001 (5)	999 (6)
38	986 (1)	995 (3)	984 (0)	996 (1)	977 (2)	979 (2)	992 (7)	991 (6)
39	981 (2)	974 (0)	979 (3)	976 (2)	955 (12)	945 (14)	976 (12)	972 (3)
40	961 (8)	955 (4)	969 (18)	953 (5)	941 (26)	908 (15)	964 (12)	958 (25)
41	948 (35)	919 (28)	900 (6)	893 (2)	929 (15)	923 (11)	948 (2)	939 (1)
42	898 (4)	887 (7)	893 (7)	900 (5)	886 (3)	878 (6)	902 (7)	898 (5)
43	881 (2)	878 (3)	853 (24)	859 (5)	861 (13)	854 (16)	875 (5)	874 (5)
44	865 (13)	866 (11)	836 (23)	821 (35)	850 (11)	849 (8)	864 (6)	858 (3)
45	796 (44)	777 (33)	813 (3)	814 (2)	820 (1)	820 (0)	814 (3)	816 (1)
46	794 (9)	801 (6)	782 (18)	785 (6)	797 (21)	786 (22)	801 (5)	791 (5)
47	775 (24)	777 (12)	778 (39)	778 (36)	779 (42)	777 (41)	767 (67)	767 (43)
48	743 (37)	744 (33)	741 (66)	742 (62)	729 (67)	733 (18)	728 (21)	735 (5)
49	709 (4)	712 (0)	705 (25)	706 (15)	716 (8)	710 (11)	720 (40)	714 (40)
50	688 (39)	682 (47)	684 (29)	686 (29)	689 (6)	689 (13)	699 (2)	695 (13)
51	679 (3)	680 (3)	669 (1)	669 (1)	669 (3)	665 (2)	676 (3)	674 (3)
52	604 (1)	605 (1)	606 (2)	607 (1)	601 (2)	601 (2)	599 (2)	599 (2)
53	560 (12)	556 (10)	566 (0)	565 (0)	569 (5)	568 (5)	544 (3)	544 (3)
54	532 (4)	531 (4)	536 (1)	536 (1)	535 (31)	505 (29)	528 (11)	528 (9)
55	513 (1)	510 (1)	509 (3)	507 (2)	505 (3)	506 (0)	512 (6)	514 (5)
56	490 (1)	488 (1)	491 (4)	491 (4)	494 (5)	492 (5)	494 (9)	492 (8)
57	455 (4)	453 (3)	459 (2)	457 (2)	467 (4)	462 (3)	440 (11)	441 (6)
58	438 (1)	434 (1)	444 (5)	441 (3)	426 (5)	422 (4)	430 (1)	422 (3)
59	411 (5)	410 (8)	414 (7)	413 (5)	414 (4)	411 (3)	412 (1)	409 (1)
60	399 (8)	392 (4)	387 (3)	384 (1)	402 (2)	398 (3)	394 (0)	391 (0)
61	369 (5)	364 (5)	366 (3)	363 (3)	374 (1)	373 (1)	380 (8)	377 (7)
62	244 (3)	240 (3)	251 (4)	246 (4)	238 (1)	236 (1)	245 (1)	241 (1)
63	220 (1)	214 (1)	210 (2)	205 (2)	223 (2)	221 (2)	218 (0)	215 (0)
64	200 (0)	197 (0)	196 (1)	192 (1)	210 (0)	205 (1)	196 (1)	192 (1)
65	101 (1)	92 (1)	97 (0)	90 (0)	92 (2)	86 (1)	93 (1)	88 (1)
66	88 (2)	82 (2)	84 (2)	78 (2)	92 (0)	85 (2)	70 (1)	63 (0)

[a]Harmonic vibrational wavenumbers scaled according to 0.9510 x + 35.9 for wavenumbers > 2000 cm^{-1} and 0.9778 x + 6.3 for wavenumbers < 2000 cm^{-1}. [b]IR intensities in km mol^{-1} are listed in parentheses.

Table S6. Cartesian coordinates of optimized geometries of isomers of hydrogenated phenanthridine ($HC_{13}H_9N$) predicted with the B3LYP/6-311++G(d,p) method

	x	*y*	*z*	*x*	*y*	*z*
		1-$HC_{13}H_9N$			2-$HC_{13}H_9N$	
C1	-3.560281	-0.284322	-0.000005	-3.593670	-0.277778	-0.000009
C2	-2.843265	0.892357	-0.000029	-2.859272	0.888997	-0.000027
C3	-1.432003	0.870423	-0.000021	-1.446813	0.849704	-0.000021
C4	-0.732513	-0.373729	0.000009	-0.771901	-0.400390	0.000004
C5	-1.500905	-1.570040	0.000034	-1.547604	-1.576421	0.000022
C6	-2.877575	-1.520391	0.000027	-2.929540	-1.518140	0.000016
C7	-0.655691	2.067082	-0.000041	-0.669590	2.049796	-0.000038
C8	0.689010	-0.340524	0.000014	0.689430	-0.383056	0.000009
C9	1.333465	0.906088	-0.000012	1.324675	0.911281	-0.000011
C10	2.758839	0.989263	-0.000016	2.769142	0.987513	-0.000008
C11	3.548066	-0.175538	-0.000007	3.546220	-0.109993	0.000014
C12	2.981309	-1.419671	0.000019	2.968025	-1.491144	0.000039
C13	1.497129	-1.618372	0.000053	1.473202	-1.520838	0.000030
H1	3.203550	1.975859	-0.000034	3.196365	1.983972	-0.000024
H2	-1.173695	3.025597	-0.000065	-1.182493	3.009034	-0.000056
H3	-4.643896	-0.266730	-0.000011	-4.677103	-0.243448	-0.000013
H4	-3.353704	1.850071	-0.000053	-3.356442	1.853527	-0.000046
H5	-1.002872	-2.531507	0.000059	-1.064333	-2.545411	0.000042
H6	-3.447231	-2.443237	0.000046	-3.505617	-2.436654	0.000031
H7	4.628937	-0.079810	-0.000024	4.627357	-0.015937	0.000015
H8	3.603843	-2.307758	0.000020	3.354171	-2.057318	0.865814
H9	1.212377	-2.236063	0.868210	1.010233	-2.500568	0.000043
H10	1.212344	-2.236147	-0.868030	3.354182	-2.057357	-0.865705
N	0.646692	2.101997	-0.000038	0.647121	2.078996	-0.000033
		3-$HC_{13}H_9N$			4-$HC_{13}H_9N$	
C1	1.999509	-2.995017	0.000000	2.019131	-2.979180	0.000000
C2	0.619599	-2.922974	0.000000	0.645611	-2.920916	0.000000
C3	-0.031907	-1.676653	0.000000	-0.020365	-1.670879	0.000000
C4	0.722503	-0.465322	0.000000	0.730412	-0.461147	0.000000
C5	2.138311	-0.571860	0.000000	2.143186	-0.555867	0.000000
C6	2.755140	-1.805983	0.000000	2.769797	-1.783532	0.000000
C7	-1.465644	-1.580678	0.000000	-1.438355	-1.580079	0.000000
C8	0.000000	0.767388	0.000000	0.000000	0.785806	0.000000
C9	-1.434066	0.725196	0.000000	-1.407308	0.716262	0.000000
C10	-2.179286	1.893322	0.000000	-2.254342	1.966513	0.000000
C11	-1.560162	3.249969	0.000000	-1.482808	3.246238	0.000000
C12	-0.062925	3.217067	0.000000	-0.117983	3.256400	0.000000
C13	0.636999	2.065238	0.000000	0.635563	2.062748	0.000000
H1	-3.259112	1.801948	0.000000	-2.934215	1.926594	0.865361
H2	-2.040832	-2.506796	0.000000	-2.028726	-2.494158	0.000000
H3	2.500063	-3.956099	0.000000	2.528274	-3.936085	0.000000
H4	0.020971	-3.828329	0.000000	0.052913	-3.829847	0.000000
H5	2.750046	0.320938	0.000000	2.747044	0.342291	0.000000
H6	3.838213	-1.861390	0.000000	3.853014	-1.832590	0.000000
H7	-1.921408	3.833375	0.865537	-2.039994	4.176677	0.000000
H8	0.461517	4.167144	0.000000	0.411414	4.203710	0.000000
H9	1.719308	2.115763	0.000000	1.715010	2.129512	0.000000
H10	-1.921408	3.833375	-0.865537	-2.934215	1.926594	-0.865361
N	-2.139398	-0.474013	0.000000	-2.100822	-0.443841	0.000000

	x	*y*	*z*	*x*	*y*	*z*
		$C_{13}H_9NH$			6-$HC_{13}H_9N$	
C1	-3.572896	-0.291682	0.000000	2.020560	-2.941107	0.000000
C2	-2.849232	0.881680	0.000000	0.633529	-2.869153	0.000000
C3	-1.424868	0.863407	0.000000	-0.021800	-1.635025	0.000000
C4	-0.749626	-0.405939	0.000000	0.724544	-0.443007	0.000000
C5	-1.520333	-1.571581	0.000000	2.130197	-0.531892	0.000000
C6	-2.913199	-1.531625	0.000000	2.770300	-1.761500	0.000000
C7	-0.683828	2.048914	0.000000	-1.530144	-1.587519	0.000000
C8	0.724086	-0.411338	0.000000	0.000000	0.833232	0.000000
C9	1.419212	0.824658	0.000000	-1.445673	0.801944	0.000000
C10	2.821039	0.861235	0.000000	-2.155949	2.046025	0.000000
C11	3.552928	-0.318213	0.000000	-1.491337	3.249486	0.000000
C12	2.890267	-1.545951	0.000000	-0.084701	3.269478	0.000000
C13	1.499161	-1.580648	0.000000	0.635351	2.078024	0.000000
H1	3.327794	1.821391	0.000000	-3.237767	1.989889	0.000000
H2	-1.138039	3.028665	0.000000	-1.915445	-2.148182	-0.866593
H3	-4.656929	-0.255883	0.000000	2.517104	-3.904771	0.000000
H4	-3.358987	1.839442	0.000000	0.045426	-3.782178	0.000000
H5	-1.032783	-2.538684	0.000000	2.731577	0.368168	0.000000
H6	-3.481677	-2.453804	0.000000	3.853514	-1.805104	0.000000
H7	4.636144	-0.278942	0.000000	-2.046638	4.180603	0.000000
H8	3.453454	-2.471535	0.000000	0.444692	4.215311	0.000000
H9	1.005190	-2.543783	0.000000	1.716886	2.131659	0.000000
H10	1.210405	2.872739	0.000000	-1.915445	-2.148182	0.866593
N	0.697024	2.006129	0.000000	-2.186167	-0.307304	0.000000
		7-$HC_{13}H_9N$			8-$HC_{13}H_9N$	
C1	-1.467759	3.258437	0.000000	3.608021	-0.235456	0.000049
C2	-2.250397	1.984479	0.000000	2.725645	0.969630	0.000032
C3	-1.402362	0.733659	0.000000	1.342511	0.878661	0.000015
C4	0.000000	0.792460	0.000000	0.674738	-0.383825	-0.000016
C5	0.646773	2.064567	0.000000	1.493153	-1.575941	-0.000066
C6	-0.102413	3.260356	0.000000	2.840108	-1.523794	-0.000047
C7	-2.030289	-0.529733	0.000000	0.520670	2.067235	0.000021
C8	0.719167	-0.461534	0.000000	-0.754582	-0.392668	-0.000011
C9	-0.041044	-1.670093	0.000000	-1.439350	0.864714	0.000008
C10	0.630851	-2.917749	0.000000	-2.846046	0.897255	0.000011
C11	2.004446	-2.976354	0.000000	-3.581414	-0.272182	0.000001
C12	2.760200	-1.785118	0.000000	-2.918833	-1.514490	-0.000012
C13	2.131438	-0.558828	0.000000	-1.540900	-1.574487	-0.000018
H1	0.020315	-3.812736	0.000000	-3.324268	1.869497	0.000023
H2	-3.119201	-0.572338	0.000000	1.022556	3.034630	0.000041
H3	-2.018850	4.192435	0.000000	4.292874	-0.209079	-0.865859
H4	-2.929874	1.970071	0.867628	3.192448	1.949513	0.000046
H5	1.726004	2.125725	0.000000	1.010406	-2.545451	-0.000133
H6	0.429690	4.206096	0.000000	3.413432	-2.445356	-0.000095
H7	2.509951	-3.935478	0.000000	-4.664707	-0.235866	0.000004
H8	3.843258	-1.834665	0.000000	-3.495867	-2.432503	-0.000016
H9	2.735407	0.339677	0.000000	-1.057851	-2.543405	-0.000022
H10	-2.929874	1.970071	-0.867628	4.292743	-0.209141	0.866066
N	-1.408355	-1.688022	0.000000	-0.774871	2.077037	0.000019

	x	*y*	*z*	*x*	*y*	*z*
		$9\text{-}HC_{13}H_9N$			$10\text{-}HC_{13}H_9N$	
C1	-3.553170	-0.109018	0.000030	-1.425738	3.262423	0.000000
C2	-2.771695	0.986569	-0.000038	-2.117392	2.036881	0.000000
C3	-1.330692	0.898143	-0.000042	-1.399266	0.802839	0.000000
C4	-0.696653	-0.387013	0.000022	0.000000	0.776621	0.000000
C5	-1.480688	-1.527074	0.000105	0.785133	2.067967	0.000000
C6	-2.974979	-1.490273	0.000103	-0.058812	3.306776	0.000000
C7	-0.527846	2.058127	-0.000094	-2.095532	-0.449696	0.000000
C8	0.763347	-0.394060	0.000007	0.652564	-0.487480	0.000000
C9	1.439374	0.860695	-0.000039	-0.156414	-1.668761	0.000000
C10	2.852750	0.889002	-0.000044	0.462857	-2.938512	0.000000
C11	3.583382	-0.279765	-0.000011	1.835637	-3.051544	0.000000
C12	2.919641	-1.519752	0.000025	2.640714	-1.892167	0.000000
C13	1.538173	-1.571194	0.000033	2.065539	-0.641048	0.000000
H1	3.331678	1.860790	-0.000077	-0.181030	-3.809891	0.000000
H2	-1.020386	3.029625	-0.000136	-3.184726	-0.433686	0.000000
H3	-4.633956	-0.014002	0.000031	-1.990717	4.188678	0.000000
H4	-3.217997	1.976465	-0.000090	-3.200886	2.016523	0.000000
H5	-1.020287	-2.507514	0.000178	1.464741	2.086563	0.867717
H6	-3.362036	-2.056838	-0.865077	0.457471	4.260542	0.000000
H7	4.666999	-0.245759	-0.000015	2.301670	-4.030310	0.000000
H8	3.492607	-2.440131	0.000044	3.720682	-1.988831	0.000000
H9	1.051021	-2.538657	0.000055	2.700799	0.236247	0.000000
H10	0.786962	2.072350	-0.000087	1.464741	2.086563	-0.867717
N	-3.362038	-2.056753	0.865338	-1.526926	-1.622600	0.000000
		$4a\text{-}HC_{13}H_9N$			$6a\text{-}HC_{13}H_9N$	
C1	-3.571603	-0.193004	-0.044848	3.529652	-0.275858	-0.114176
C2	-2.775136	0.941605	-0.139936	2.848413	0.857264	0.191315
C3	-1.381833	0.840113	-0.076447	1.376680	0.826407	0.486602
C4	-0.759982	-0.426774	0.072335	0.693873	-0.466416	0.103507
C5	-1.585437	-1.565679	0.152464	1.460450	-1.585305	-0.178982
C6	-2.966850	-1.447486	0.100893	2.855315	-1.524673	-0.252423
C7	-0.531458	2.020606	-0.241070	0.613864	2.021669	-0.059414
C8	0.687600	-0.458826	0.099984	-0.754342	-0.417867	0.067432
C9	1.374118	0.843871	0.456803	-1.376183	0.850403	-0.098794
C10	2.842429	0.858709	0.146616	-2.769539	0.946267	-0.152721
C11	3.524446	-0.281678	-0.123603	-3.563609	-0.187685	-0.032736
C12	2.846527	-1.532595	-0.228873	-2.962683	-1.441883	0.121748
C13	1.453166	-1.587485	-0.154717	-1.580529	-1.554296	0.164469
H1	3.331178	1.824866	0.190782	-3.205574	1.927422	-0.298619
H2	-1.018622	2.939427	-0.574626	1.175026	2.942266	-0.226967
H3	-4.651208	-0.109320	-0.086192	4.600342	-0.233269	-0.283758
H4	-3.229248	1.919018	-0.268978	3.360515	1.810062	0.280239
H5	-1.140747	-2.545004	0.281981	0.967611	-2.523788	-0.408493
H6	-3.582946	-2.336308	0.180264	3.421238	-2.415933	-0.494294
H7	4.594125	-0.244723	-0.300298	-4.643457	-0.101065	-0.066644
H8	3.408886	-2.431715	-0.450187	-3.577113	-2.330111	0.216492
H9	0.960081	-2.531410	-0.361247	-1.130814	-2.530102	0.306591
H10	1.295449	0.941618	1.565229	1.268055	0.928366	1.594052
N	0.728162	2.072184	-0.042048	-0.637714	2.033998	-0.299079

	x	y	z	x	y	z
		10a-$HC_{13}H_9N$			10b-$HC_{13}H_9N$	
C1	-3.508604	-0.246430	-0.267803	-3.507893	-0.277395	-0.303646
C2	-2.776850	0.924396	-0.132562	-2.779959	0.896367	-0.159624
C3	-1.404611	0.894863	0.121577	-1.398802	0.850628	0.090192
C4	-0.735209	-0.403953	0.504017	-0.745332	-0.395575	0.200890
C5	-1.528797	-1.613211	0.086274	-1.483878	-1.564021	0.041555
C6	-2.845054	-1.514477	-0.220953	-2.857308	-1.508269	-0.208732
C7	-0.598344	2.064825	0.067778	-0.605607	2.063049	0.164538
C8	0.752614	-0.391210	0.179279	0.731334	-0.352234	0.587819
C9	1.386881	0.865228	0.033482	1.387317	0.926365	0.091347
C10	2.774847	0.917676	-0.178413	2.730680	0.922739	-0.300340
C11	3.528413	-0.245106	-0.247877	3.447048	-0.255816	-0.413717
C12	2.902254	-1.483752	-0.103128	2.817555	-1.514687	-0.145280
C13	1.525415	-1.548808	0.112015	1.538419	-1.586123	0.291458
H1	3.229450	1.894984	-0.288815	3.176743	1.877533	-0.554775
H2	-1.100136	3.029848	-0.008009	-1.123579	3.019863	0.187509
H3	-4.568127	-0.204877	-0.489570	-4.574167	-0.237728	-0.494486
H4	-3.258651	1.885147	-0.288514	-3.270477	1.860366	-0.248425
H5	-1.048509	-2.583165	0.110838	-1.003259	-2.531705	0.113105
H6	-3.409176	-2.409161	-0.462091	-3.417613	-2.428808	-0.327191
H7	4.598374	-0.190582	-0.413401	4.478154	-0.235290	-0.745570
H8	3.481589	-2.398545	-0.155474	3.381721	-2.427405	-0.305587
H9	1.062393	-2.521250	0.231388	1.093715	-2.550568	0.505324
H10	-0.770114	-0.416521	1.620308	0.719085	-0.251417	1.698062
N	0.706453	2.084838	0.060173	0.699746	2.096429	0.079039

Table S7. Scaled harmonic and anharmonic vibrational wavenumbers and IR intensities of 1-, 2-, 3-, 4-$C_{13}H_9N$, and $C_{13}H_9NH$ calculated with the B3LYP/6-311++G(d,p) method

ν_i	1-$HC_{13}H_9N$		2-$HC_{13}H_9N$		3-$HC_{13}H_9N$		4-$HC_{13}H_9N$		$C_{13}H_9NH$	
	scaled[a]	anharmonic	scaled[a]	anharmonic	scaled[a]	anharmonic	scaled[a]	anharmonic	scaled[a]	anharmonic
1	3084 (5)[b]	3086 (9)[b]	3075 (17)[b]	3085 (9)[b]	3079 (18)[b]	3088 (12)[b]	3086 (16)[b]	3111 (11)[b]	3500 (60)[b]	3468 (43)[b]
2	3074 (11)	3075 (16)	3065 (23)	3078 (30)	3068 (24)	3064 (9)	3073 (5)	3060 (2)	3100 (7)	3097 (7)
3	3066 (19)	3054 (0)	3065 (12)	3065 (1)	3065 (3)	3052 (25)	3065 (16)	3058 (11)	3080 (18)	3084 (15)
4	3056 (25)	3046 (32)	3054 (5)	3056 (4)	3061 (5)	3047 (2)	3056 (30)	3047 (57)	3072 (19)	3073 (33)
5	3049 (9)	3044 (18)	3049 (12)	3047 (8)	3049 (14)	3038 (5)	3051 (10)	3044 (2)	3066 (3)	3059 (13)
6	3042 (4)	3030 (4)	3042 (1)	3033 (1)	3041 (3)	3014 (19)	3042 (1)	3026 (13)	3058 (28)	3053 (25)
7	3038 (7)	3023 (0)	3040 (7)	3010 (7)	3039 (7)	2999 (14)	3037 (7)	3022 (3)	3053 (15)	3047 (0)
8	2999 (25)	2991 (11)	3016 (15)	3002 (23)	2991 (26)	2996 (3)	3012 (14)	3001 (13)	3050 (2)	3043 (3)
9	2831 (27)	2771 (9)	2823 (55)	2752 (20)	2819 (60)	2811 (25)	2855 (22)	2800 (10)	3037 (6)	3023 (5)
10	1616 (29)	1607 (8)	1631 (0)	1616 (0)	1621 (5)	1606 (1)	1621 (10)	1612 (0)	3034 (10)	3014 (13)
11	1584 (27)	1575 (4)	1611 (4)	1601 (2)	1600 (23)	1591 (7)	1567 (5)	1555 (3)	1608 (25)	1597 (5)
12	1558 (18)	1550 (3)	1562 (2)	1552 (1)	1586 (27)	1580 (21)	1557 (18)	1552 (9)	1586 (2)	1579 (2)
13	1531 (7)	1516 (2)	1517 (8)	1506 (4)	1539 (15)	1528 (8)	1539 (6)	1521 (3)	1580 (25)	1575 (8)
14	1504 (16)	1496 (9)	1486 (5)	1479 (2)	1500 (16)	1493 (11)	1487 (6)	1479 (0)	1538 (0)	1532 (1)
15	1452 (2)	1449 (0)	1475 (3)	1471 (3)	1456 (4)	1452 (1)	1454 (0)	1450 (0)	1516 (2)	1511 (4)
16	1421 (9)	1421 (5)	1430 (10)	1429 (2)	1428 (5)	1424 (3)	1431 (2)	1426 (1)	1510 (41)	1502 (32)
17	1415 (8)	1408 (4)	1412 (8)	1399 (1)	1414 (4)	1416 (2)	1411 (5)	1404 (0)	1468 (56)	1465 (30)
18	1415 (2)	1389 (2)	1405 (2)	1395 (6)	1406 (3)	1392 (1)	1407 (13)	1393 (12)	1444 (1)	1442 (1)
19	1387 (0)	1383 (0)	1376 (4)	1373 (2)	1393 (3)	1377 (3)	1386 (3)	1378 (1)	1437 (26)	1434 (18)
20	1370 (2)	1362 (0)	1345 (1)	1340 (1)	1360 (8)	1358 (3)	1363 (2)	1353 (1)	1415 (18)	1410 (13)
21	1365 (3)	1352 (2)	1336 (4)	1316 (3)	1349 (1)	1340 (0)	1346 (0)	1340 (0)	1338 (19)	1329 (11)
22	1310 (6)	1303 (0)	1303 (21)	1297 (4)	1334 (12)	1319 (4)	1297 (1)	1292 (1)	1306 (64)	1303 (7)
23	1292 (36)	1279 (34)	1272 (2)	1270 (1)	1293 (1)	1282 (1)	1278 (36)	1271 (18)	1290 (0)	1292 (1)
24	1268 (1)	1266 (0)	1269 (4)	1268 (4)	1271 (1)	1273 (1)	1267 (11)	1267 (24)	1282 (29)	1281 (22)
25	1235 (24)	1231 (19)	1251 (5)	1248 (3)	1228 (11)	1227 (6)	1239 (8)	1235 (6)	1259 (7)	1257 (3)
26	1201 (3)	1202 (3)	1214 (7)	1211 (3)	1221 (16)	1219 (0)	1198 (2)	1199 (1)	1227 (10)	1229 (2)
27	1163 (1)	1165 (0)	1197 (0)	1200 (0)	1169 (2)	1173 (2)	1186 (1)	1184 (1)	1203 (17)	1198 (13)
28	1154 (3)	1154 (3)	1150 (0)	1153 (0)	1152 (0)	1150 (0)	1162 (1)	1160 (1)	1165 (11)	1171 (1)
29	1142 (3)	1142 (2)	1143 (6)	1147 (2)	1141 (2)	1138 (1)	1148 (1)	1148 (1)	1152 (10)	1158 (10)
30	1113 (0)	1114 (0)	1121 (0)	1123 (1)	1098 (2)	1097 (1)	1113 (5)	1117 (4)	1137 (19)	1139 (4)
31	1053 (2)	1053 (1)	1037 (4)	1037 (3)	1038 (12)	1039 (3)	1059 (4)	1057 (2)	1132 (7)	1130 (17)
32	1030 (8)	1030 (7)	1020 (5)	1021 (2)	1023 (2)	1023 (1)	1032 (6)	1032 (4)	1099 (7)	1100 (4)
33	989 (0)	1001 (0)	984 (1)	1015 (0)	988 (1)	1001 (0)	983 (2)	1007 (0)	1049 (6)	1047 (4)

34	936 (15)	942 (49)	902 (15)	910 (8)	898 (20)	897 (13)	935 (15)	937 (5)	1037 (8)	1038 (4)
35	865 (7)	877 (4)	867 (10)	867 (9)	872 (14)	896 (1)	882 (7)	883 (6)	989 (18)	995 (7)
36	822 (1)	822 (1)	810 (7)	839 (0)	807 (2)	823 (0)	824 (5)	825 (2)	871 (2)	880 (11)
37	720 (2)	721 (1)	714 (3)	715 (3)	719 (1)	720 (1)	711 (4)	711 (4)	823 (6)	817 (15)
38	695 (1)	696 (1)	703 (0)	704 (0)	694 (1)	695 (1)	697 (0)	698 (0)	716 (3)	713 (1)
39	594 (2)	591 (1)	606 (5)	607 (5)	591 (7)	592 (5)	596 (2)	597 (1)	707 (2)	697 (0)
40	533 (0)	536 (2)	542 (2)	545 (2)	540 (0)	540 (0)	542 (0)	542 (0)	621 (4)	622 (4)
41	494 (1)	494 (1)	492 (2)	492 (1)	492 (1)	492 (1)	492 (0)	492 (0)	550 (1)	550 (2)
42	451 (1)	449 (1)	438 (2)	441 (1)	448 (2)	447 (2)	443 (2)	443 (1)	502 (3)	500 (4)
43	408 (1)	406 (1)	409 (1)	407 (1)	409 (0)	409 (0)	405 (1)	403 (1)	434 (0)	463 (1)
44	229 (1)	224 (1)	239 (1)	234 (1)	239 (1)	235 (1)	239 (1)	234 (1)	410 (1)	358 (38)
45	2828 (15)	2770[e] (19)	2812 (15)	2746 (38)	2806 (14)	2743 (19)	2855 (9)	2836 (14)	244 (1)	240 (1)
46	1184 (0)	1173 (0)	1167 (0)	1157 (0)	1162 (0)	1154 (1)	1170 (1)	1167 (1)	955 (0)	984 (0)
47	978 (0)	991 (0)	981 (0)	986 (1)	974 (0)	990 (1)	979 (0)	985 (0)	952 (0)	972 (0)
48	957 (6)	977 (5)	974 (0)	985 (1)	965 (0)	972 (2)	956 (1)	971 (2)	921 (3)	937 (0)
49	955 (0)	947 (2)	949 (2)	972 (1)	953 (6)	969 (0)	951 (2)	952 (1)	914 (0)	934 (2)
50	927 (3)	932 (5)	919 (12)	928 (1)	926 (8)	941 (4)	925 (0)	915 (0)	836 (0)	841 (3)
51	923 (7)	918 (2)	874 (12)	902 (14)	913 (7)	907 (12)	895 (12)	905 (2)	829 (3)	834 (1)
52	851 (0)	865 (7)	848 (8)	866 (17)	848 (0)	874 (13)	851 (2)	869 (8)	761 (1)	749 (23)
53	806 (4)	822 (0)	793 (10)	810 (4)	790 (0)	809 (1)	793 (2)	814 (2)	743 (110)	737 (34)
54	758 (15)	783 (2)	757 (7)	763 (10)	755 (15)	759 (15)	753 (45)	761 (15)	728 (2)	723 (162)
55	749 (34)	743 (39)	739 (60)	747 (23)	732 (33)	745 (0)	740 (4)	744 (27)	714 (51)	705 (4)
56	682 (38)	687 (6)	719 (4)	716 (32)	723 (64)	720 (87)	669 (45)	674 (15)	605 (10)	611 (6)
57	661 (35)	650 (58)	659 (18)	661 (10)	669 (5)	669 (5)	654 (29)	645 (50)	564 (2)	561 (3)
58	537 (1)	536 (6)	534 (13)	538 (12)	541 (0)	549 (0)	544 (3)	544 (3)	515 (5)	502 (3)
59	525 (6)	533 (0)	516 (0)	529 (0)	517 (0)	525 (0)	507 (0)	501 (0)	476 (0)	496 (18)
60	470 (0)	469 (0)	433 (1)	436 (1)	435 (7)	439 (7)	471 (1)	460 (1)	428 (4)	435 (0)
61	420 (0)	416 (0)	414 (3)	416 (4)	399 (4)	396 (4)	416 (0)	410 (0)	413 (72)	407 (0)
62	301 (5)	299 (5)	270 (0)	271 (1)	293 (0)	297 (0)	303 (5)	293 (5)	319 (2)	321 (7)
63	220 (0)	220 (0)	235 (0)	229 (0)	225 (1)	222 (1)	225 (0)	220 (0)	241 (2)	238 (0)
64	167 (1)	166 (1)	177 (1)	173 (0)	180 (0)	179 (0)	146 (1)	152 (1)	221 (6)	216 (7)
65	122 (0)	115 (0)	103 (2)	103 (2)	107 (1)	104 (1)	98 (1)	94 (1)	102 (1)	100 (1)
66	86 (0)	71 (0)	80 (0)	73 (0)	78 (1)	78 (1)	83 (0)	74 (1)	78 (0)	74 (0)

[a]Harmonic vibrational wavenumbers scaled according to 0.9510 x + 35.9 for wavenumbers > 2000 cm^{-1} and 0.9778 x + 6.3 for wavenumbers < 2000 cm^{-1}. [b]IR intensities in km mol^{-1} are listed in parentheses.

Table S8. Scaled harmonic and anharmonic vibrational wavenumbers and IR intensities of 6-, 7-, 8-, 9-, and 10-$C_{13}H_9N$ calculated with the B3LYP/6-311++G(d,p) method

ν_i	6-$HC_{13}H_9N$		7-$HC_{13}H_9N$		8-$HC_{13}H_9N$		9-$HC_{13}H_9N$		10-$HC_{13}H_9N$	
	scaled[a]	anharmonic	scaled[a]	anharmonic	scaled[a]	anharmonic	scaled[a]	anharmonic	scaled[a]	anharmonic
1	3080 (18)[b]	3110 (11)[b]	3091 (12)[b]	3112 (5)[b]	3078 (20)[b]	3095 (11)[b]	3076 (15)[b]	3128 (5)[b]	3075 (13)[b]	3090 (7)[b]
2	3076 (17)	3085 (9)	3075 (13)	3108 (9)	3074 (22)	3081 (5)	3071 (20)	3061 (16)	3070 (15)	3084 (10)
3	3069 (10)	3079 (9)	3070 (9)	3064 (9)	3065 (3)	3073 (13)	3062 (2)	3058 (4)	3066 (19)	3070 (7)
4	3064 (7)	3068 (0)	3058 (3)	3055 (3)	3058 (7)	3051 (27)	3055 (4)	3046 (2)	3058 (8)	3059 (11)
5	3058 (19)	3043 (7)	3057 (32)	3035 (3)	3046 (4)	3037 (24)	3053 (31)	3040 (1)	3054 (18)	3049 (2)
6	3049 (8)	3031 (2)	3046 (2)	3030 (4)	3041 (11)	3020 (10)	3045 (1)	3014 (0)	3046 (2)	3025 (7)
7	3047 (0)	3030 (7)	3038 (6)	3023 (5)	3039 (8)	2997 (10)	3033 (6)	2996 (1)	3039 (4)	3014 (16)
8	3033 (9)	3001 (17)	2995 (28)	2974 (22)	2995 (22)	2988 (23)	3001 (26)	2989 (20)	3001 (23)	2985 (19)
9	2849 (21)	2760 (5)	2842 (25)	2831 (4)	2820 (60)	2746 (33)	2824 (41)	2814 (16)	2838 (24)	2837 (6)
10	1610 (1)	1599 (1)	1619 (1)	1610 (2)	1622 (4)	1611 (2)	1626 (6)	1611 (1)	1615 (0)	1606 (0)
11	1584 (2)	1576 (1)	1567 (17)	1556 (1)	1600 (1)	1589 (1)	1613 (0)	1603 (0)	1579 (8)	1566 (2)
12	1575 (11)	1566 (4)	1562 (11)	1553 (9)	1572 (17)	1559 (8)	1556 (6)	1550 (2)	1562 (7)	1554 (3)
13	1526 (4)	1520 (1)	1541 (6)	1528 (4)	1536 (0)	1527 (0)	1524 (6)	1512 (2)	1529 (1)	1520 (0)
14	1491 (6)	1485 (6)	1491 (21)	1483 (18)	1507 (16)	1499 (6)	1478 (21)	1471 (14)	1509 (27)	1502 (19)
15	1465 (7)	1462 (5)	1461 (1)	1458 (1)	1458 (3)	1454 (3)	1470 (3)	1465 (1)	1465 (2)	1460 (1)
16	1451 (17)	1447 (9)	1427 (5)	1425 (1)	1431 (2)	1430 (2)	1426 (4)	1422 (2)	1425 (4)	1417 (0)
17	1417 (10)	1403 (2)	1417 (1)	1411 (0)	1416 (10)	1427 (1)	1421 (0)	1411 (0)	1418 (6)	1406 (1)
18	1404 (9)	1398 (3)	1412 (7)	1390 (1)	1412 (3)	1387 (9)	1407 (10)	1392 (0)	1411 (3)	1397 (3)
19	1358 (11)	1348 (8)	1396 (3)	1388 (1)	1389 (1)	1384 (10)	1397 (1)	1389 (7)	1391 (2)	1389 (0)
20	1335 (1)	1323 (0)	1358 (8)	1351 (3)	1385 (12)	1374 (4)	1339 (2)	1324 (7)	1376 (3)	1367 (2)
21	1309 (0)	1305 (0)	1343 (5)	1333 (3)	1341 (1)	1334 (2)	1332 (13)	1321 (3)	1355 (5)	1347 (1)
22	1299 (1)	1288 (1)	1296 (19)	1290 (4)	1333 (8)	1315 (4)	1321 (7)	1309 (6)	1302 (5)	1290 (5)
23	1281 (2)	1283 (2)	1281 (19)	1273 (29)	1288 (1)	1278 (2)	1290 (6)	1290 (6)	1293 (6)	1288 (4)
24	1257 (9)	1256 (7)	1264 (1)	1265 (1)	1271 (1)	1270 (1)	1262 (0)	1260 (0)	1267 (1)	1265 (1)
25	1218 (1)	1226 (0)	1248 (10)	1243 (3)	1231 (4)	1232 (2)	1252 (13)	1248 (9)	1227 (5)	1223 (4)
26	1189 (7)	1191 (3)	1191 (2)	1194 (1)	1213 (3)	1211 (2)	1205 (1)	1205 (0)	1197 (1)	1200 (0)
27	1167 (3)	1171 (2)	1187 (4)	1189 (5)	1174 (2)	1177 (1)	1188 (1)	1192 (0)	1167 (14)	1165 (7)
28	1152 (1)	1157 (1)	1164 (0)	1169 (0)	1149 (2)	1152 (0)	1159 (3)	1156 (0)	1162 (1)	1165 (7)
29	1129 (2)	1130 (0)	1138 (0)	1140 (0)	1148 (8)	1147 (8)	1141 (3)	1142 (1)	1142 (3)	1143 (3)
30	1099 (2)	1097 (1)	1108 (6)	1108 (4)	1092 (4)	1089 (2)	1112 (4)	1115 (3)	1111 (2)	1113 (1)
31	1060 (0)	1059 (0)	1059 (1)	1057 (0)	1036 (9)	1036 (5)	1036 (5)	1036 (4)	1059 (1)	1060 (0)
32	1039 (5)	1035 (3)	1031 (6)	1031 (3)	1019 (2)	1020 (1)	1024 (1)	1025 (1)	1029 (6)	1028 (3)
33	1031 (2)	1031 (1)	984 (2)	1008 (0)	988 (2)	999 (0)	986 (1)	1008 (0)	989 (2)	997 (0)

34	989 (2)	998 (0)	930 (11)	929 (6)	893 (12)	905 (3)	902 (7)	902 (5)	935 (4)	941 (0)
35	849 (7)	847 (5)	879 (16)	890 (1)	864 (10)	893 (9)	860 (24)	860 (20)	855 (7)	857 (6)
36	815 (1)	816 (1)	829 (1)	831 (1)	817 (1)	819 (1)	820 (1)	824 (0)	827 (1)	829 (1)
37	704 (1)	707 (4)	717 (4)	717 (4)	715 (4)	716 (4)	716 (3)	717 (3)	717 (5)	717 (4)
38	691 (1)	691 (1)	688 (2)	688 (2)	694 (1)	695 (1)	696 (2)	698 (2)	693 (1)	693 (1)
39	617 (4)	619 (4)	598 (4)	600 (3)	593 (7)	594 (7)	607 (8)	609 (7)	596 (3)	598 (2)
40	545 (0)	545 (0)	543 (2)	542 (1)	543 (1)	543 (1)	543 (0)	545 (0)	540 (0)	539 (0)
41	498 (1)	494 (0)	496 (2)	495 (2)	493 (1)	492 (1)	494 (2)	492 (1)	497 (2)	497 (2)
42	432 (2)	428 (1)	446 (3)	445 (3)	447 (3)	445 (3)	442 (1)	442 (1)	446 (2)	444 (2)
43	402 (1)	399 (1)	400 (0)	397 (0)	410 (1)	409 (1)	408 (0)	406 (0)	409 (1)	408 (1)
44	244 (1)	242 (1)	240 (1)	236 (1)	238 (1)	235 (1)	239 (1)	235 (1)	230 (0)	227 (2)
45	2851 (9)	2787 (10)	2841 (10)	2786 (14)	2807 (15)	2746 (20)	2813 (12)	2753 (17)	2836 (12)	2784 (15)
46	1212 (0)	1199 (0)	1178 (1)	1171 (0)	1165 (0)	1152 (0)	1165 (0)	1164 (2)	1184 (0)	1179 (0)
47	974 (0)	992 (0)	980 (0)	984 (0)	973 (0)	990 (2)	977 (0)	991 (0)	978 (0)	990 (2)
48	970 (0)	988 (0)	956 (0)	979 (3)	968 (0)	982 (0)	975 (1)	977 (0)	955 (4)	965 (2)
49	952 (1)	962 (1)	954 (4)	964 (1)	949 (4)	967 (2)	950 (4)	971 (2)	953 (1)	949 (1)
50	935 (2)	951 (1)	945 (0)	955 (0)	925 (13)	940 (13)	928 (15)	937 (17)	933 (4)	935 (2)
51	924 (0)	929 (1)	899 (10)	905 (3)	913 (2)	907 (0)	911 (4)	907 (1)	923 (6)	924 (7)
52	855 (1)	865 (1)	859 (0)	879 (14)	854 (1)	863 (8)	861 (1)	893 (1)	858 (2)	872 (4)
53	849 (0)	853 (1)	795 (1)	824 (0)	783 (3)	816 (0)	788 (0)	818 (2)	787 (2)	801 (0)
54	771 (20)	776 (18)	763 (41)	775 (5)	759 (37)	763 (44)	760 (62)	760 (49)	764 (14)	763 (4)
55	756 (58)	767 (21)	754 (22)	753 (54)	735 (28)	737 (38)	741 (9)	741 (30)	755 (44)	754 (52)
56	721 (60)	722 (74)	662 (60)	665 (42)	710 (41)	704 (13)	724 (19)	724 (10)	664 (64)	663 (55)
57	701 (0)	706 (1)	651 (1)	655 (5)	652 (3)	656 (3)	655 (24)	652 (22)	647 (5)	640 (7)
58	552 (0)	547 (0)	563 (1)	568 (1)	561 (5)	567 (5)	557 (1)	561 (0)	554 (0)	559 (0)
59	501 (0)	494 (0)	497 (1)	499 (2)	510 (0)	518 (0)	509 (4)	512 (4)	527 (0)	529 (0)
60	447 (0)	445 (1)	468 (1)	471 (1)	439 (11)	440 (12)	434 (1)	437 (2)	467 (3)	469 (3)
61	423 (4)	410 (3)	418 (6)	420 (6)	397 (0)	393 (0)	420 (5)	419 (5)	427 (6)	421 (6)
62	280 (2)	277 (1)	289 (1)	298 (0)	298 (1)	302 (1)	265 (0)	265 (0)	299 (0)	296 (0)
63	225 (0)	221 (0)	234 (1)	233 (1)	220 (1)	218 (1)	239 (1)	235 (1)	228 (1)	226 (0)
64	174 (9)	177 (8)	137 (0)	150 (0)	177 (0)	178 (1)	178 (2)	174 (1)	159 (2)	164 (2)
65	94 (1)	88 (1)	99 (2)	97 (2)	104 (0)	101 (0)	101 (1)	99 (1)	122 (0)	115 (0)
66	35 (2)	53 (0)	78 (1)	78 (1)	80 (2)	71 (1)	85 (0)	79 (0)	87 (0)	81 (0)

[a]Harmonic vibrational wavenumbers scaled according to 0.9510 x + 35.9 for wavenumbers > 2000 cm^{-1} and 0.9778 x + 6.3 for wavenumbers < 2000 cm^{-1}. [b]IR intensities in km mol^{-1} are listed in parentheses.

Table S9. Scaled harmonic and anharmonic vibrational wavenumbers and IR intensities of 4a-, 6a-, 10a-, and 10b-$C_{13}H_9N$ calculated with the B3LYP/6-311++G(d,p) method

ν_i	4a-$HC_{13}H_9N$		6a-$HC_{13}H_9N$		10a-$HC_{13}H_9N$		10b-$HC_{13}H_9N$	
	scaled[a]	anharmonic	scaled[a]	anharmonic	scaled[a]	anharmonic	scaled[a]	anharmonic
1	3073 (9)[b]	3101 (23)[b]	3074 (12)[b]	3113 (15)[b]	3077 (11)[b]	3105 (2)[b]	3076 (13)[b]	3079 (2)[b]
2	3071 (26)	3084 (1)	3072 (20)	3084 (23)	3074 (21)	3075 (3)	3073 (22)	3076 (11)
3	3066 (15)	3072 (26)	3066 (18)	3054 (4)	3069 (18)	3069 (8)	3066 (17)	3070 (39)
4	3064 (8)	3065 (6)	3057 (2)	3049 (8)	3065 (7)	3055 (21)	3061 (6)	3067 (30)
5	3049 (4)	3045 (3)	3053 (25)	3041 (16)	3055 (8)	3046 (6)	3056 (3)	3055 (2)
6	3045 (16)	3036 (17)	3048 (0)	3036 (1)	3046 (7)	3038 (5)	3050 (11)	3044 (6)
7	3041 (7)	3032 (2)	3044 (3)	3034 (3)	3043 (7)	3031 (8)	3041 (1)	3023 (2)
8	3040 (2)	2998 (3)	3035 (4)	3013 (18)	3035 (8)	3008 (10)	3040 (10)	3021 (23)
9	2970 (36)	2924 (27)	2980 (21)	2935 (19)	2989 (25)	2963 (16)	3011 (14)	3001 (14)
10	2678 (21)	2600 (15)	2657 (23)	2577 (15)	2665 (11)	2585 (11)	2688 (17)	2616 (0)
11	1650 (55)	1638 (7)	1645 (39)	1635 (8)	1606 (0)	1596 (0)	1601 (2)	1591 (0)
12	1600 (6)	1589 (2)	1598 (3)	1588 (2)	1586 (4)	1569 (0)	1598 (3)	1582 (1)
13	1578 (11)	1563 (3)	1576 (2)	1560 (0)	1573 (3)	1562 (1)	1578 (1)	1569 (1)
14	1557 (9)	1547 (2)	1556 (1)	1547 (0)	1508 (9)	1496 (1)	1509 (13)	1499 (5)
15	1494 (1)	1486 (0)	1494 (1)	1487 (0)	1481 (3)	1475 (0)	1479 (6)	1472 (1)
16	1465 (16)	1458 (8)	1460 (8)	1455 (3)	1467 (19)	1460 (8)	1472 (1)	1462 (1)
17	1457 (6)	1454 (3)	1454 (17)	1451 (10)	1453 (12)	1449 (0)	1448 (7)	1443 (0)
18	1395 (3)	1389 (0)	1399 (3)	1393 (0)	1403 (1)	1398 (0)	1410 (5)	1406 (0)
19	1378 (1)	1371 (0)	1380 (0)	1373 (0)	1383 (2)	1379 (1)	1394 (3)	1389 (1)
20	1366 (5)	1361 (2)	1358 (2)	1354 (1)	1345 (8)	1338 (3)	1330 (7)	1321 (3)
21	1315 (1)	1311 (0)	1313 (1)	1308 (0)	1309 (1)	1301 (0)	1310 (3)	1302 (0)
22	1314 (2)	1306 (1)	1306 (1)	1300 (1)	1298 (1)	1289 (0)	1284 (3)	1284 (0)
23	1291 (2)	1289 (1)	1289 (3)	1284 (2)	1283 (1)	1283 (0)	1260 (7)	1256 (3)
24	1252 (10)	1245 (4)	1249 (7)	1242 (1)	1251 (1)	1248 (2)	1250 (19)	1242 (5)
25	1231 (0)	1230 (1)	1228 (12)	1221 (3)	1221 (13)	1218 (5)	1223 (5)	1221 (3)
26	1217 (2)	1205 (10)	1199 (6)	1196 (3)	1198 (7)	1198 (2)	1215 (2)	1209 (1)
27	1202 (22)	1194 (6)	1180 (2)	1174 (0)	1189 (0)	1182 (0)	1192 (8)	1193 (0)
28	1163 (2)	1168 (2)	1161 (0)	1164 (0)	1174 (6)	1166 (0)	1179 (5)	1167 (3)
29	1153 (1)	1155 (0)	1154 (1)	1152 (2)	1162 (0)	1156 (2)	1164 (1)	1164 (0)
30	1124 (3)	1127 (2)	1117 (4)	1119 (4)	1158 (1)	1155 (1)	1162 (1)	1164 (1)
31	1119 (7)	1120 (4)	1110 (5)	1109 (3)	1113 (8)	1116 (6)	1117 (4)	1116 (2)
32	1051 (13)	1048 (7)	1054 (6)	1047 (5)	1105 (1)	1104 (0)	1092 (28)	1097 (11)
33	1038 (1)	1037 (0)	1043 (12)	1042 (8)	1044 (2)	1041 (1)	1043 (3)	1042 (2)

34	1015 (11)	1008 (7)	1033 (1)	1031 (1)	1034 (5)	1031 (3)	1027 (11)	1024 (7)
35	994 (4)	992 (3)	996 (4)	995 (1)	990 (8)	991 (5)	985 (8)	989 (0)
36	977 (0)	991 (0)	974 (0)	987 (0)	977 (0)	991 (0)	976 (0)	983 (4)
37	971 (5)	972 (5)	971 (4)	971 (1)	966 (1)	968 (1)	967 (1)	966 (1)
38	961 (10)	965 (2)	955 (1)	954 (4)	954 (2)	954 (4)	953 (1)	946 (2)
39	959 (2)	956 (0)	951 (4)	951 (1)	950 (10)	944 (2)	945 (3)	944 (0)
40	938 (2)	935 (4)	925 (6)	923 (8)	943 (1)	939 (4)	940 (3)	934 (4)
41	920 (2)	915 (2)	910 (4)	905 (1)	909 (3)	904 (3)	885 (9)	894 (3)
42	878 (12)	882 (12)	873 (2)	877 (3)	879 (4)	881 (4)	875 (8)	870 (8)
43	859 (9)	862 (4)	847 (6)	845 (6)	845 (8)	842 (6)	853 (6)	846 (8)
44	849 (5)	842 (5)	817 (7)	815 (4)	823 (6)	820 (3)	829 (2)	822 (2)
45	784 (4)	791 (4)	789 (2)	795 (2)	809 (0)	809 (0)	798 (11)	802 (7)
46	769 (12)	763 (5)	765 (7)	768 (10)	777 (2)	773 (0)	781 (2)	774 (4)
47	754 (31)	762 (6)	756 (53)	751 (45)	763 (52)	767 (35)	750 (41)	752 (29)
48	737 (23)	737 (36)	717 (19)	721 (8)	718 (4)	718 (8)	723 (24)	720 (13)
49	703 (28)	707 (3)	705 (11)	709 (2)	715 (50)	714 (24)	709 (31)	713 (14)
50	687 (35)	684 (40)	680 (55)	677 (49)	682 (32)	682 (39)	693 (40)	687 (36)
51	656 (4)	654 (11)	646 (3)	644 (10)	658 (1)	653 (9)	665 (1)	663 (3)
52	602 (1)	602 (1)	606 (1)	606 (1)	598 (3)	598 (2)	590 (6)	590 (4)
53	560 (5)	559 (5)	571 (0)	570 (0)	576 (1)	574 (1)	568 (3)	565 (3)
54	533 (2)	531 (2)	540 (1)	539 (1)	539 (3)	538 (2)	536 (1)	535 (0)
55	525 (5)	525 (4)	528 (1)	528 (1)	515 (4)	514 (3)	519 (1)	518 (1)
56	501 (3)	498 (3)	504 (1)	502 (1)	489 (0)	490 (0)	490 (3)	490 (3)
57	467 (2)	465 (1)	468 (1)	465 (1)	481 (2)	478 (2)	474 (5)	472 (5)
58	432 (2)	430 (2)	436 (5)	434 (5)	433 (4)	428 (2)	445 (5)	441 (5)
59	408 (1)	406 (1)	422 (5)	420 (5)	416 (5)	413 (5)	413 (4)	409 (3)
60	379 (1)	378 (1)	374 (1)	372 (1)	398 (3)	393 (2)	392 (0)	388 (0)
61	373 (3)	369 (4)	363 (0)	360 (0)	374 (0)	372 (0)	375 (3)	372 (3)
62	255 (0)	252 (0)	259 (1)	253 (1)	248 (0)	244 (0)	260 (1)	255 (0)
63	214 (1)	210 (1)	208 (1)	205 (1)	212 (0)	208 (0)	210 (0)	206 (0)
64	180 (0)	178 (0)	187 (2)	183 (1)	209 (2)	206 (3)	206 (1)	203 (1)
65	95 (1)	93 (1)	95 (0)	91 (0)	95 (0)	90 (0)	100 (1)	95 (1)
66	78 (0)	70 (0)	76 (1)	70 (1)	64 (1)	59 (1)	63 (0)	55 (0)

[a]Harmonic vibrational wavenumbers scaled according to $0.9510\,x + 35.9$ for wavenumbers > 2000 cm^{-1} and $0.9778\,x + 6.3$ for wavenumbers < 2000 cm^{-1}. [b] IR intensities in km mol^{-1} are listed in parentheses.

Table S10. Integrated lines and estimated mixing ratios of each species in the UV/IR-irradiated $C_{13}H_9N/Cl_2/p\text{-}H_2$ matrix experiments

group	assignments	mixing ratio / ppm	lines integrated / cm^{-1}
parent	$C_{13}H_9N$	132 ± 7	1492.7, 1239.9, 889.7, 768.1, and 720.5
A	$C_{13}H_9NH$	2.7 ± 0.4	1508.2, 1473.4, 1306.0, 744.6, and 709.4
B	9-$HC_{13}H_9N$	2.2 ± 0.1	1476.8, 1249.7, 759.5, and 651.1
C	1-$HC_{13}H_9N$	2.3 ± 0.2	1232.0, 956.3, 677.1, and 654.9
D	2-$HC_{13}H_9N$	2.3 ± 0.5	1431.4, 802.4, 737.8, and 659.8
E	10-$HC_{13}H_9N$	1.9 ± 1.0	1511.3 and 754.8
F	7-$HC_{13}H_9N$	2.3 ± 0.3	1489.0, 897.5, and 761.7
G	3-$HC_{13}H_9N$[a]	1.6 ± 0.6	1218.6 and 732.3
H	6-$HC_{13}H_9N$[a]	1.4	757.3

[a] Groups are tentatively assigned.

Table S11. Vertical excitation wavelengths and oscillator strengths of electronic excitation of $C_{13}H_9N$, 1-, 2-, 3-, 4-H$C_{13}H_9N$, $C_{13}H_9NH$, 6-, 7-, 8-, 9-, and 10-H$C_{13}H_9N$ predicted with the TD-B3LYP/6-311++G(d,p) method

State	$C_{13}H_9N$			1-H$C_{13}H_9N$			2-H$C_{13}H_9N$			3-H$C_{13}H_9N$		
	λ / nm	*f*	Assignment	λ / nm	*f*	Assignment	λ / nm	*f*	Assignment	λ / nm	*f*	Assignment
1	311	0.01	HOMO−1 → LUMO	577	0.00	HOMO(α) → LUMO(α)	568	0.00	HOMO(α) → LUMO(α)	620	0.00	HOMO(α) → LUMO(α)
2	294	0.05	HOMO → LUMO	454	0.00	HOMO(α) → LUMO+1(α)	500	0.04	HOMO(α) → LUMO+1(α)	438	0.02	HOMO(β) → LUMO(β)
3	292	0.00	HOMO−2 → LUMO	396	0.05	HOMO(α) → LUMO+1(α), HOMO(β) → LUMO+1(β)	405	0.03	HOMO(β) → LUMO(β)	419	0.01	HOMO(α) → LUMO+1(α)
4	263	0.12	HOMO → LUMO+1	379	0.11	HOMO(β) → LUMO(β)	387	0.00	HOMO−2(β) → LUMO(β)	389	0.00	HOMO(β) → LUMO+1(β)
5	258	0.00	HOMO−2 → LUMO+1	360	0.00	HOMO−1(β) → LUMO(β)	365	0.04	HOMO−1(β) → LUMO(β)	381	0.00	HOMO−1(β) → LUMO(β)
6	254	0.50	HOMO−1 → LUMO+1	334	0.03	HOMO(α) → LUMO+4(α)	351	0.07	HOMO−1(β) → LUMO(β)	369	0.02	HOMO(α) → LUMO+2(α)
7	241	0.01	HOMO → LUMO+2	329	0.00	HOMO(α) → LUMO+2(α)	336	0.00	HOMO(α) → LUMO+2(α)	333	0.00	HOMO(α) → LUMO+3(α)
8	237	0.15	HOMO−3 → LUMO	324	0.03	HOMO−2(β) → LUMO(β)	329	0.01	HOMO(α) → LUMO+5(α)	328	0.11	HOMO−2(β) → LUMO(β)
9	221	0.00	HOMO → LUMO+3	321	0.00	HOMO−2(α) → LUMO(α)	304	0.00	HOMO(α) → LUMO+3(α)	321	0.00	HOMO−2(α) → LUMO(α)
10	219	0.00	HOMO−2 → LUMO+2	301	0.00	HOMO−3(α) → LUMO(α)	303	0.00	HOMO−2(α) → LUMO+1(α)	307	0.02	HOMO−3(β) → LUMO(β)
11	218	0.00	HOMO−1 → LUMO+3	296	0.00	HOMO(α) → LUMO+3(α)	300	0.00	HOMO(α) → LUMO+7(α)	307	0.00	HOMO(α) → LUMO+4(α)
12	217	0.24	HOMO−4 → LUMO	292	0.00	HOMO−2(β) → LUMO+1(β)	294	0.00	HOMO(α) → LUMO+5(α), HOMO(β) → LUMO+2(β)	299	0.00	HOMO(β) → LUMO+2(β)

State	4-$HC_{13}H_9N$			$C_{13}H_9NH$			6-$HC_{13}H_9N$			7-$HC_{13}H_9N$		
	λ / nm	*f*	Assignment	λ / nm	*f*	Assignment	λ / nm	*f*	Assignment	λ / nm	*f*	Assignment
1	591	0.01	HOMO(α) → LUMO(α)	811	0.01	HOMO(α) → LUMO(α)	576	0.00	HOMO−1(β) → LUMO(β)	542	0.00	HOMO(α) → LUMO(α)
2	445	0.04	HOMO(α) → LUMO+1(α)	551	0.04	HOMO(α) → LUMO+1(α)	510	0.01	HOMO(β) → LUMO(β)	418	0.11	HOMO(β) → LUMO(β)
3	403	0.09	HOMO(β) → LUMO(β)	508	0.00	HOMO(α) → LUMO+2(α)	406	0.09	HOMO(α) → LUMO(α)	415	0.00	HOMO−2(β) → LUMO(β)
4	383	0.00	HOMO−1(α) → LUMO(α), HOMO(β) → LUMO+1(β)	412	0.01	HOMO(α) → LUMO+5(α)	387	0.01	HOMO−2(β) → LUMO(β)	408	0.02	HOMO−1(β) → LUMO(β)
5	364	0.00	HOMO−1(β) → LUMO(β)	406	0.00	HOMO(α) → LUMO+3(α)	372	0.02	HOMO(α) → LUMO+1(α), HOMO−3(β) → LUMO(β)	374	0.01	HOMO−1(α) → LUMO(α), HOMO(β) → LUMO+1(β)
6	342	0.01	HOMO(α) → LUMO+3(α)	402	0.00	HOMO(α) → LUMO+4(α)	349	0.02	HOMO−1(α) → LUMO(α), HOMO(β) → LUMO+1(β)	358	0.02	HOMO(α) → LUMO+1(α)
7	326	0.00	HOMO(α) → LUMO+2(α)	372	0.02	HOMO−1(β) → LUMO(β)	331	0.00	HOMO(α) → LUMO+1(α)	328	0.01	HOMO(α) → LUMO+3(α), HOMO−3(β) → LUMO(β)
8	323	0.05	HOMO−2(β) → LUMO(β)	360	0.01	HOMO(α) → LUMO+8(α)	307	0.00	HOMO−4(β) → LUMO(β)	312	0.00	HOMO(α) → LUMO+2(α)
9	308	0.00	HOMO−2(α) → LUMO(α)	353	0.00	HOMO(α) → LUMO+6(α)	297	0.00	HOMO−4(β) → LUMO(β)	299	0.00	HOMO−2(α) → LUMO(α)
10	300	0.00	HOMO(α) → LUMO+4(α)	348	0.00	HOMO(α) → LUMO+7(α)	279	0.00	HOMO(α) → LUMO+2(α)	291	0.00	HOMO−3(α) → LUMO(α)
11	296	0.01	HOMO(β) → LUMO+2(β)	345	0.09	HOMO(β) → LUMO(β)	278	0.03	HOMO(α) → LUMO+3(α)	289	0.08	HOMO(α) → LUMO+3(α)
12	292	0.00	HOMO(α) → LUMO+5(α)	324	0.02	HOMO−1(β) → LUMO(β)	276	0.00	HOMO−2(α) → LUMO(α)	285	0.01	HOMO−3(β) → LUMO(β)

State	8-$HC_{13}H_9N$			9-$HC_{13}H_9N$			10-$HC_{13}H_9N$		
	λ / nm	*f*	Assignment	λ / nm	*f*	Assignment	λ / nm	*f*	Assignment
1	624	0.01	HOMO(α) → LUMO(α)	548	0.00	HOMO(β) → LUMO(β)	564	0.01	HOMO(α) → LUMO(α)
2	423	0.03	HOMO(β) → LUMO(β)	467	0.02	HOMO−1(β) → LUMO(β)	428	0.02	HOMO(β) → LUMO(β)
3	406	0.01	HOMO(α) → LUMO+1(α)	464	0.00	HOMO−2(β) → LUMO(β)	401	0.01	HOMO(α) → LUMO+1(α)
4	396	0.00	HOMO−1(α) → LUMO(α)	420	0.03	HOMO(α) → LUMO(α)	376	0.01	HOMO−1(α) → LUMO(α),<br>HOMO(β) → LUMO(β),<br>HOMO(β) → LUMO+1(β)
5	372	0.00	HOMO(α) → LUMO+3(α)	376	0.11	HOMO(α) → LUMO+1(α)	351	0.15	HOMO−1(β) → LUMO(β)
6	361	0.00	HOMO−2(β) → LUMO(β)	359	0.01	HOMO−2(α) → LUMO(α),<br>HOMO(β) → LUMO+1(β)	351	0.00	HOMO−2(β) → LUMO(β)
7	335	0.00	HOMO(α) → LUMO+2(α)	331	0.00	HOMO−3(β) → LUMO(β)	326	0.00	HOMO−3(α) → LUMO(α)
8	333	0.00	HOMO−2(α) → LUMO(α)	314	0.00	HOMO(α) → LUMO+2(α)	323	0.00	HOMO(α) → LUMO+2(α)
9	332	0.08	HOMO−1(β) → LUMO(β)	299	0.00	HOMO−3(β) → LUMO(β)	323	0.03	HOMO(α) → LUMO+4(α)
10	311	0.03	HOMO−1(α) → LUMO(α),<br>HOMO(β) → LUMO+1(β)	291	0.00	HOMO−1(α) → LUMO+1(α)	299	0.00	HOMO(α) → LUMO+4(α)
11	299	0.00	HOMO(α) → LUMO+4(α)	287	0.00	HOMO−4(β) → LUMO(β)	290	0.00	HOMO(α) → LUMO+3(α)
12	298	0.00	HOMO(β) → LUMO+2(β)	280	0.00	HOMO(α) → LUMO+3(α)	287	0.00	HOMO(α) → LUMO+10(α)

(a) 1-$H^+C_{13}H_9N$; 155 kJ mol^{-1}

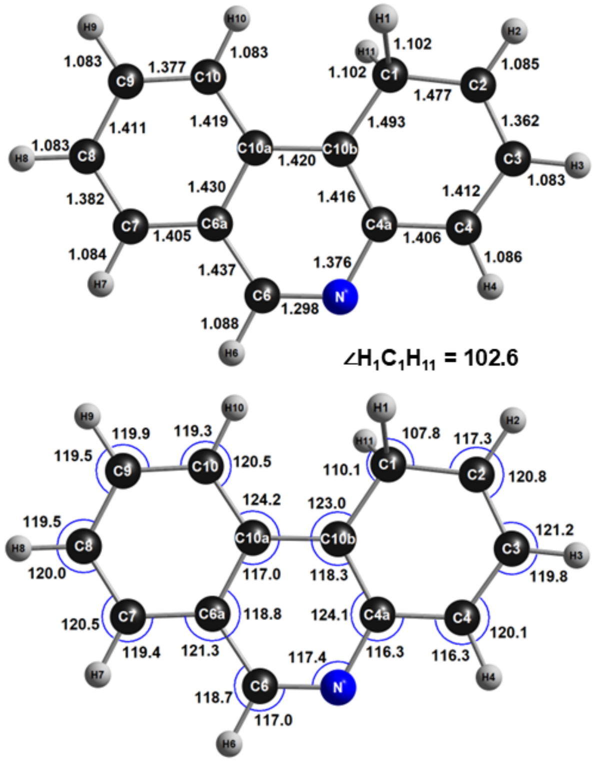

(b) 2-H^+C13H9N; 158 kJ mol^{-1}

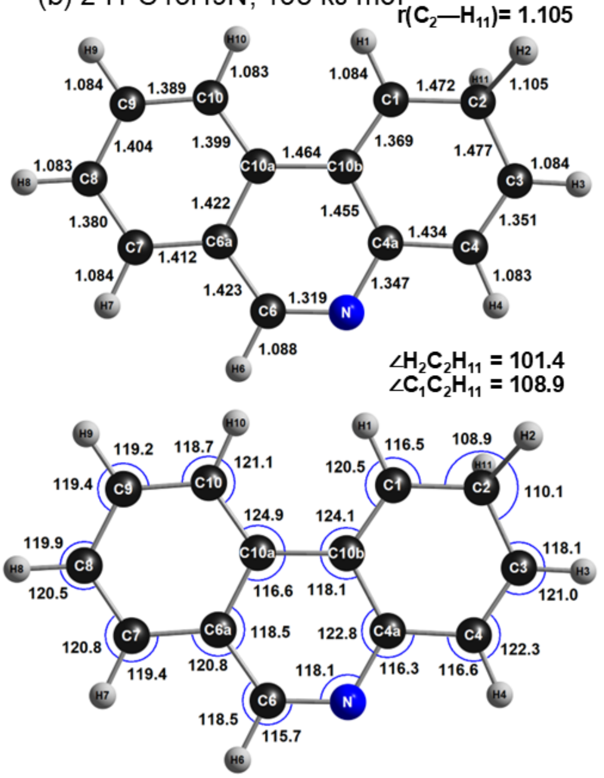

(c) 3-$H^+C_{13}H_9N$; 160 kJ mol^{-1}

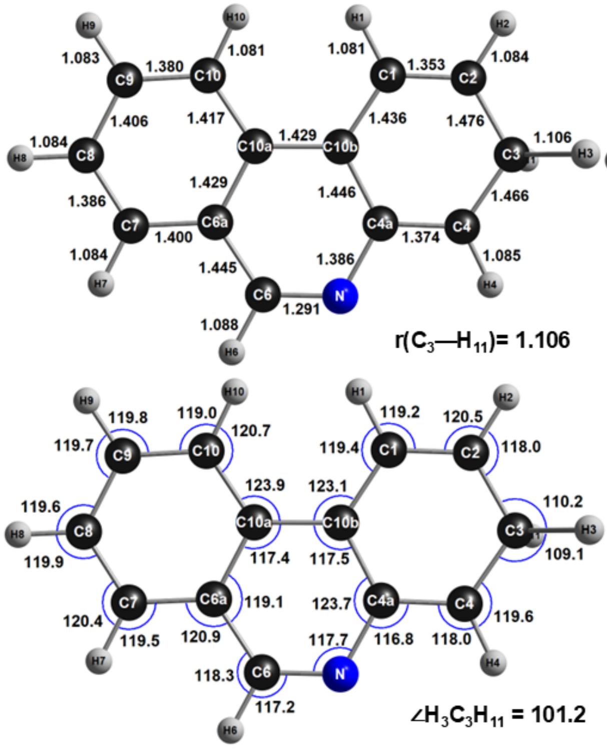

(d) 4-$H^+C_{13}H_9N$; 150 kJ mol^{-1}

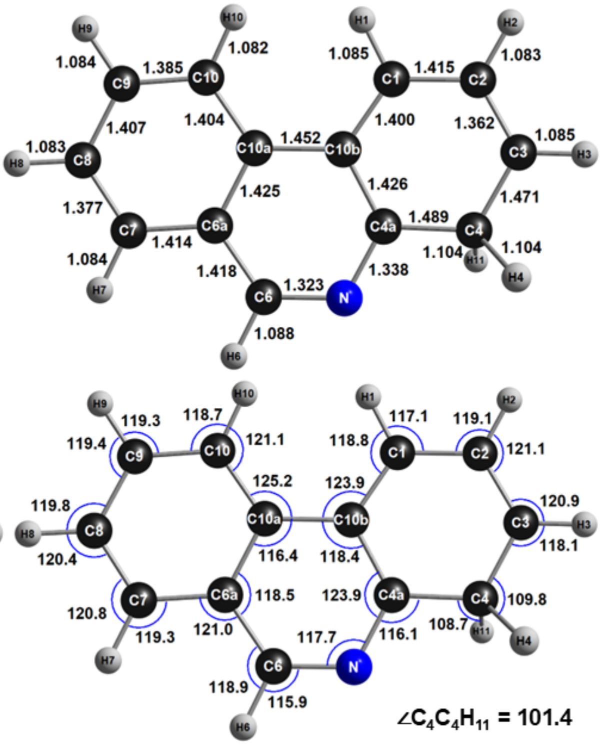

(e) $C_{13}H_9NH^+$; 0 kJ mol^{-1}

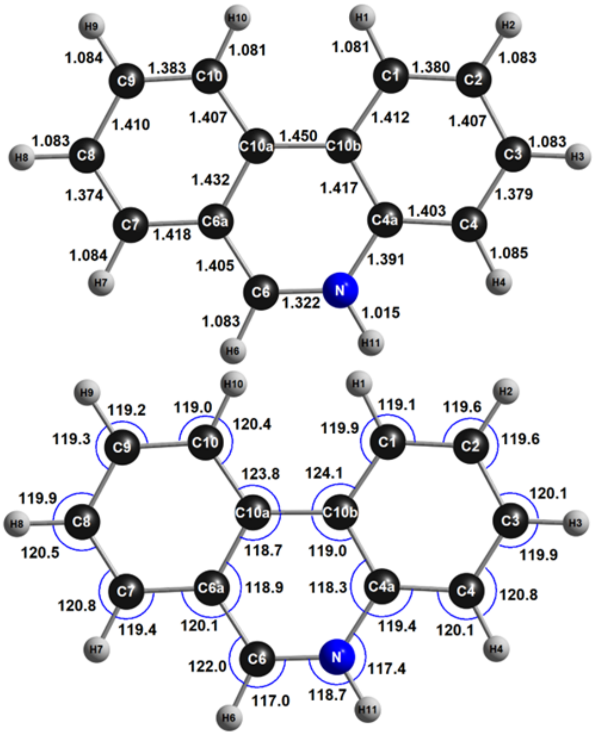

(f) 6-$H^+C_{13}H_9N$; 197 kJ mol^{-1}

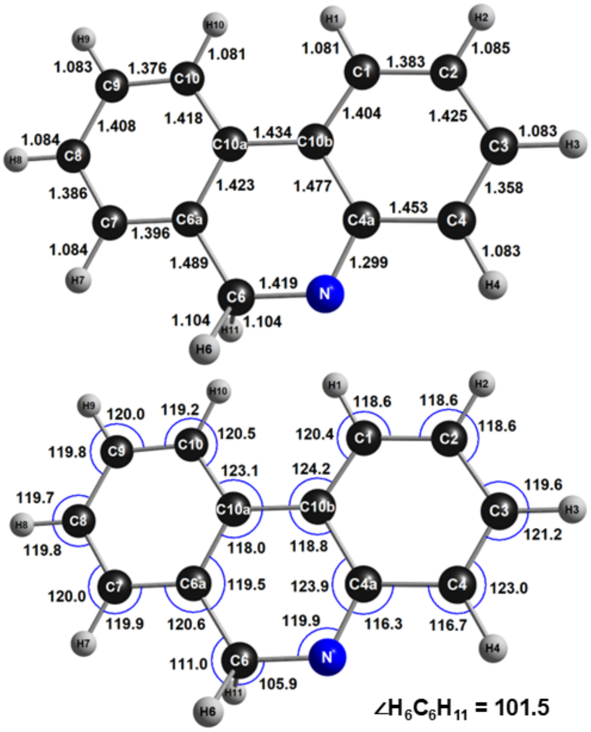

(g) 7-$H^+C_{13}H_9N$; 186 kJ mol^{-1}

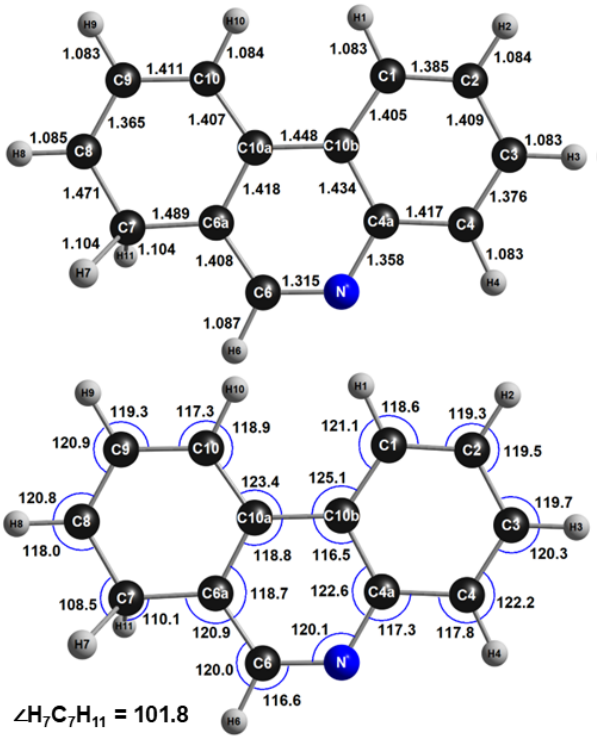

(h) 8-$H^+C_{13}H_9N$; 168 kJ mol^{-1}

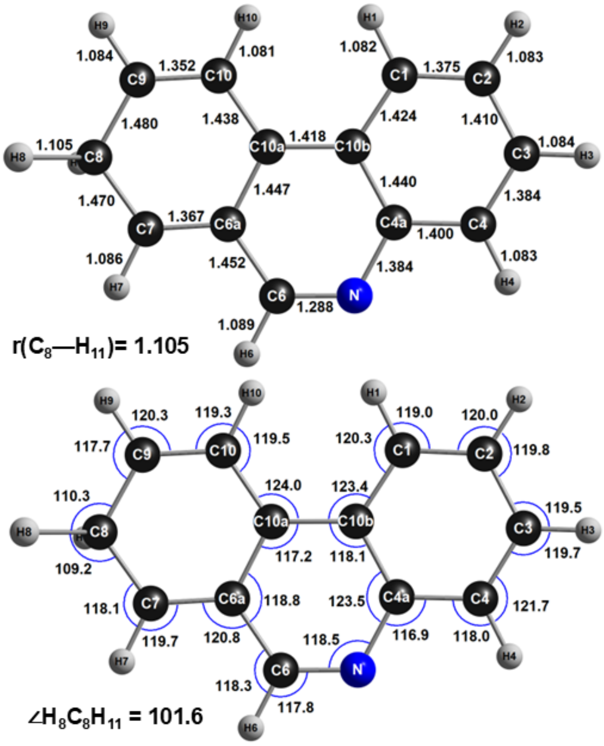

(i) 9-$H^+C_{13}H_9N$; 194 kJ mol^{-1}

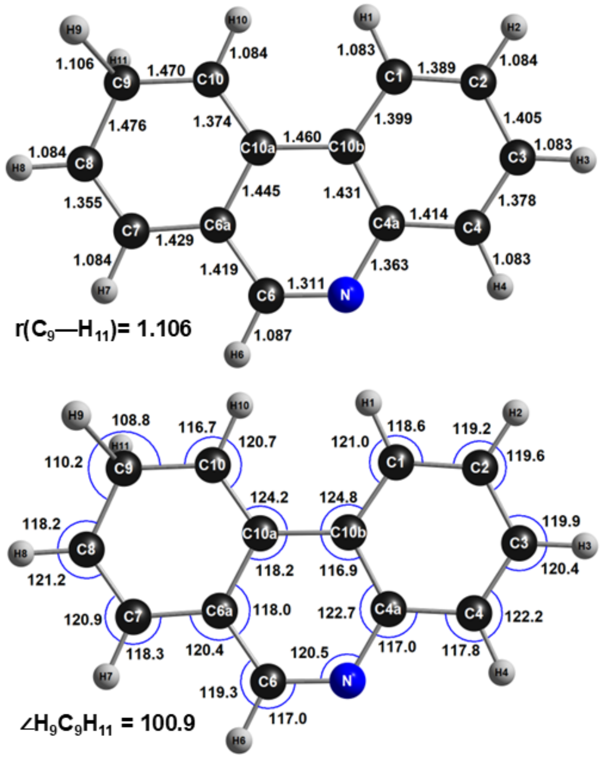

(j) 10-$H^+C_{13}H_9N$; 164 kJ mol^{-1}

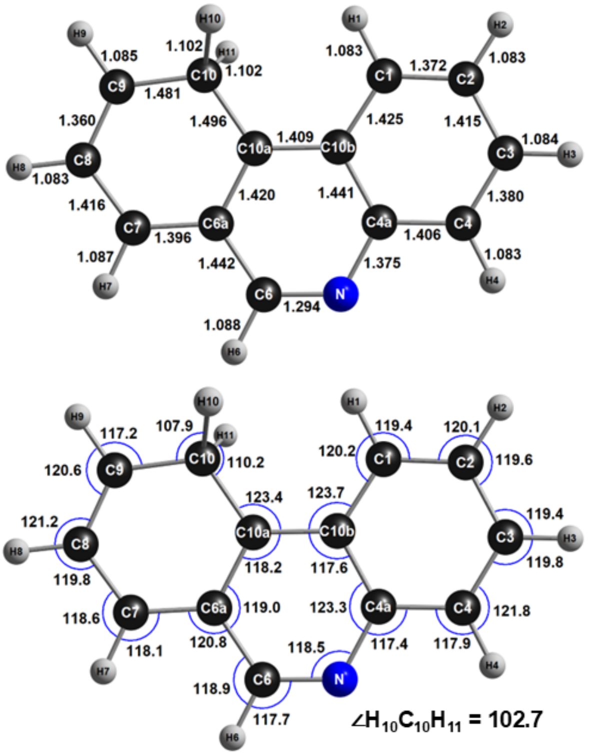

(k) 4a-$H^+C_{13}H_9N$; 214 kJ mol^{-1}

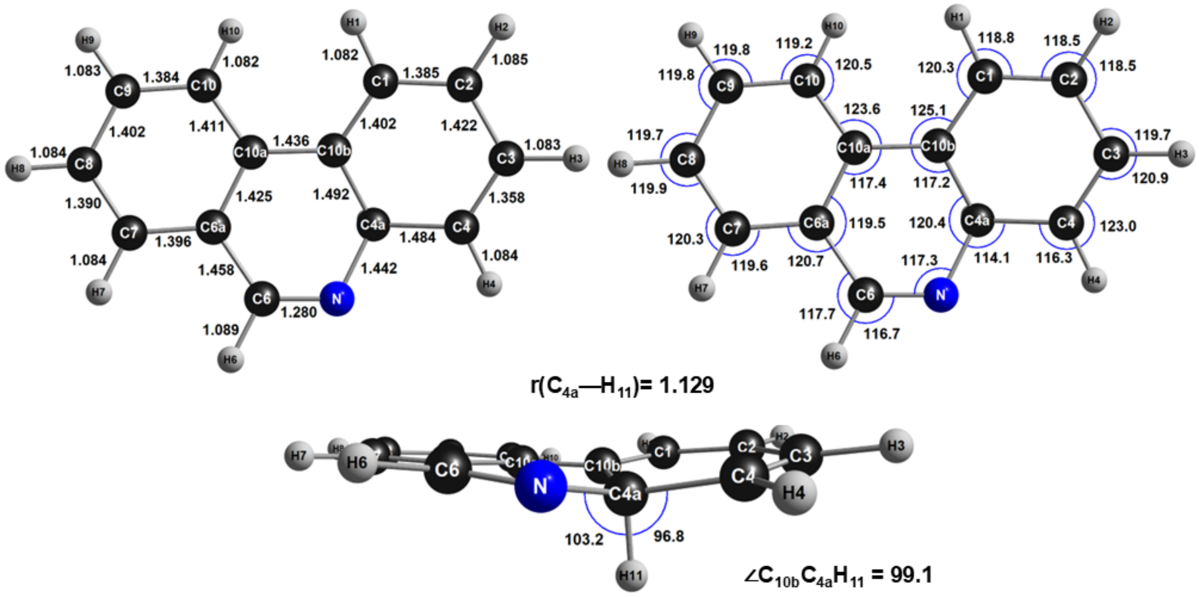

(l) 6a-H$^+$C$_{13}$H$_9$N; 215 kJ mol^{-1}

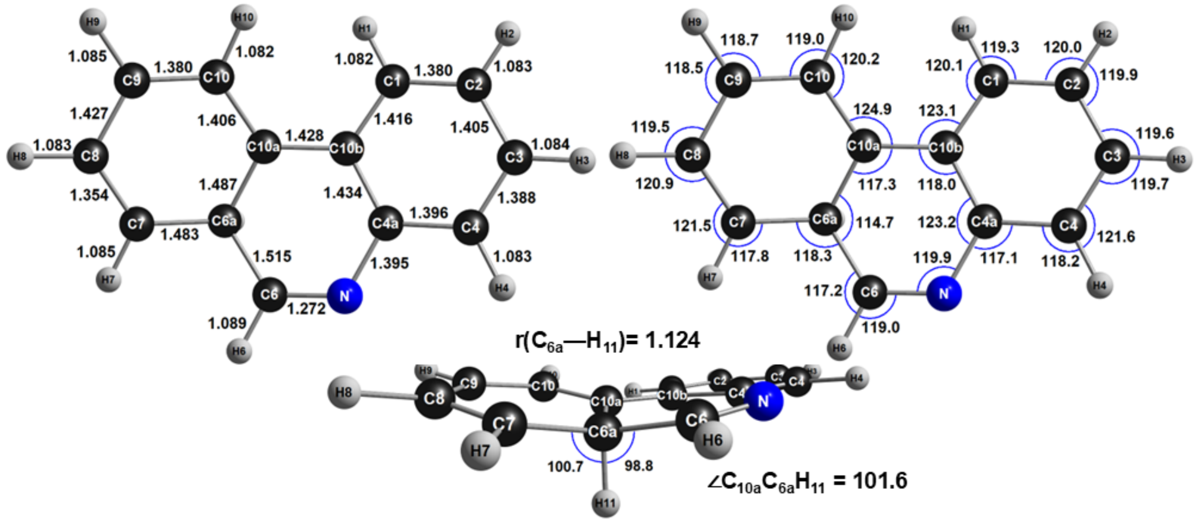

(m) 10a-H$^+$C$_{13}$H$_9$N; 237 kJ mol^{-1}

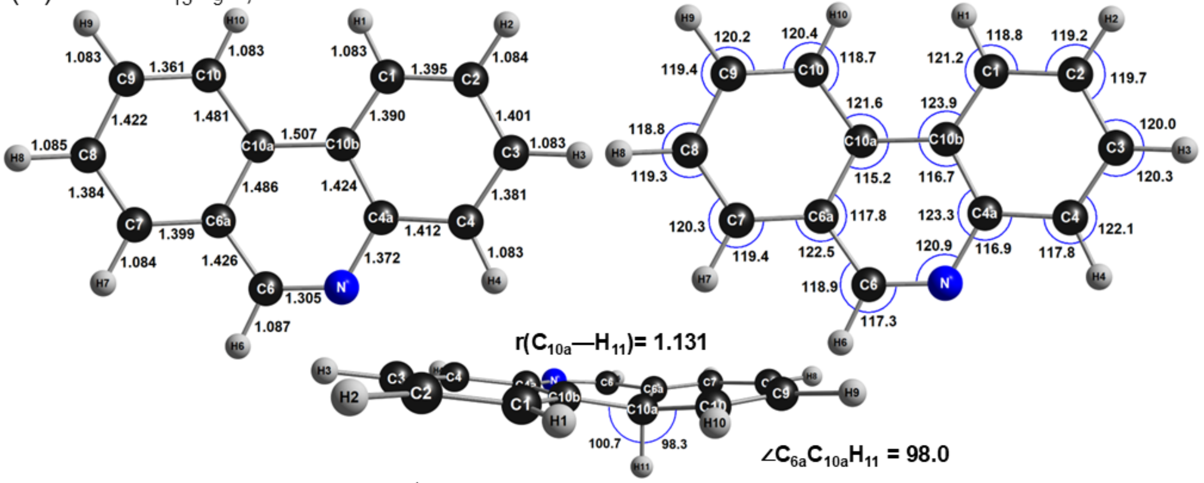

(n) 10b-H$^+$C$_{13}$H$_9$N; 202 kJ mol^{-1}

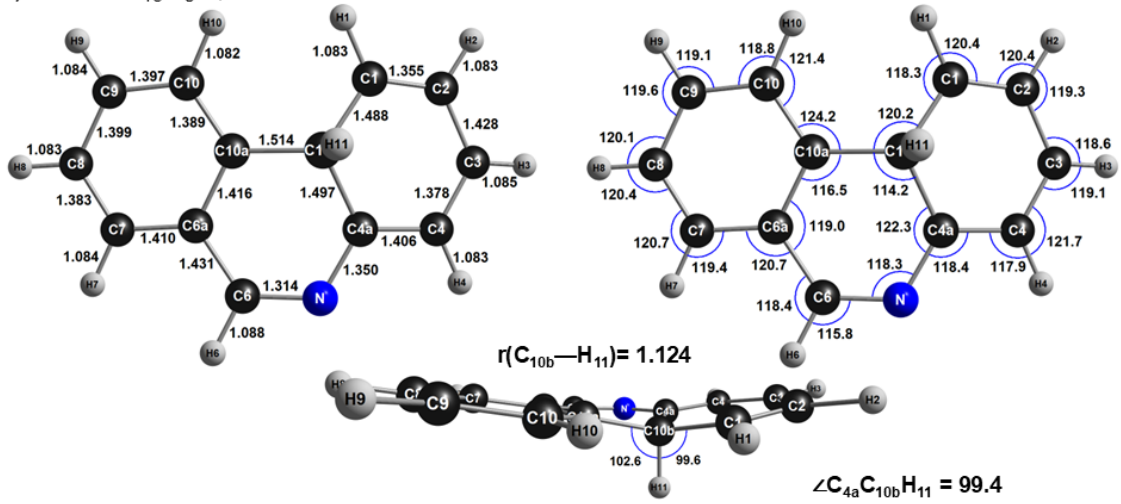

Figure S1. Geometries and relative energies of isomers of protonated phenanthridine (H$^+$C$_{13}$H$_9$N). Energies were calculated with the CCSD(T)/6-311++G(d,p)//B3LYP/6-311++G(d,p) method and corrected for zero-point vibrational energy (ZPVE) calculated with the B3LYP/6-311++G(d,p) method. Bond lengths are in Å and bond angles in degree.

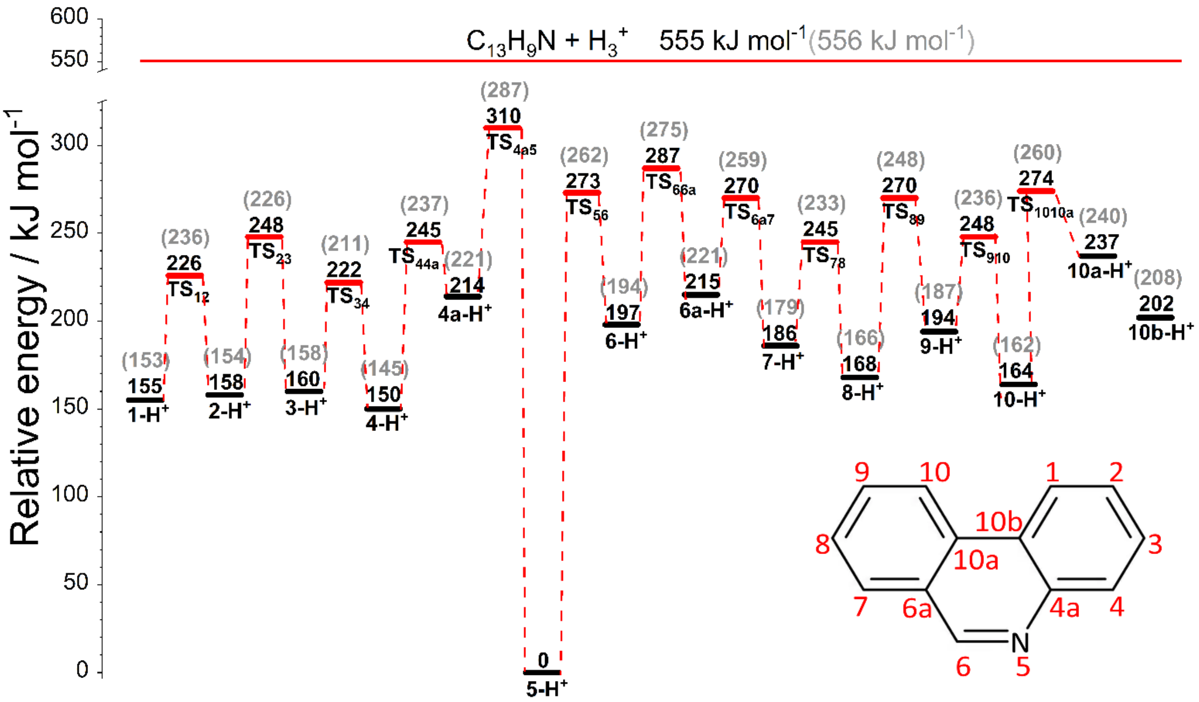

Figure S2. Potential-energy scheme for the isomerization of various isomers of protonated phenanthridine ($H^+C_{13}H_9N$, indicated as *n*-H^+). The isomers are indicated by the site-number *n* of protonation (see lower-right inset for the numbering). Transition states for isomerization are in red. The energies relative to the most stable isomer $C_{13}H_9NH^+$ (5-H^+), calculated with the CCSD(T)/6-311++G(d,p)//B3LYP/6-311++G(d,p) method, are in black; values calculated with the B3LYP/6-311++G(d,p) method are in parentheses (gray). Zero-point vibrational energy (ZPVE) obtained from the B3LYP/6-311++G(d,p) method were corrected. The energy of $C_{13}H_9N + H_3^+$ is 555 or 556 kJ mol^{-1} above $C_{13}H_9NH + H_2$ according to CCSD(T) or B3LYP calculations, respectively.

(a) 1-$HC_{13}H_9N$; 26 kJ mol^{-1}

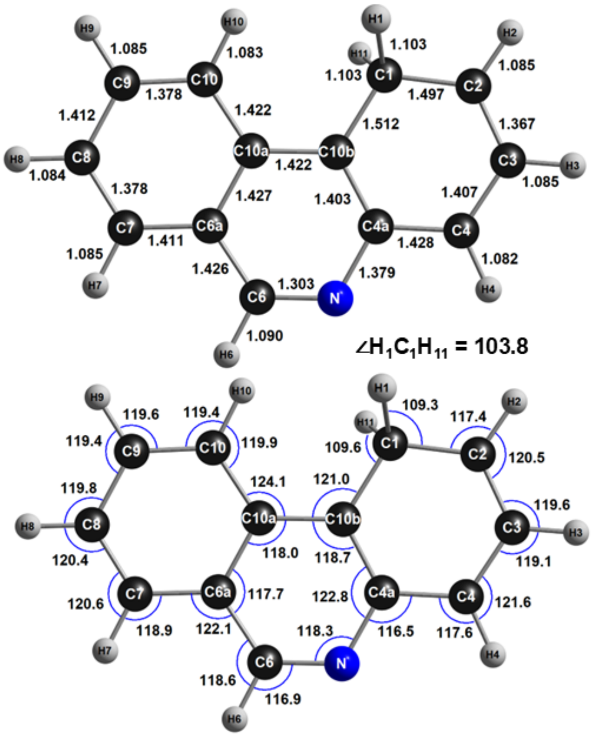

(b) 2-HC13H9N; 39 kJ mol^{-1}

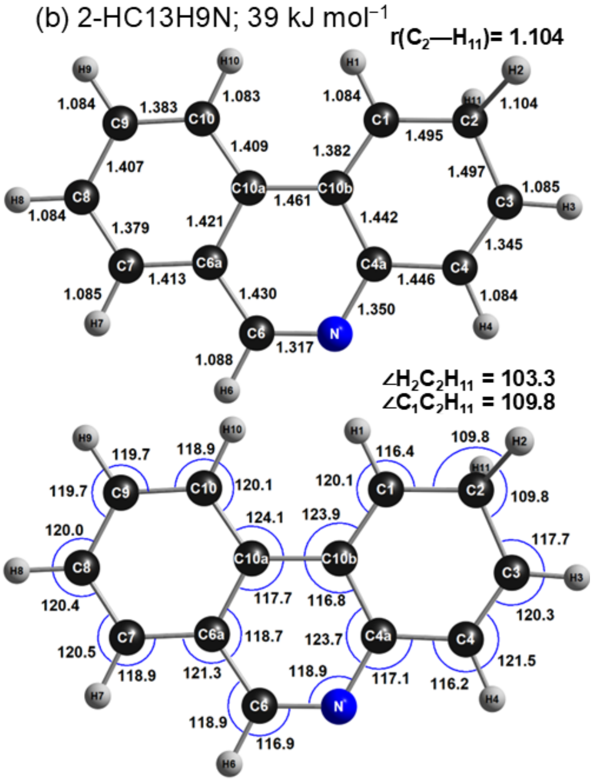

(c) 3-$HC_{13}H_9N$; 45 kJ mol^{-1}

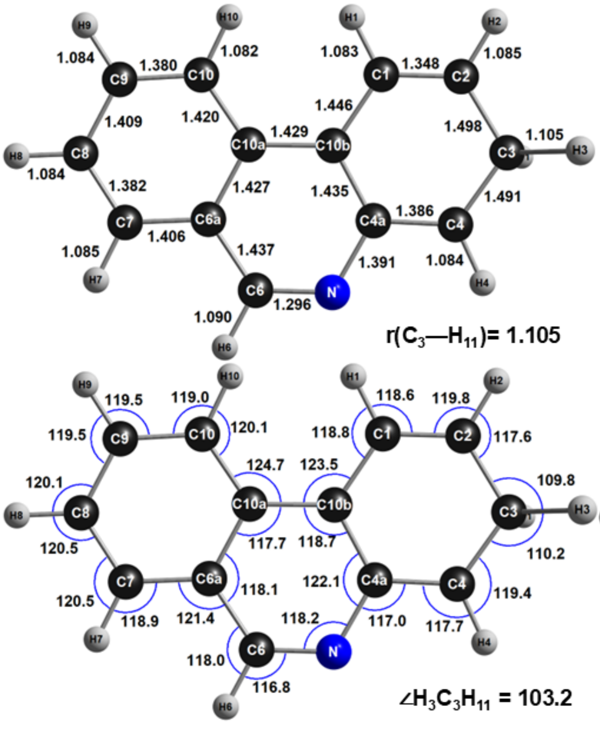

(d) 4-$HC_{13}H_9N$; 27 kJ mol^{-1}

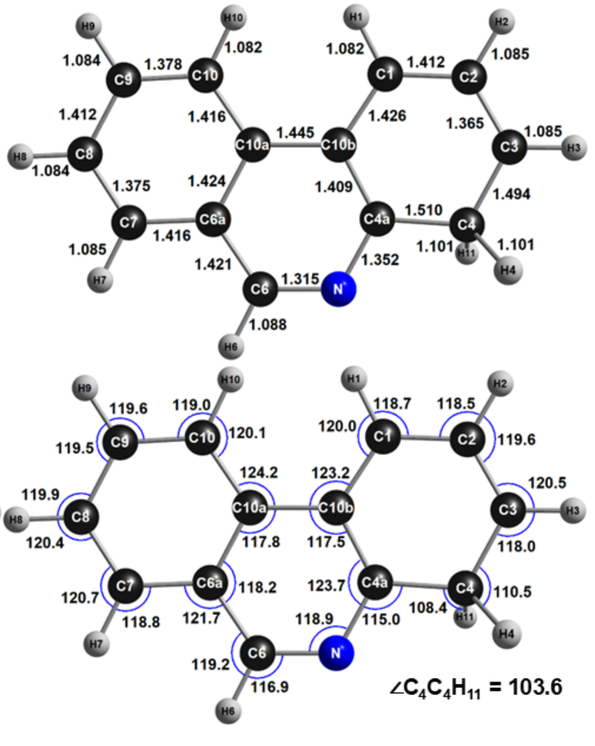

(e) $C_{13}H_9NH$; 0 kJ mol^{-1}

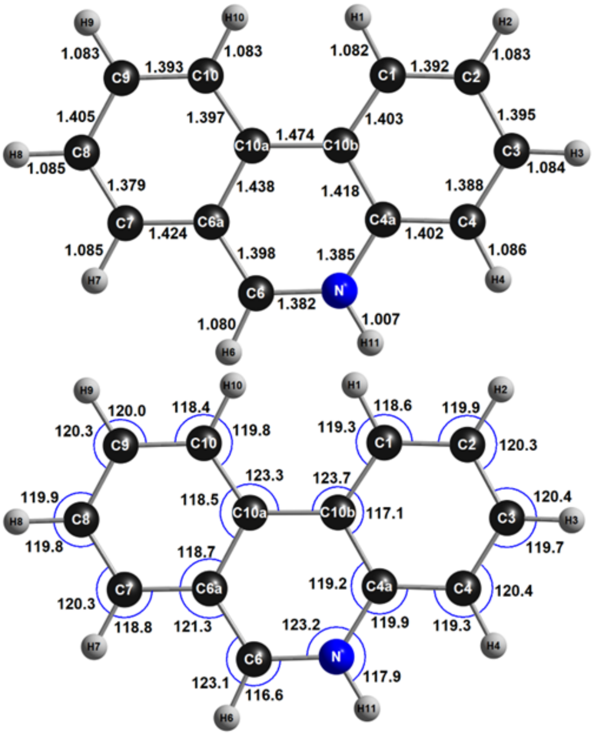

(f) 6-$HC_{13}H_9N$; 28 kJ mol^{-1}

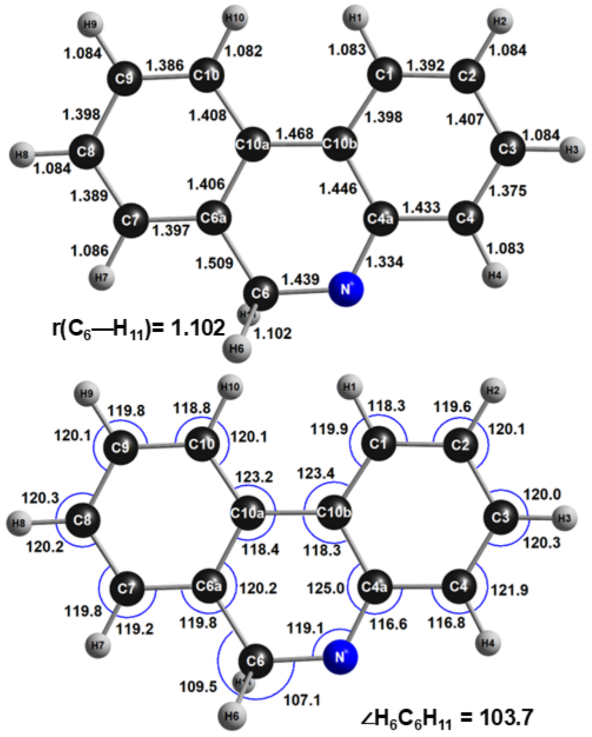

(g) 7-$HC_{13}H_9N$; 29 kJ mol^{-1}

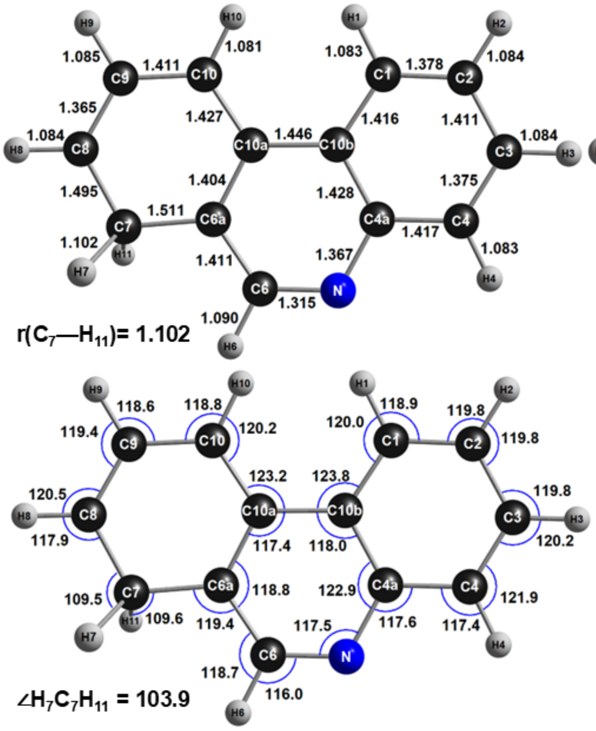

(h) 8-$HC_{13}H_9N$; 44 kJ mol^{-1}

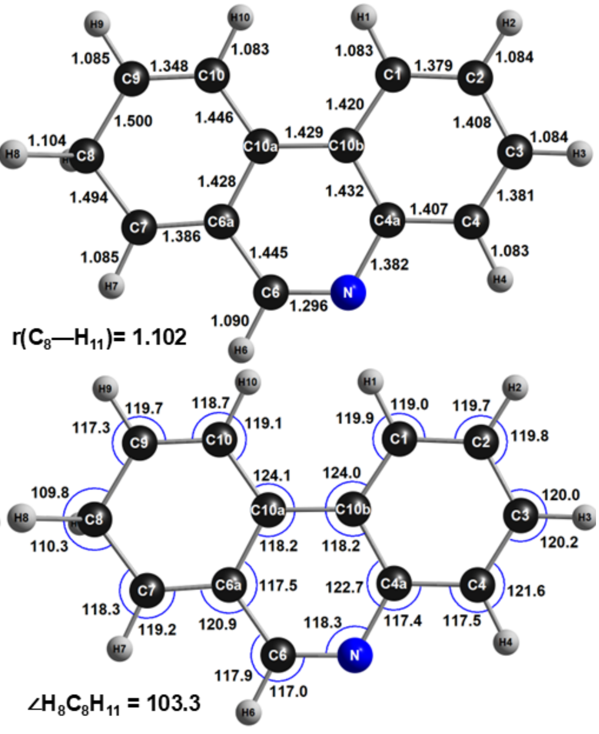

(i) 9-$HC_{13}H_9N$; 41 kJ mol^{-1}

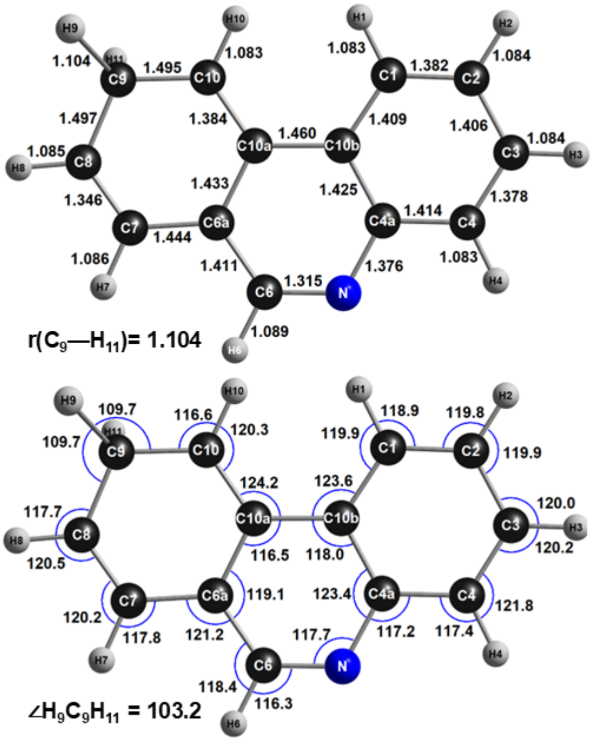

(j) 10-$HC_{13}H_9N$; 25 kJ mol^{-1}

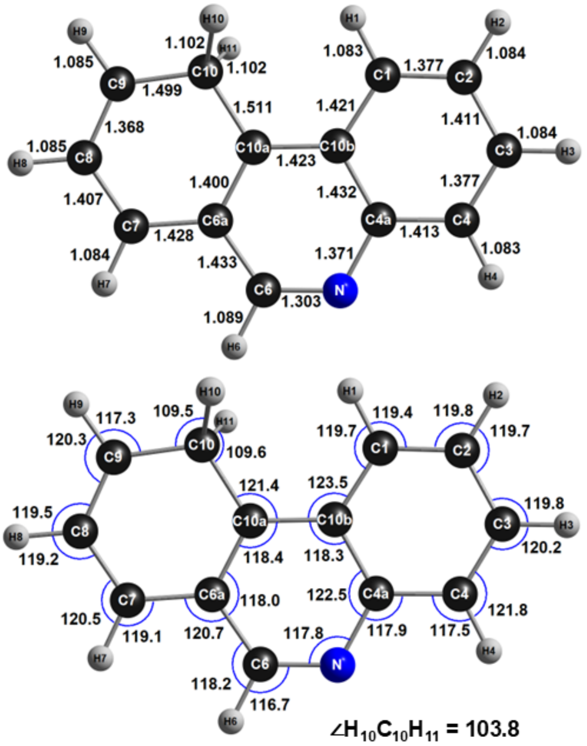

(k) 4a-$HC_{13}H_9N$; 87 kJ mol^{-1}

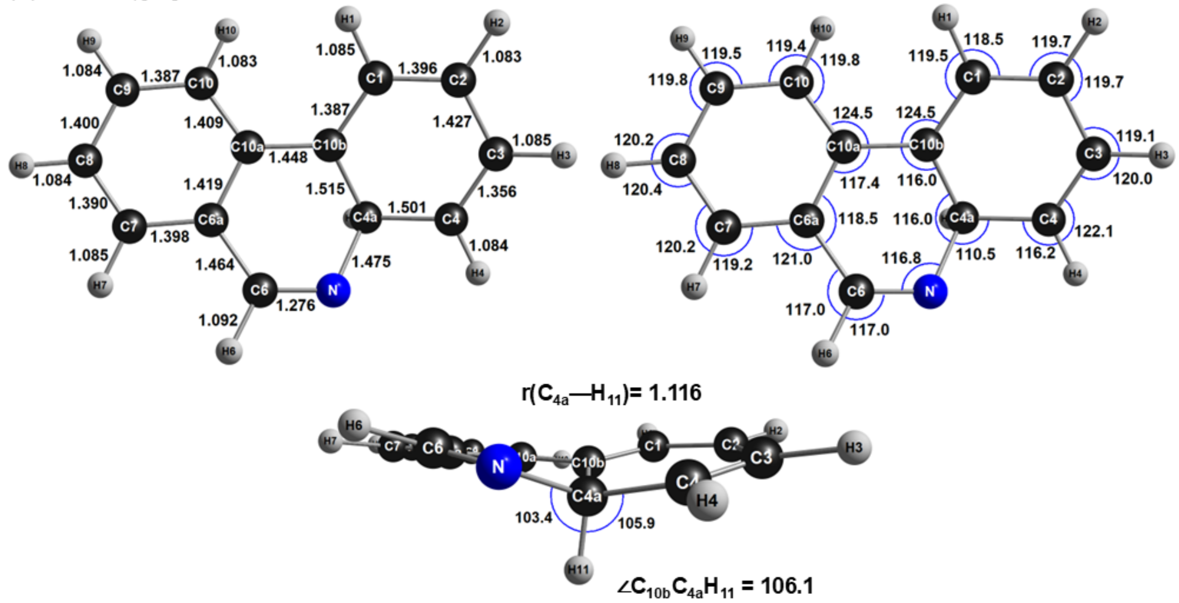

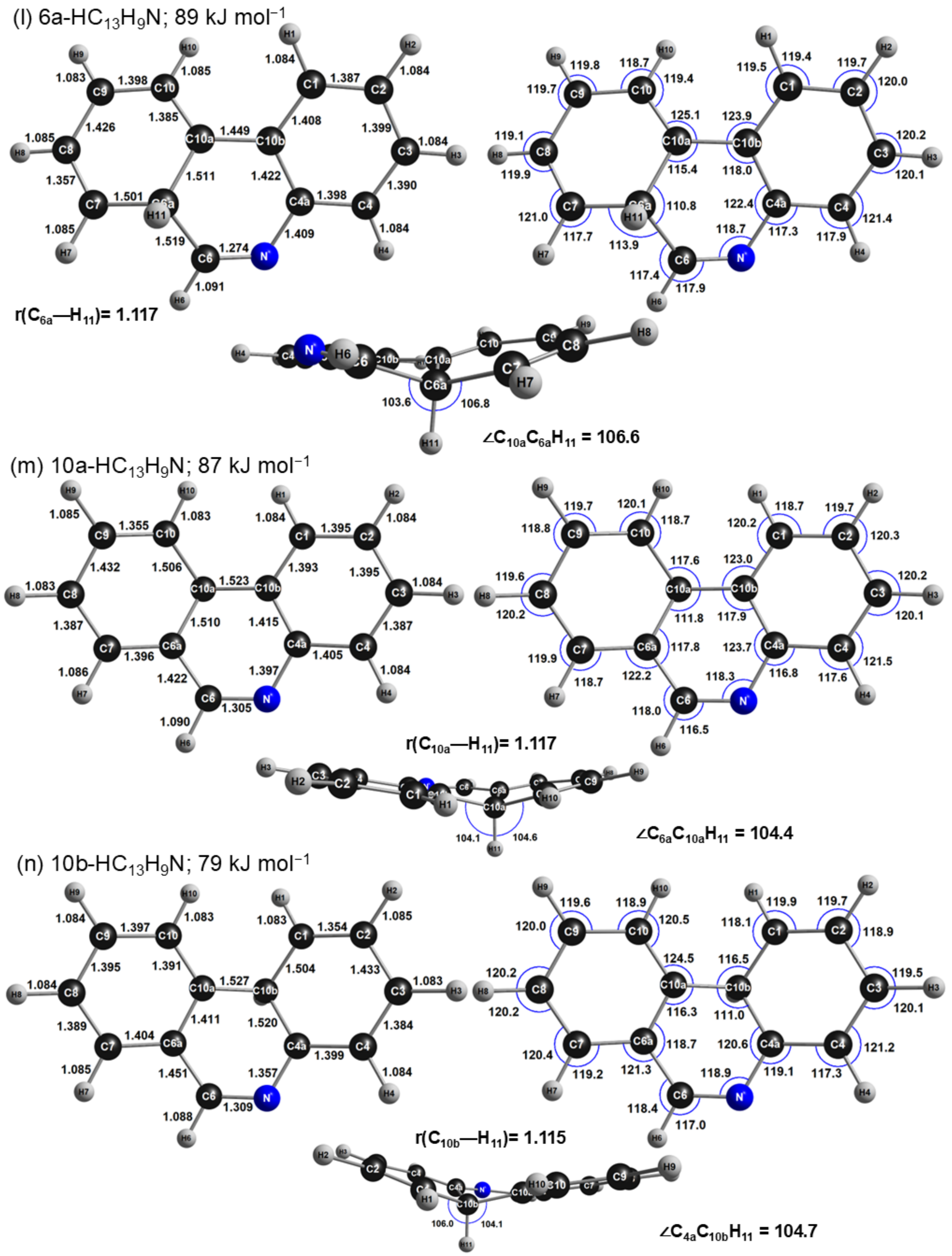

Figure S3. Geometries and relative energies of isomers of hydrogenated phenanthridine ($HC_{13}H_9N$). Energies were calculated with the CCSD(T)/6-311++G(d,p)//B3LYP/6-311++G(d,p) method and corrected for zero-point vibrational energy (ZPVE) calculated with the B3LYP/6-311++G(d,p) method. Bond lengths are in Å and bond angles in degree.

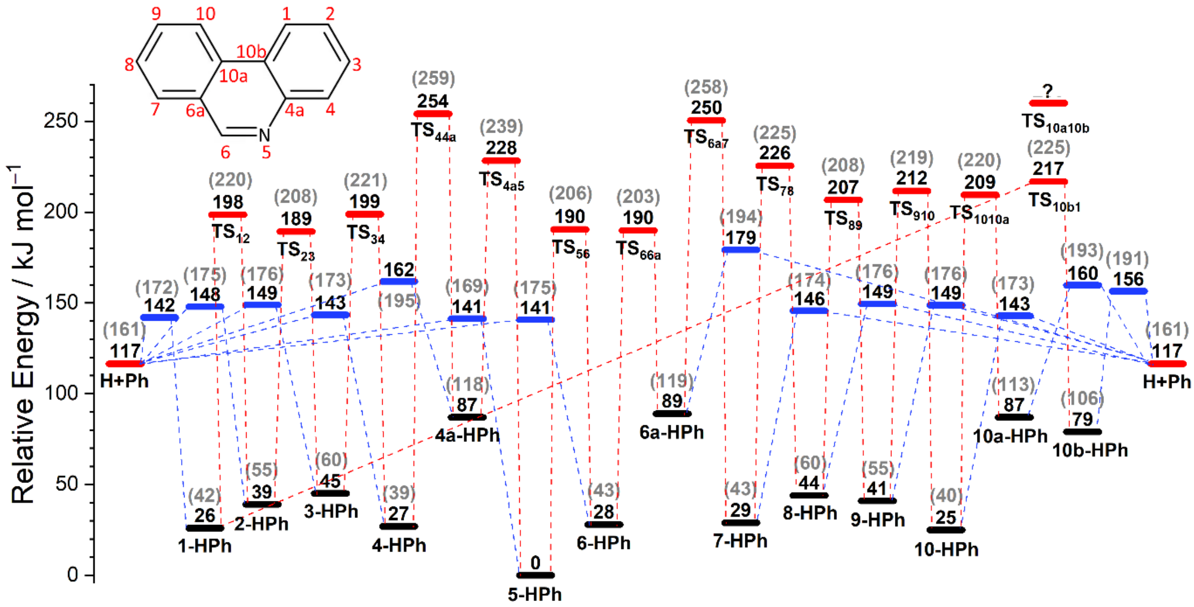

Figure S4. Potential-energy scheme for the formation and isomerization of various isomers of hydrogenated phenanthridine ($HC_{13}H_9N$, indicated as *n*-HPh). The isomers are indicated by the site-number *n* of hydrogenation (see upper-left inset for the numbering). Transition states for isomerization are in red and those for the formation from H + $C_{13}H_9N$ (indicated as H + Ph) are in blue. The energies relative to the most stable isomer $C_{13}H_9NH$ (5-HPh), calculated with the CCSD(T)/6-311++G(d,p)//B3LYP/6-311++G(d,p) method, are in black; values calculated with the B3LYP/6-311++G(d,p) method are in parentheses (gray). Zero-point vibrational eergy (ZPVE) obtained from the B3LYP/6-311++G(d,p) method were corrected. The energy of H + $C_{13}H_9N$ is 117 or 161 kJ mol^{-1} above $C_{13}H_9NH$ according to CCSD(T) or B3LYP calculations, respectively.

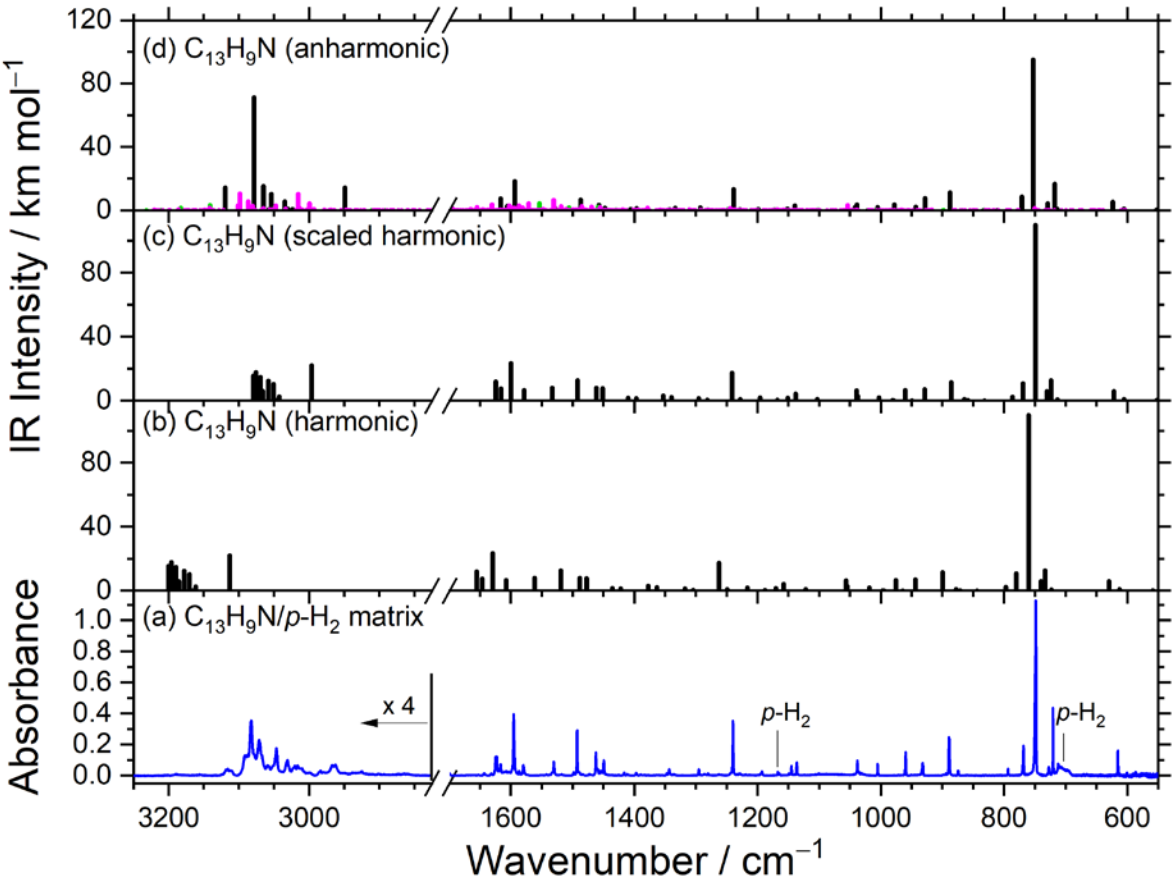

Figure S5. Comparison of experimental and predicted IR spectra of $C_{13}H_9N$. (a) Experimental absorption spectrum of a $C_{13}H_9N$/*p*-H_2 matrix. Absorptions near 700 cm^{-1} and at 1167 cm^{-1} originate from solid *p*-H_2. (b) Simulated stick spectrum of $C_{13}H_9N$ based on harmonic vibrational wavenumbers and IR intensities. (c) Simulated stick spectrum of $C_{13}H_9N$ based on IR intensities and harmonic vibrational wavenumbers scaled according to 0.9510 x + 35.9 for wavenumbers > 2000 cm^{-1} and 0.9778 x + 6.3 for wavenumbers < 2000 cm^{-1}. (d) Simulated stick spectrum of $C_{13}H_9N$ based on anharmonic vibrational wavenumbers and IR intensities; the IR stick spectra of overtone and combination bands are presented with green and pink colors, respectively. Both harmonic and anharmonic vibrational calculations were predicted with the B3LYP/6-311++G(d,p) method. The observed and predicted wavenumbers and IR intensities are listed in Table S2.

(a)

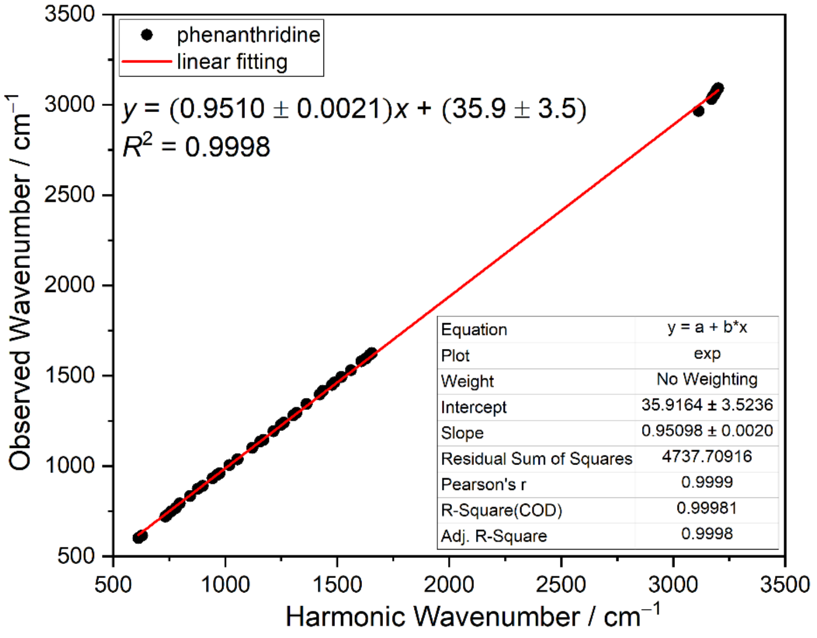

(b)

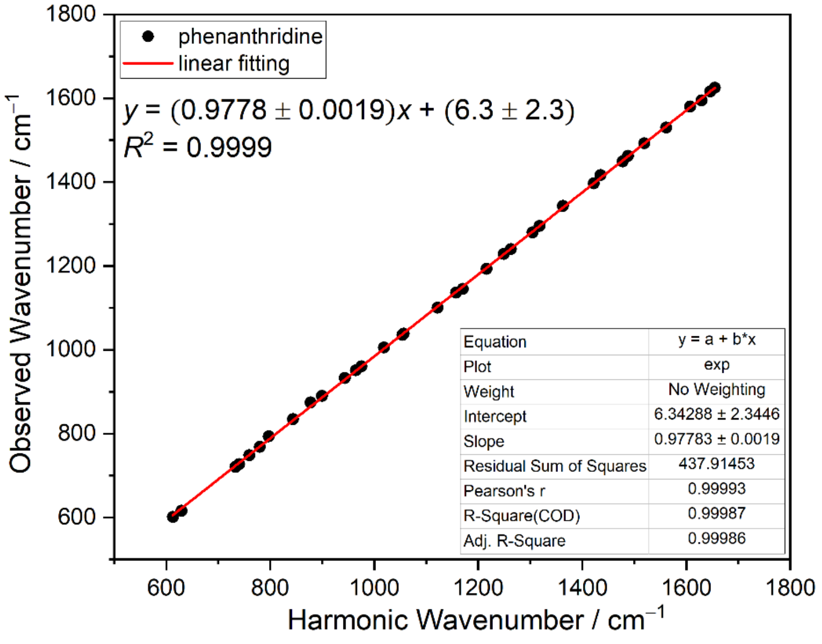

Figure S6. Linear fit of observed and calculated harmonic vibrational wavenumbers of $C_{13}H_9N$ in two spectral ranges. (a) 500−3500 cm^{-1}; (b) 500−1800 cm^{-1}. Harmonic vibrational wavenumbers were calculated with the B3LYP/6-311++G(d,p) method. The linear equations obtained on fitting the data for traces (a) and (b) are $y = (0.9510 \pm 0.0021)\,x + (35.9 \pm 3.5)$ and $y = (0.9778 \pm 0.0019)\,x + (6.3 \pm 2.3)$, respectively; y is the scaled vibrational wavenumber and x is the harmonic vibrational wavenumber.

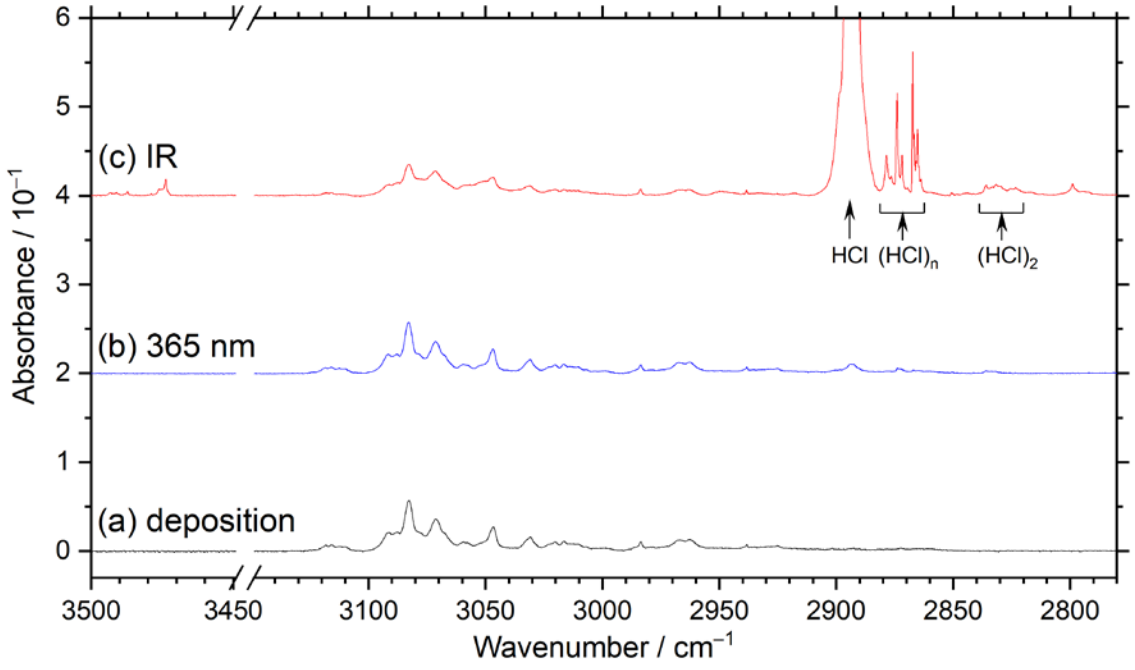

(c) IR
(b) 365 nm
(a) deposition
HCl
(HCl)n
(HCl)2
Absorbance / 10−1
Wavenumber / cm−1

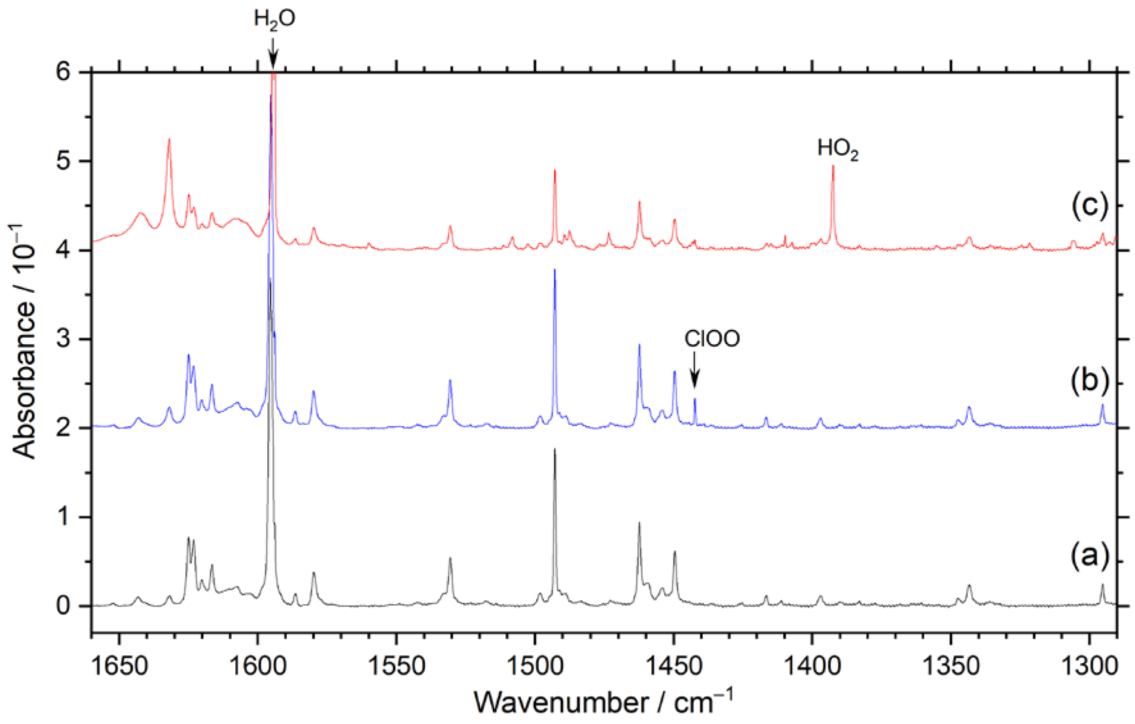

H2O
HO2
ClOO
(c)
(b)
(a)
Absorbance / 10−1
Wavenumber / cm−1

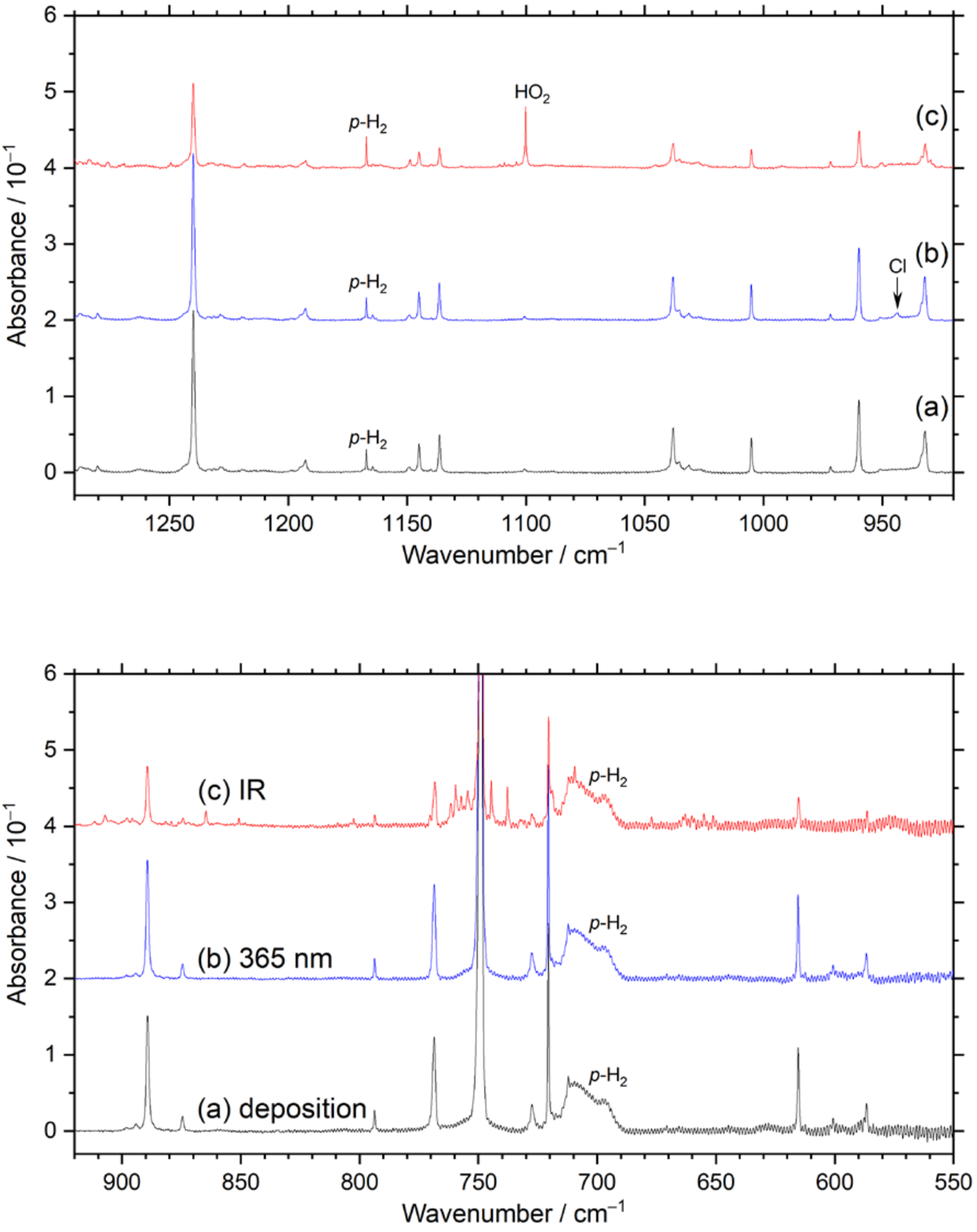

Figure S7. Spectra of a $C_{13}H_9N/Cl_2/p\text{-}H_2$ matrix before and after irradiation with UV/IR light. (a) Spectrum of a $C_{13}H_9N/Cl_2/p\text{-}H_2$ matrix deposited at 3.2 K for 7 h. (b) Spectrum of the matrix after irradiation at 365 nm for 1 h. (c) Spectrum of the matrix after subsequent irradiation with IR light for 2 h. Baselines were shifted for clarity.

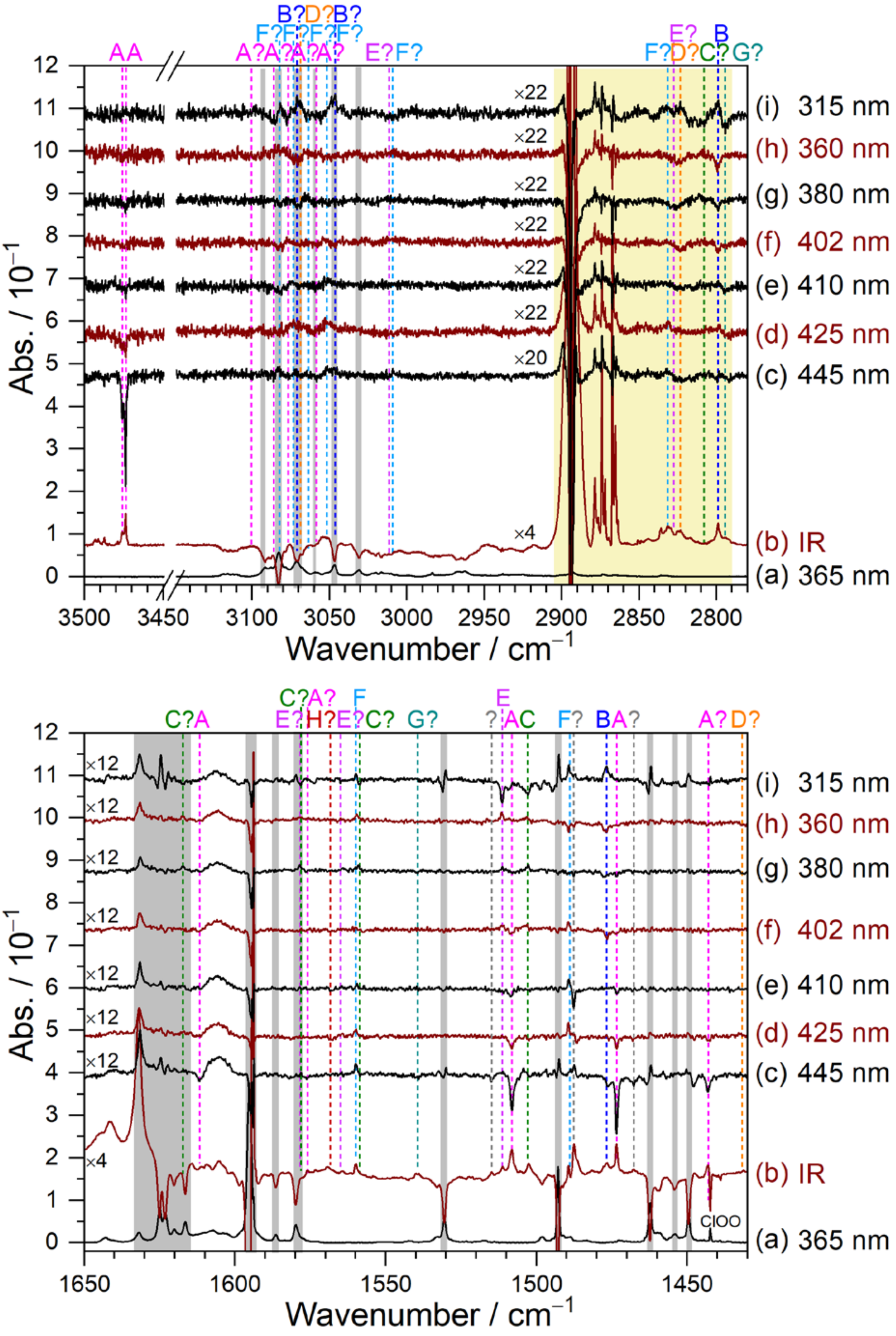

Abs. / 10⁻¹
Wavenumber / cm⁻¹
(i) 315 nm
(h) 360 nm
(g) 380 nm
(f) 402 nm
(e) 410 nm
(d) 425 nm
(c) 445 nm
(b) IR
(a) 365 nm
×22
×20
×4
×12
ClOO

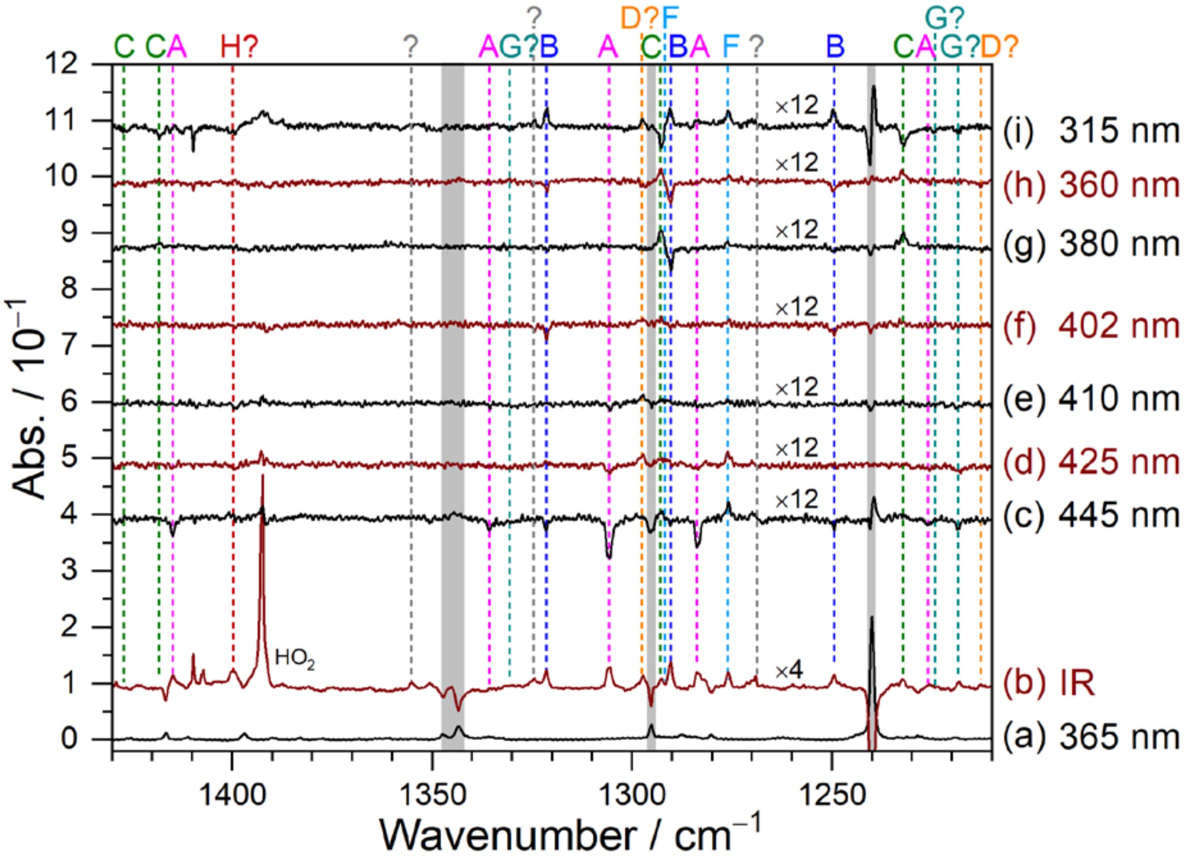
C C A H? ? A G? ? B A D? ? C F B A F ? B C A G? G? D?
(i) 315 nm
(h) 360 nm
(g) 380 nm
(f) 402 nm
(e) 410 nm
(d) 425 nm
(c) 445 nm
(b) IR
(a) 365 nm
×12
×4
HO2
Abs. / 10−1
Wavenumber / cm−1

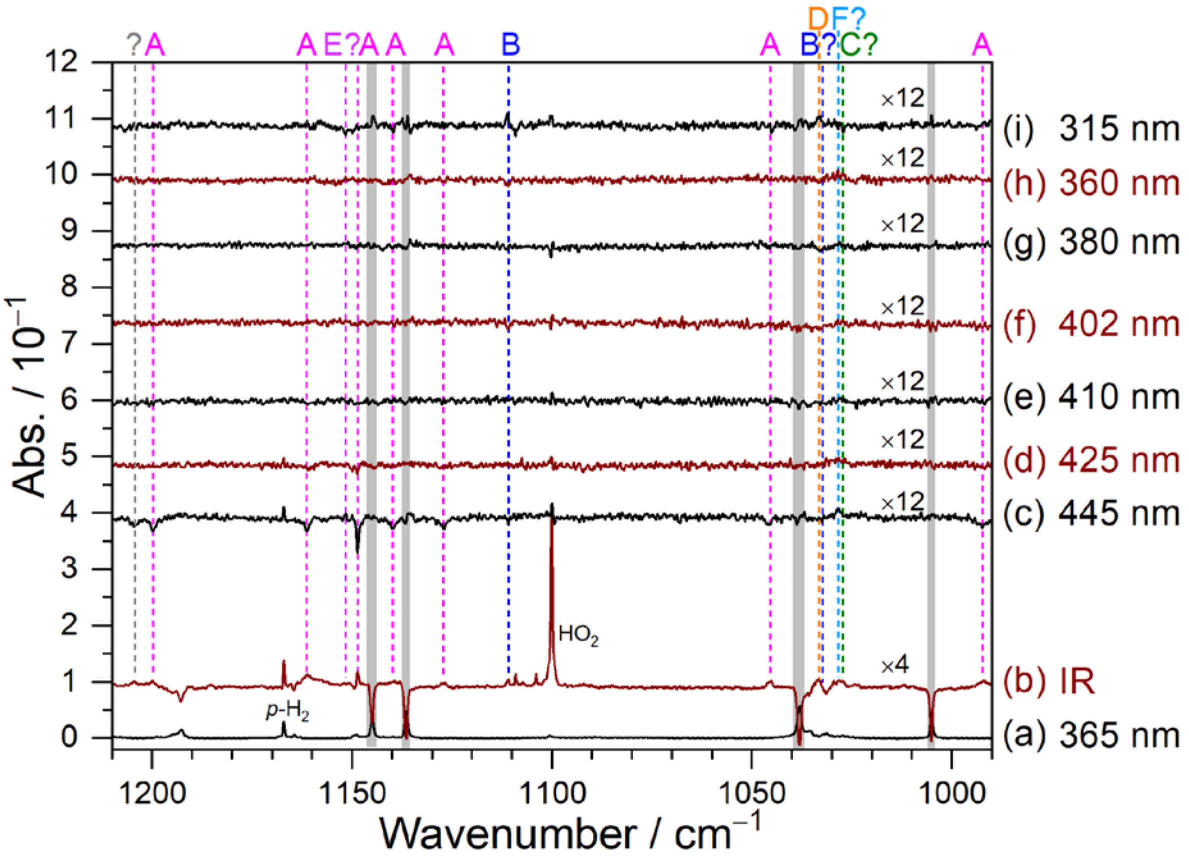
? A A E? A A A B A D B? F? C? A
(i) 315 nm
(h) 360 nm
(g) 380 nm
(f) 402 nm
(e) 410 nm
(d) 425 nm
(c) 445 nm
(b) IR
(a) 365 nm
×12
×4
HO2
p-H2
Abs. / 10−1
Wavenumber / cm−1

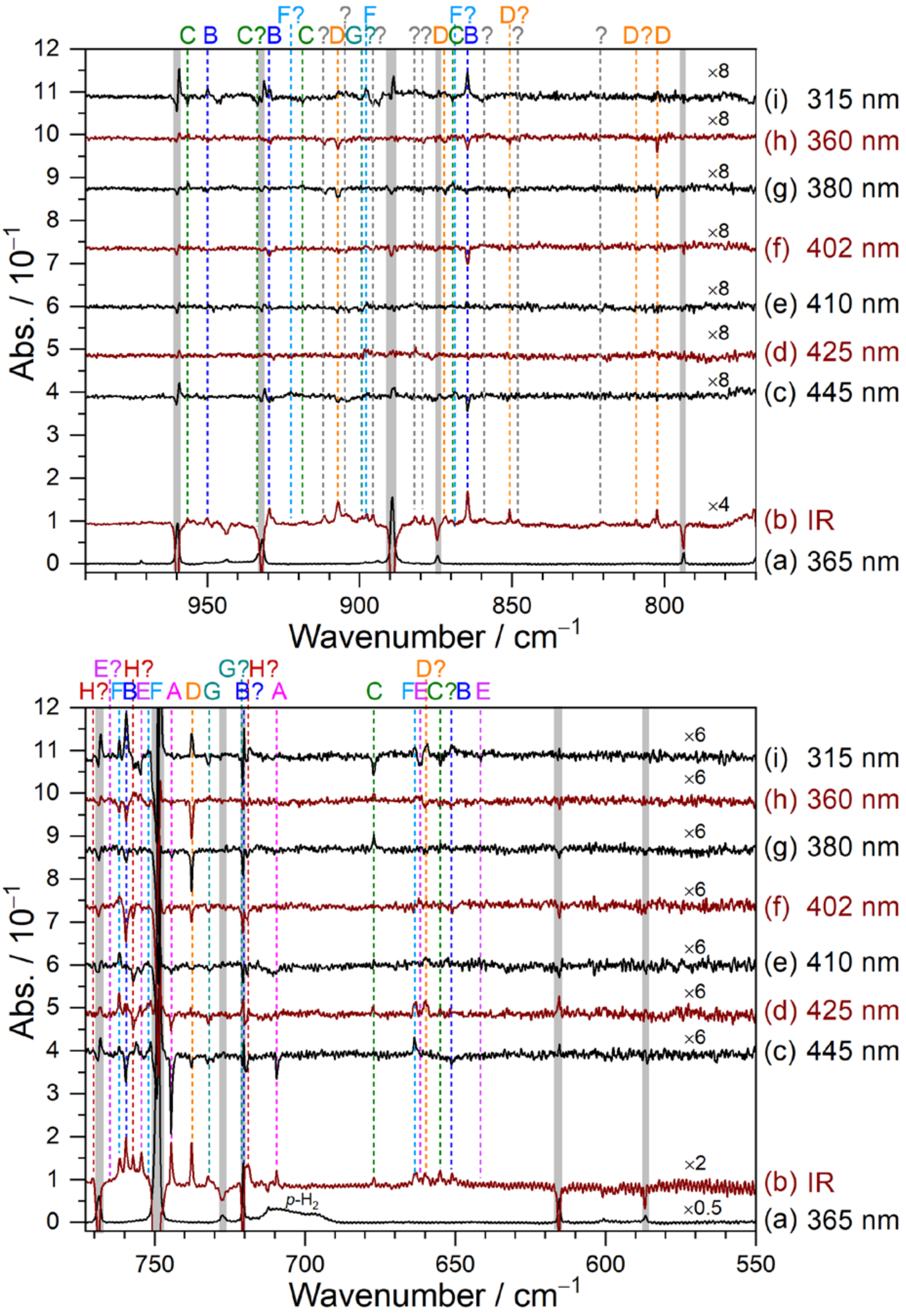

(i) 315 nm
(h) 360 nm
(g) 380 nm
(f) 402 nm
(e) 410 nm
(d) 425 nm
(c) 445 nm
(b) IR
(a) 365 nm
Abs. / 10^{-1}
Wavenumber / cm^{-1}
p-H_2

Figure S8. Spectra of a $C_{13}H_9N/Cl_2/p\text{-}H_2$ matrix in regions 3500−3450, 3150−2780, and 1650−550 cm^{-1} at various stages of the experiment. (a) Spectrum of a $C_{13}H_9N/Cl_2/p\text{-}H_2$ matrix deposited at 3.2 K for 7 h and irradiated at 365 nm for 1 h. (b) Difference spectrum of the matrix after subsequent irradiation with IR light for 2 h. Difference spectra of the matrix after further sequential irradiation at 445 nm (c), 425 nm (d), 410 nm (e), 402 nm (f), 380 nm (g), 360 nm (h), and 315 nm (i); each step is 20 min. Lines in groups A ($C_{13}H_9NH$), B (9-$HC_{13}H_9N$), C (1-$HC_{13}H_9N$), D (2-$HC_{13}H_9N$), E (10-$HC_{13}H_9N$), F (7-$HC_{13}H_9N$), G (3-$HC_{13}H_9N$), and H (6-$HC_{13}H_9N$) are indicated with color-coded dashed lines and labels. Spectral regions subjected to interference from the intense absorption of $C_{13}H_9N$ are shaded gray and those from absorption of HCl and HCl complexes are shaded yellow.

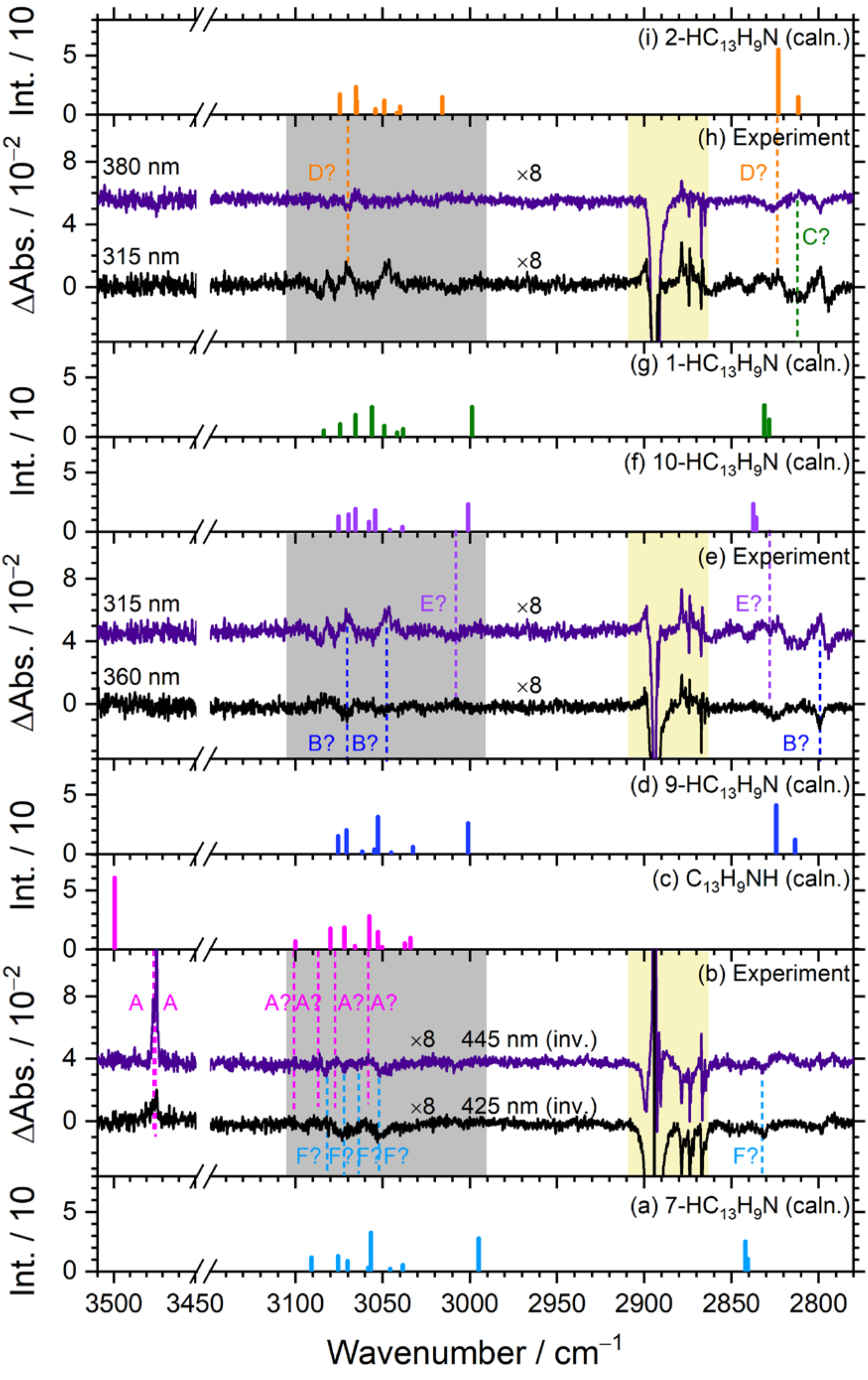
(i) 2-HC13H9N (caln.)
(h) Experiment
380 nm
315 nm
D?
D?
C?
×8
(g) 1-HC13H9N (caln.)
(f) 10-HC13H9N (caln.)
(e) Experiment
315 nm
360 nm
E?
B?
(d) 9-HC13H9N (caln.)
(c) C13H9NH (caln.)
(b) Experiment
A
A?
445 nm (inv.)
425 nm (inv.)
F?
(a) 7-HC13H9N (caln.)
Int. / 10
ΔAbs. / 10−2
Wavenumber / cm−1

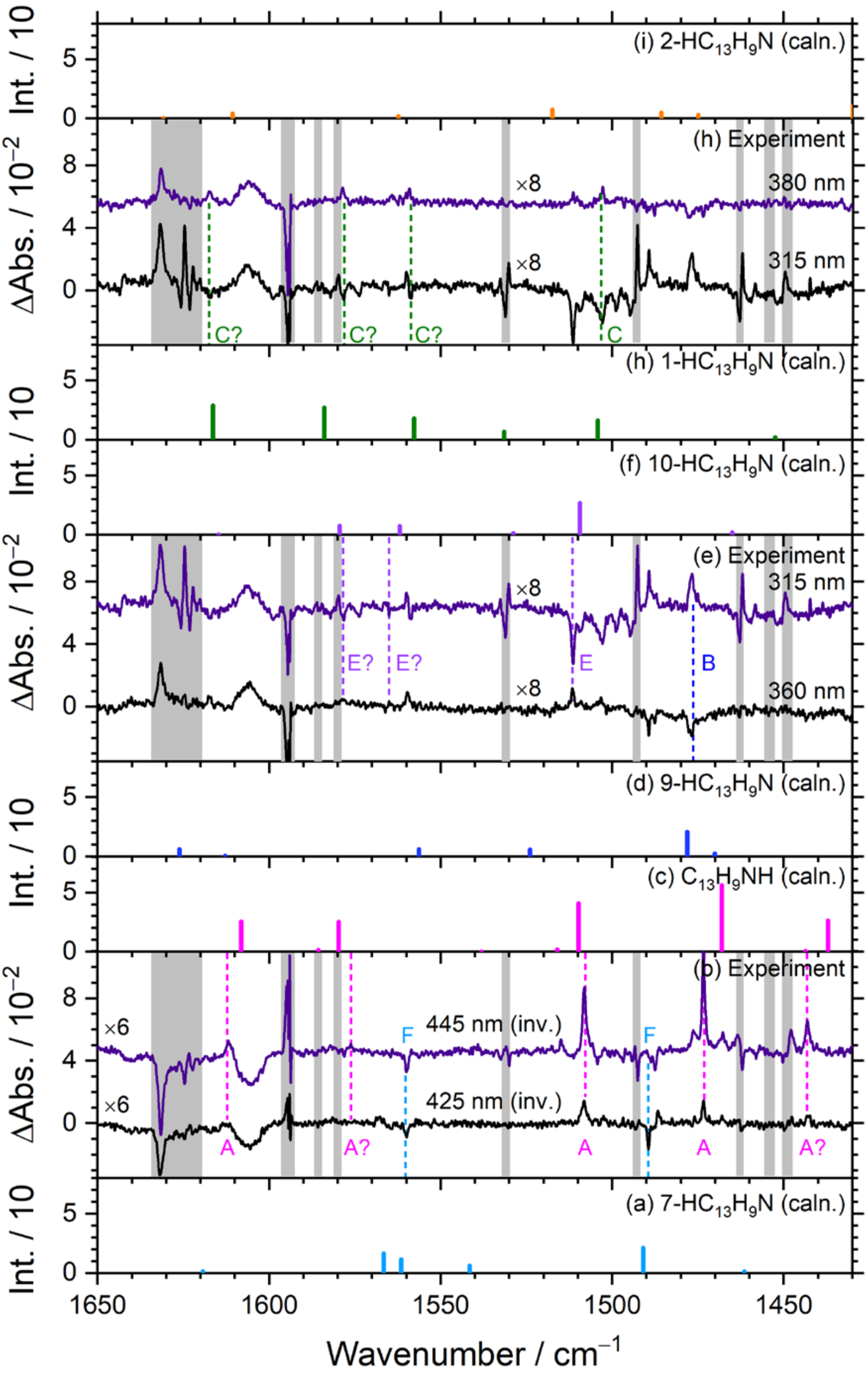

(i) 2-HC13H9N (caln.)
(h) Experiment
380 nm
315 nm
×8
×8
C?
C?
C?
C
(h) 1-HC13H9N (caln.)
(f) 10-HC13H9N (caln.)
(e) Experiment
315 nm
360 nm
×8
×8
E?
E?
E
B
(d) 9-HC13H9N (caln.)
(c) C13H9NH (caln.)
(b) Experiment
445 nm (inv.)
425 nm (inv.)
×6
×6
F
F
A
A?
A
A
A?
(a) 7-HC13H9N (caln.)
Int. / 10
ΔAbs. / 10−2
Wavenumber / cm−1

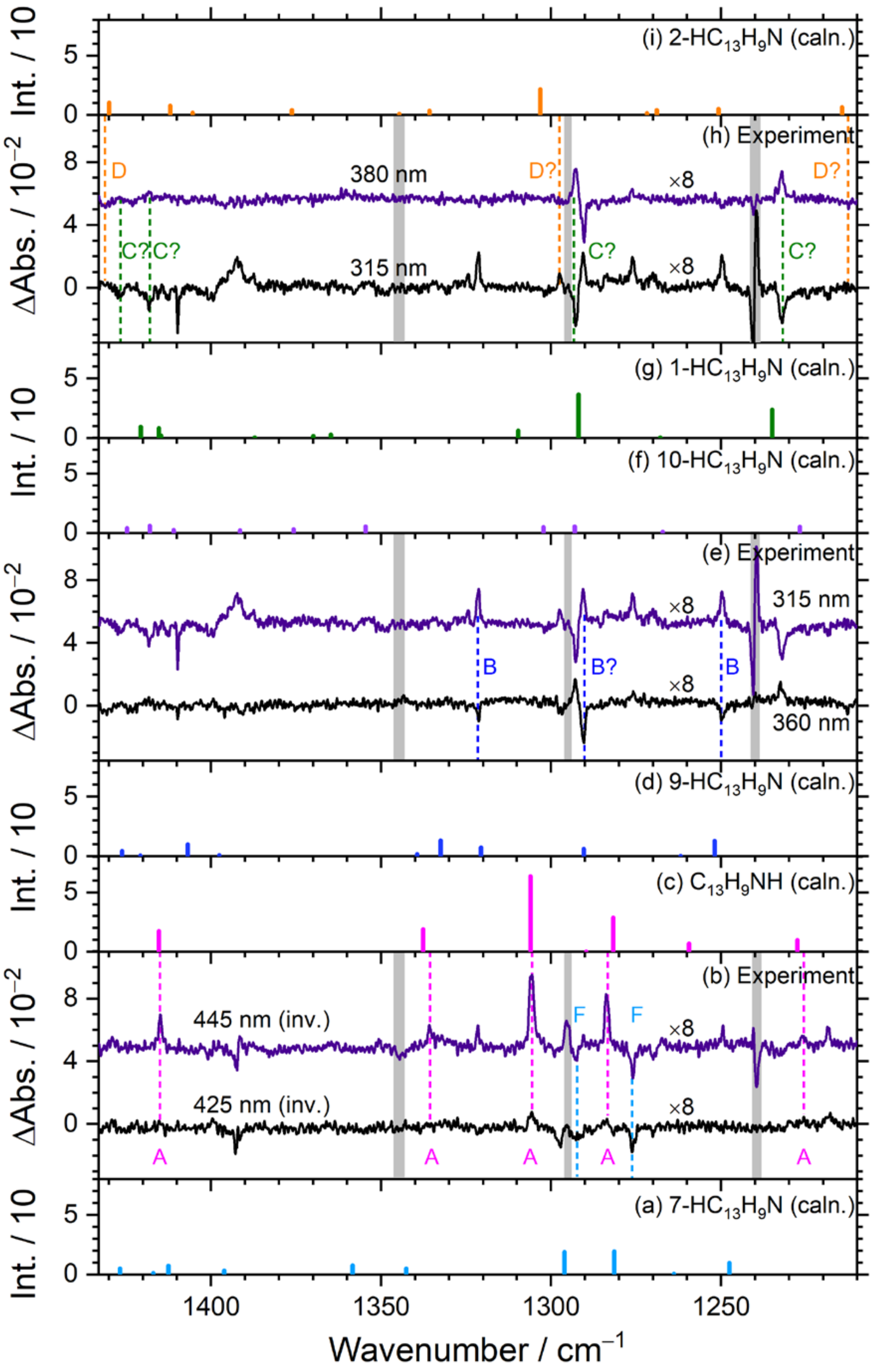

(i) 2-HC13H9N (caln.)
(h) Experiment
380 nm
315 nm
×8
D
D?
C?
(g) 1-HC13H9N (caln.)
(f) 10-HC13H9N (caln.)
(e) Experiment
315 nm
360 nm
B
B?
(d) 9-HC13H9N (caln.)
(c) C13H9NH (caln.)
(b) Experiment
445 nm (inv.)
425 nm (inv.)
A
F
(a) 7-HC13H9N (caln.)
Int. / 10
ΔAbs. / 10−2
Wavenumber / cm−1

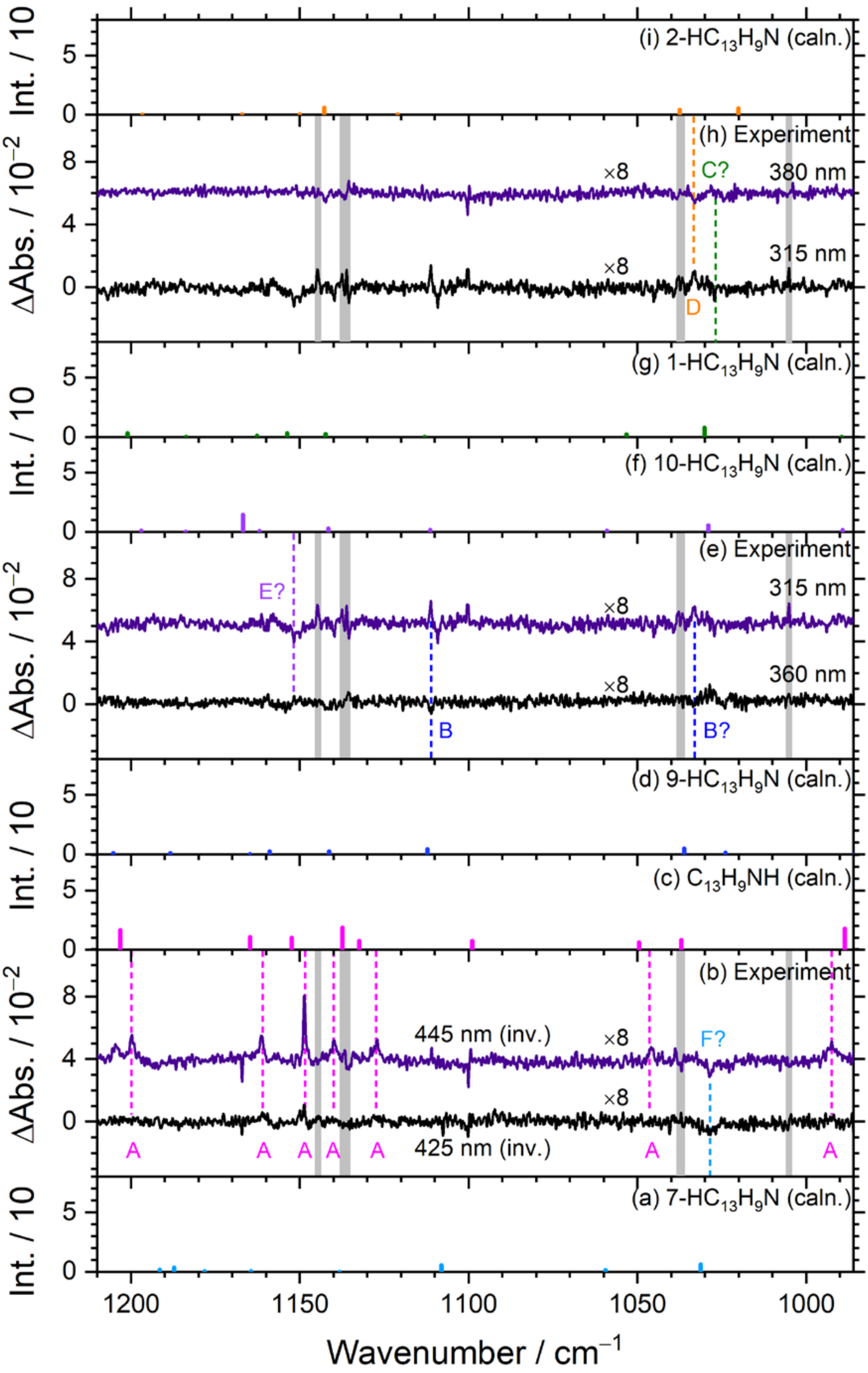
(i) 2-HC$_{13}$H$_9$N (caln.)
(h) Experiment
380 nm
315 nm
×8
C?
D
(g) 1-HC$_{13}$H$_9$N (caln.)
(f) 10-HC$_{13}$H$_9$N (caln.)
(e) Experiment
315 nm
360 nm
E?
B
B?
(d) 9-HC$_{13}$H$_9$N (caln.)
(c) C$_{13}$H$_9$NH (caln.)
(b) Experiment
445 nm (inv.)
425 nm (inv.)
F?
A
(a) 7-HC$_{13}$H$_9$N (caln.)
Int. / 10
ΔAbs. / 10^{-2}
Wavenumber / cm^{-1}

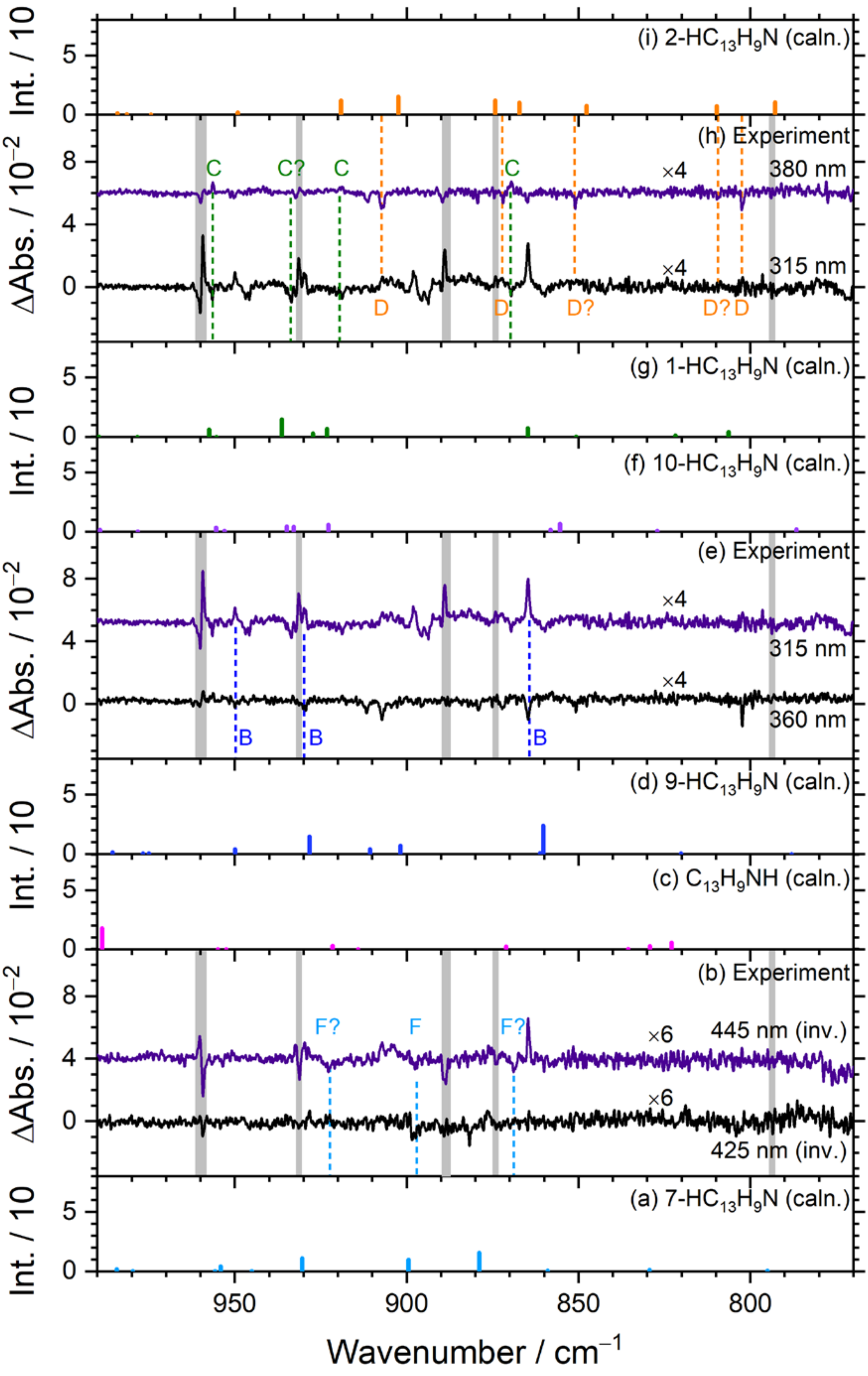
(i) 2-HC13H9N (caln.)
(h) Experiment
380 nm
315 nm
×4
C
C?
D
D?
(g) 1-HC13H9N (caln.)
(f) 10-HC13H9N (caln.)
(e) Experiment
315 nm
360 nm
B
(d) 9-HC13H9N (caln.)
(c) C13H9NH (caln.)
(b) Experiment
445 nm (inv.)
425 nm (inv.)
×6
F
F?
(a) 7-HC13H9N (caln.)
Int. / 10
ΔAbs. / 10−2
Wavenumber / cm−1
950
900
850
800

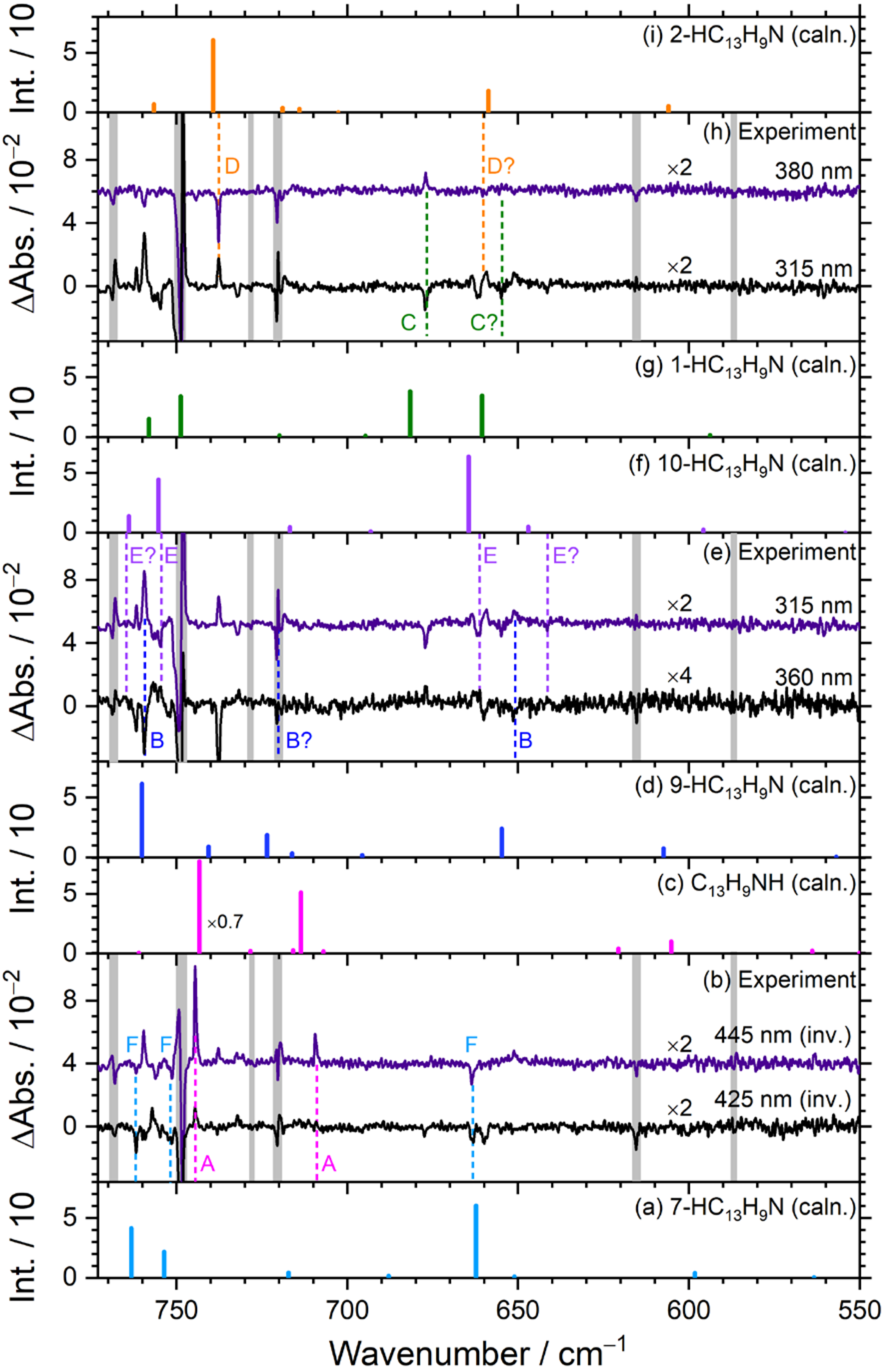

(i) 2-HC$_{13}$H$_9$N (caln.)
(h) Experiment
D
D?
×2
380 nm
×2
315 nm
C
C?
(g) 1-HC$_{13}$H$_9$N (caln.)
(f) 10-HC$_{13}$H$_9$N (caln.)
E? E
E
E?
(e) Experiment
×2
315 nm
×4
360 nm
B
B?
B
(d) 9-HC$_{13}$H$_9$N (caln.)
(c) C$_{13}$H$_9$NH (caln.)
×0.7
(b) Experiment
F F
F
×2
445 nm (inv.)
×2
425 nm (inv.)
A
A
(a) 7-HC$_{13}$H$_9$N (caln.)
Int. / 10
ΔAbs. / 10^{-2}
Wavenumber / cm^{-1}
750
700
650
600
550

Figure S9. Comparison of observed lines in groups A−F in the $C_{13}H_9N/Cl_2/p\text{-}H_2$ experiments in regions 3500−3450, 3150−2780, and 1650−550 cm^{-1} with theoretically predicted stick spectra of assigned isomers of hydrogenated phenanthridine ($HC_{13}H_9N$). (a) IR stick spectrum of 7-$HC_{13}H_9NH$. (b) Difference spectra after secondary photolysis at 425 nm (lower trace, from Figure S8d), and after secondary photolysis at 445 nm (upper trace, from Figure S8c, inverted); lines of groups A and F are indicated with pink and light blue dashed lines and labels. (C) IR stick spectrum of $C_{13}H_9NH$. (d) IR stick spectrum of 9-$HC_{13}H_9NH$. (e) Difference spectra after secondary photolysis at 360 nm (lower trace, from Figure S8h), and after secondary photolysis at 315 nm (Figure S8i); lines of groups B and E are indicated with blue and purple dashed lines and labels. (f) IR stick spectrum of 10-$HC_{13}H_9N$. (g) IR stick spectrum of 1-$HC_{13}H_9NH$. (h) Difference spectra after secondary photolysis at 315 nm (lower trace, from Figure S8i), and after secondary photolysis at 380 nm (Figure S8g); lines of groups C and D are indicated with green and orange dashed lines and labels. (i) IR stick spectrum of 2-$HC_{13}H_9N$. All IR stick spectra are based on the scaled harmonic vibrational wavenumbers and IR intensities predicted using the B3LYP/6-311++G(d,p) method. The predicted IR intensities are in km mol^{-1}. Spectral regions subjected to interference by the intense absorption of $C_{13}H_9N$ are shaded gray and those by absorption of HCl and HCl complexes are shaded yellow.

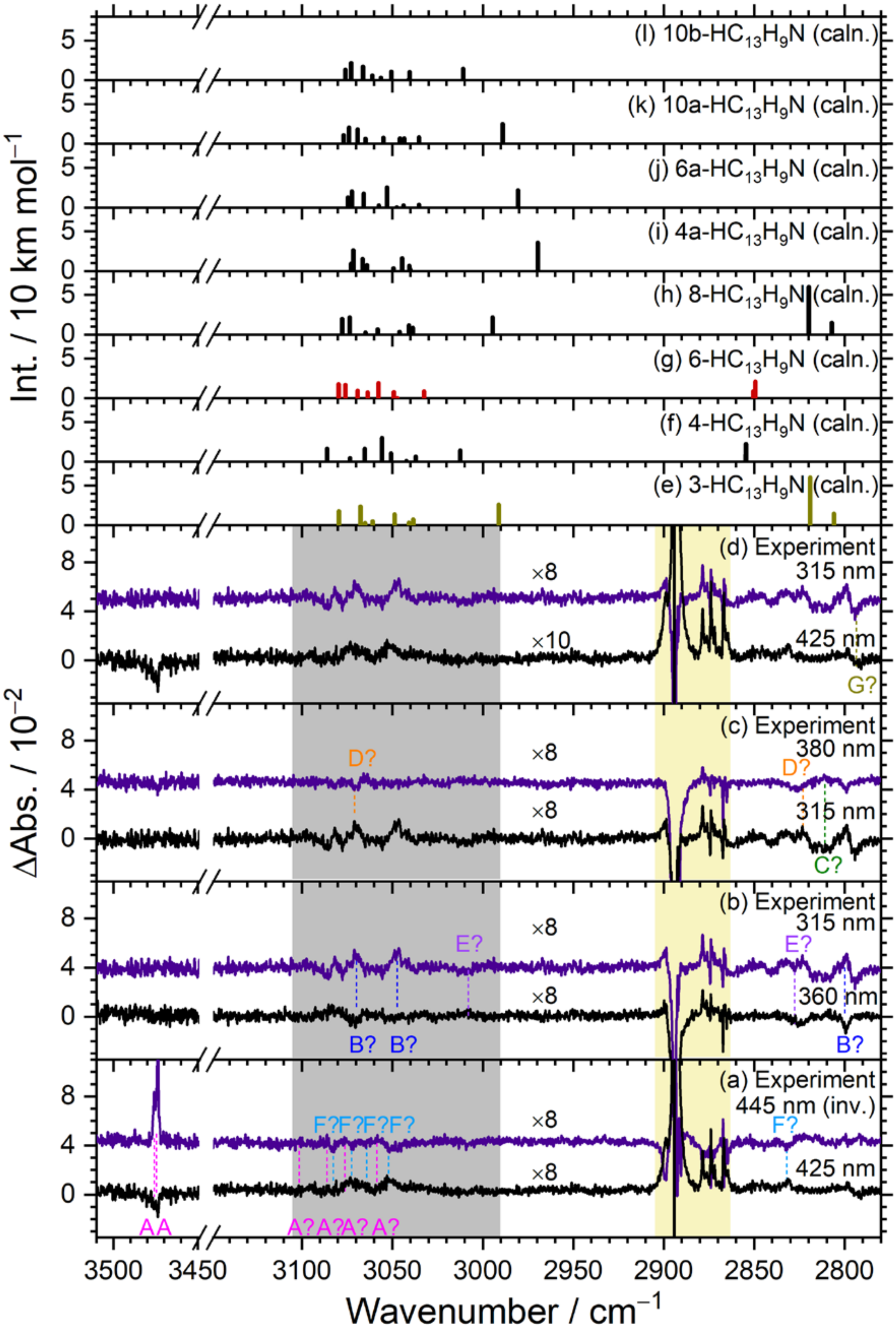

(l) 10b-$HC_{13}H_9N$ (caln.)
(k) 10a-$HC_{13}H_9N$ (caln.)
(j) 6a-$HC_{13}H_9N$ (caln.)
(i) 4a-$HC_{13}H_9N$ (caln.)
(h) 8-$HC_{13}H_9N$ (caln.)
(g) 6-$HC_{13}H_9N$ (caln.)
(f) 4-$HC_{13}H_9N$ (caln.)
(e) 3-$HC_{13}H_9N$ (caln.)
Int. / 10 km mol^{-1}
(d) Experiment
315 nm
425 nm
×8
×10
G?
(c) Experiment
380 nm
315 nm
D?
C?
(b) Experiment
315 nm
360 nm
E?
B?
(a) Experiment
445 nm (inv.)
425 nm
F?
A
A?
ΔAbs. / 10^{-2}
Wavenumber / cm^{-1}

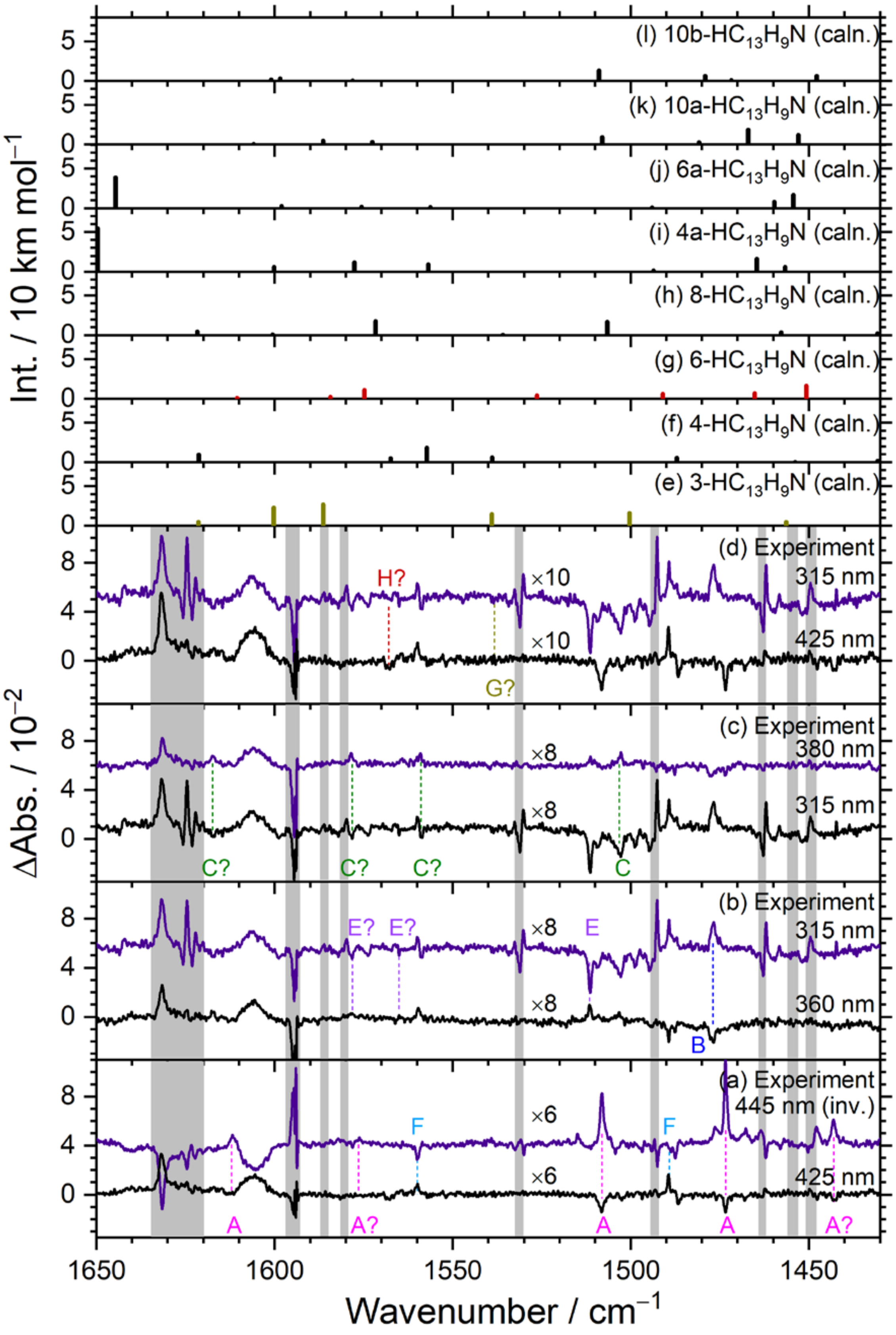

(l) 10b-HC13H9N (caln.)
(k) 10a-HC13H9N (caln.)
(j) 6a-HC13H9N (caln.)
(i) 4a-HC13H9N (caln.)
(h) 8-HC13H9N (caln.)
(g) 6-HC13H9N (caln.)
(f) 4-HC13H9N (caln.)
(e) 3-HC13H9N (caln.)
(d) Experiment
315 nm
425 nm
(c) Experiment
380 nm
315 nm
(b) Experiment
315 nm
360 nm
(a) Experiment
445 nm (inv.)
425 nm
Int. / 10 km mol−1
ΔAbs. / 10−2
Wavenumber / cm−1

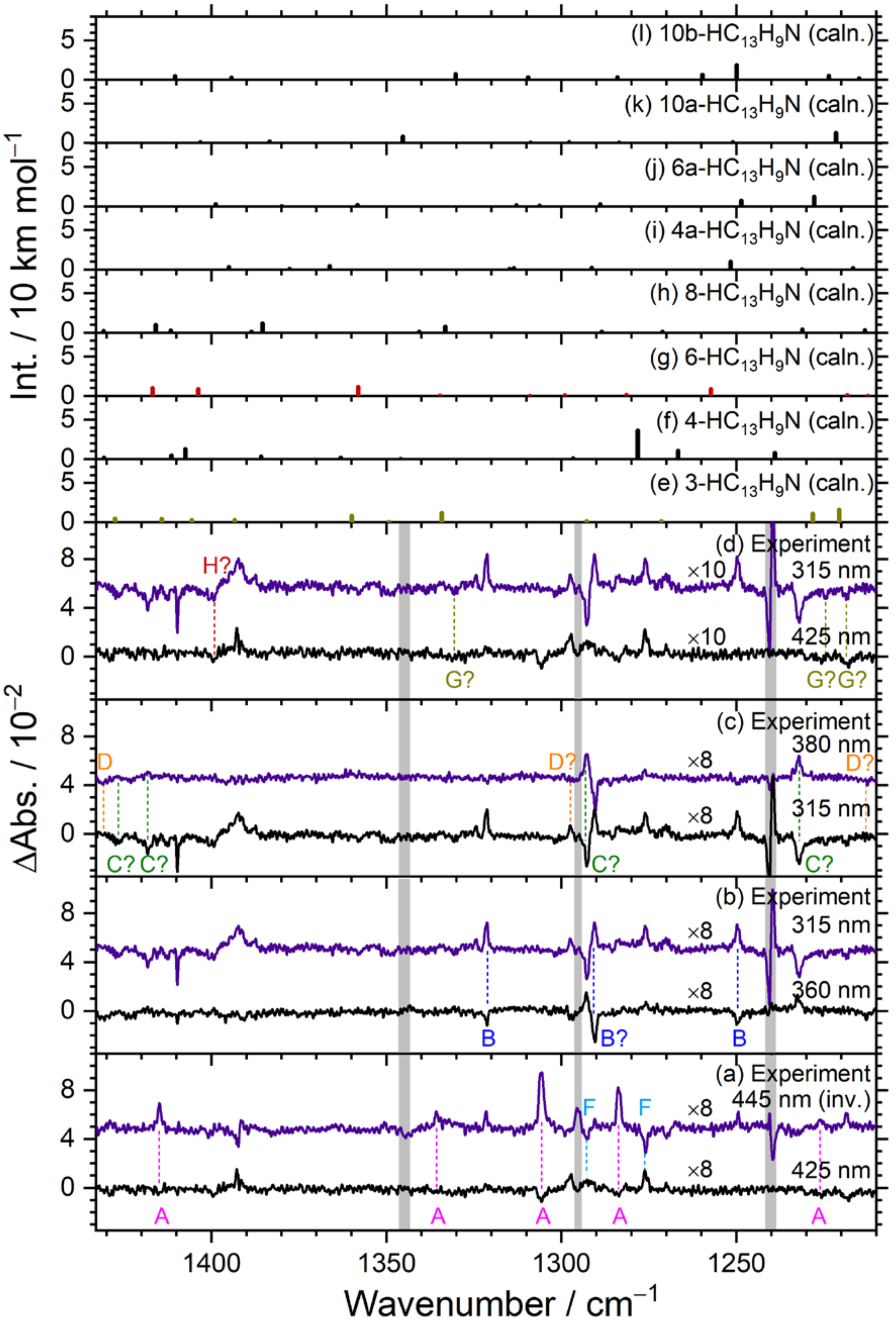

(l) 10b-$HC_{13}H_9N$ (caln.)
(k) 10a-$HC_{13}H_9N$ (caln.)
(j) 6a-$HC_{13}H_9N$ (caln.)
(i) 4a-$HC_{13}H_9N$ (caln.)
(h) 8-$HC_{13}H_9N$ (caln.)
(g) 6-$HC_{13}H_9N$ (caln.)
(f) 4-$HC_{13}H_9N$ (caln.)
(e) 3-$HC_{13}H_9N$ (caln.)
(d) Experiment
315 nm
425 nm
×10
H?
G?
G?G?
(c) Experiment
380 nm
315 nm
×8
D
D?
C?
(b) Experiment
315 nm
360 nm
×8
B
B?
(a) Experiment
445 nm (inv.)
425 nm
×8
A
F
Int. / 10 km mol^{-1}
ΔAbs. / 10^{-2}
Wavenumber / cm^{-1}

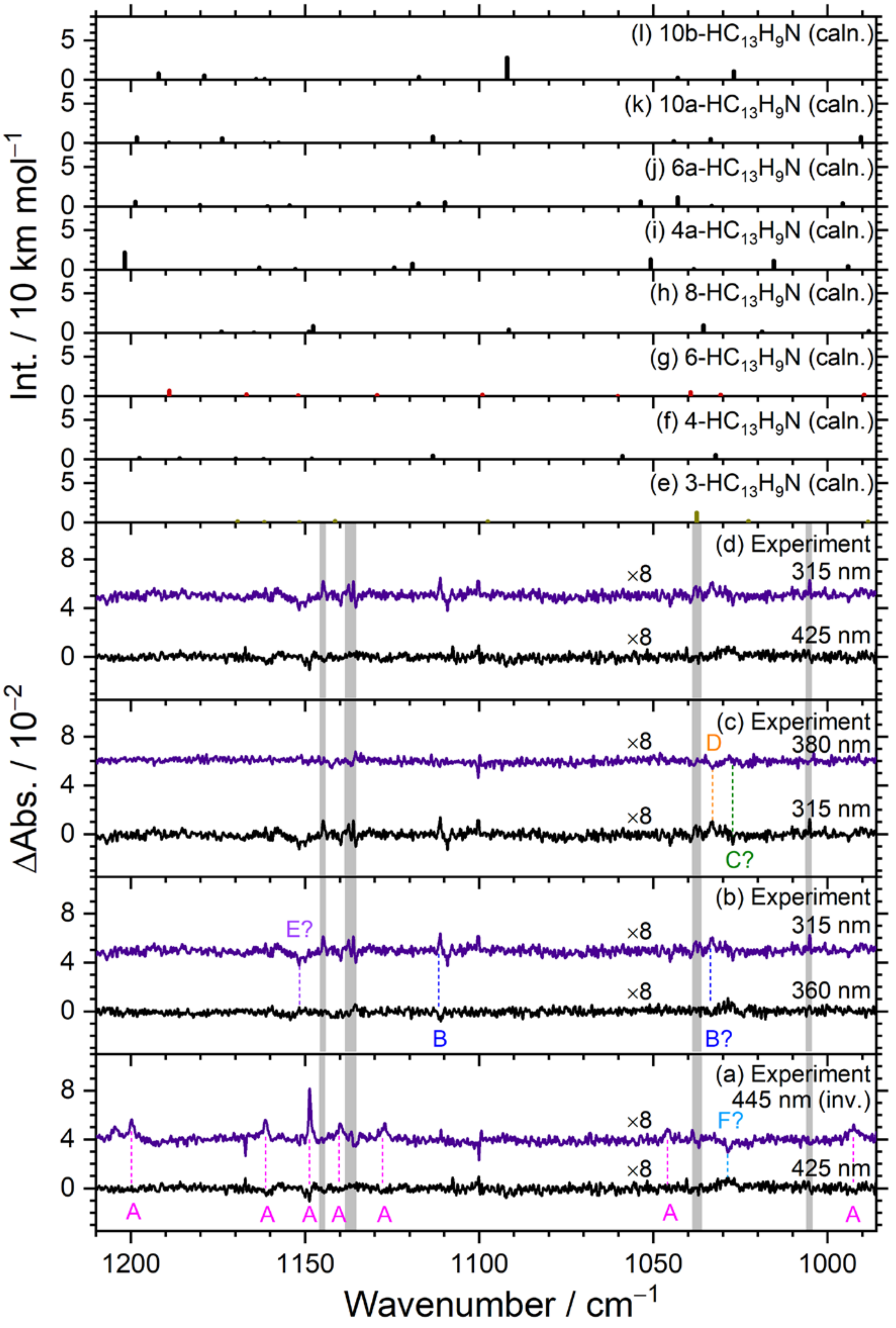
(l) 10b-HC13H9N (caln.)
(k) 10a-HC13H9N (caln.)
(j) 6a-HC13H9N (caln.)
(i) 4a-HC13H9N (caln.)
(h) 8-HC13H9N (caln.)
(g) 6-HC13H9N (caln.)
(f) 4-HC13H9N (caln.)
(e) 3-HC13H9N (caln.)
(d) Experiment
315 nm
425 nm
(c) Experiment
380 nm
315 nm
D
C?
(b) Experiment
315 nm
360 nm
E?
B
B?
(a) Experiment
445 nm (inv.)
425 nm
F?
A
×8
Int. / 10 km mol−1
ΔAbs. / 10−2
Wavenumber / cm−1

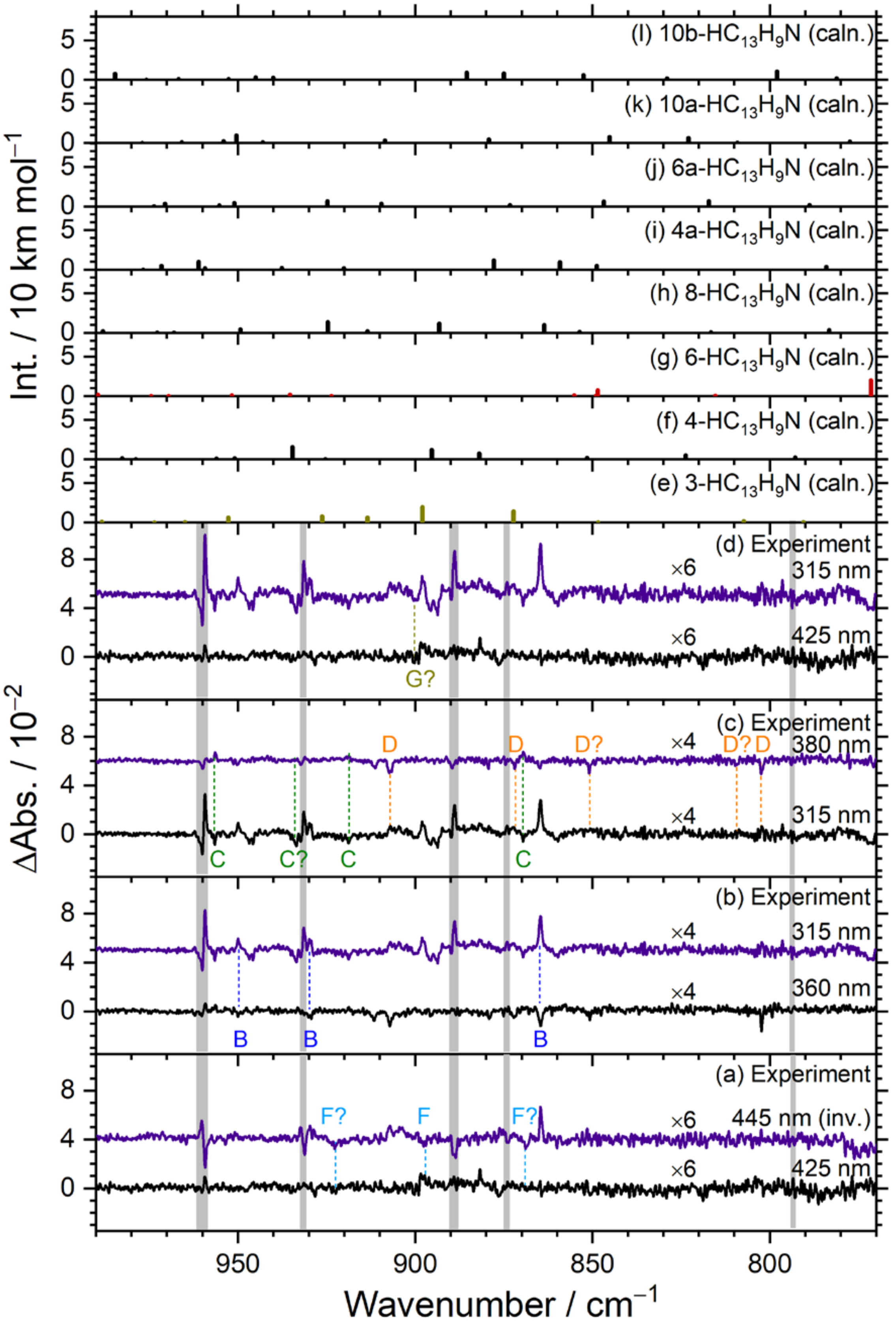

(l) 10b-$HC_{13}H_9N$ (caln.)
(k) 10a-$HC_{13}H_9N$ (caln.)
(j) 6a-$HC_{13}H_9N$ (caln.)
(i) 4a-$HC_{13}H_9N$ (caln.)
(h) 8-$HC_{13}H_9N$ (caln.)
(g) 6-$HC_{13}H_9N$ (caln.)
(f) 4-$HC_{13}H_9N$ (caln.)
(e) 3-$HC_{13}H_9N$ (caln.)
Int. / 10 km mol^{-1}
(d) Experiment
×6 315 nm
×6 425 nm
G?
(c) Experiment
×4 380 nm
×4 315 nm
D D D? D? D
C C? C C
(b) Experiment
×4 315 nm
×4 360 nm
B B B
(a) Experiment
×6 445 nm (inv.)
×6 425 nm
F? F F?
ΔAbs. / 10^{-2}
Wavenumber / cm^{-1}

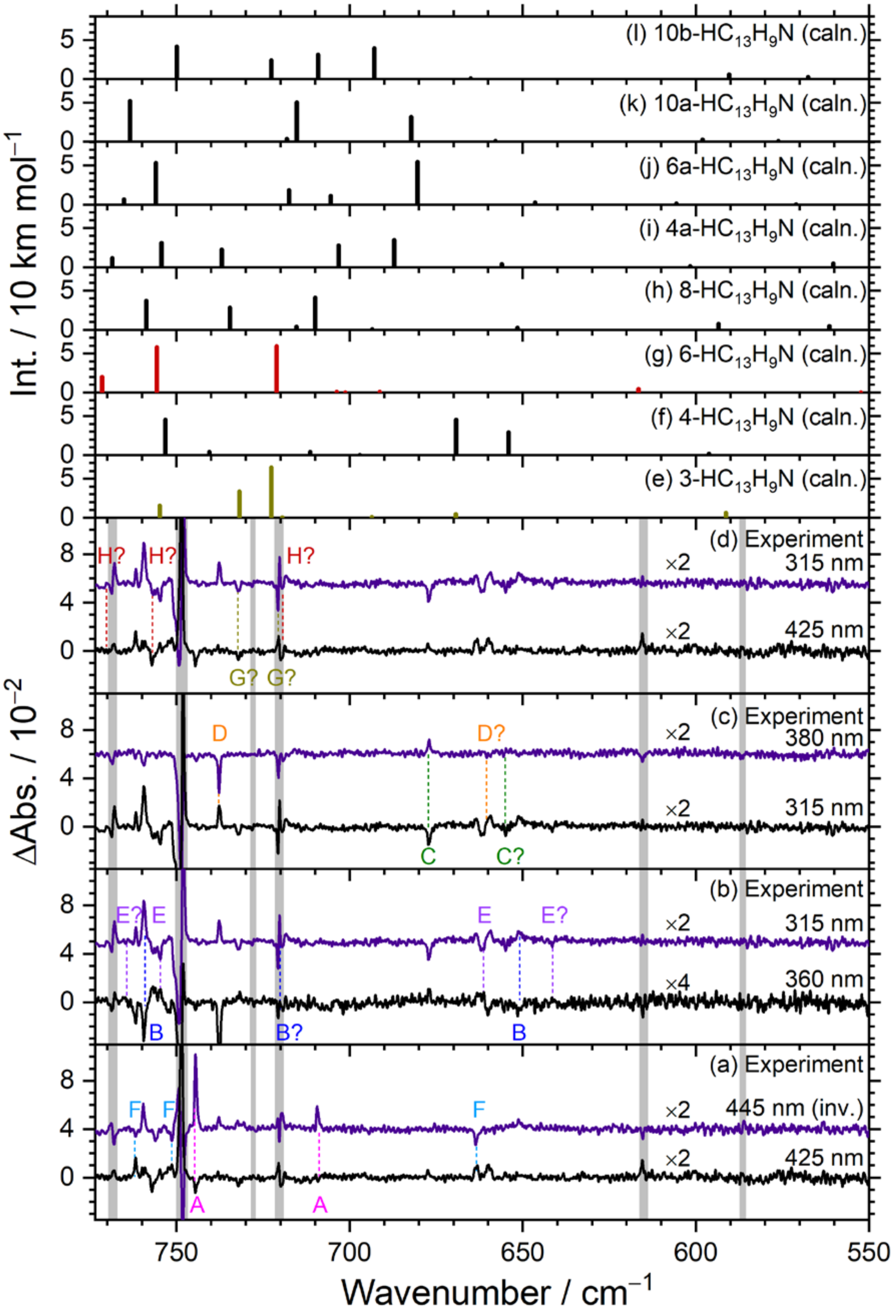

(l) 10b-HC13H9N (caln.)
(k) 10a-HC13H9N (caln.)
(j) 6a-HC13H9N (caln.)
(i) 4a-HC13H9N (caln.)
(h) 8-HC13H9N (caln.)
(g) 6-HC13H9N (caln.)
(f) 4-HC13H9N (caln.)
(e) 3-HC13H9N (caln.)
(d) Experiment
315 nm
425 nm
(c) Experiment
380 nm
315 nm
(b) Experiment
315 nm
360 nm
(a) Experiment
445 nm (inv.)
425 nm
Int. / 10 km mol−1
ΔAbs. / 10−2
Wavenumber / cm−1

Figure S10. Comparison of the observed lines of groups A−H in regions 3500−3450, 3150−2780, and 1650−550 cm^{-1} with theoretically predicted stick spectra of unassigned isomers of hydrogenated phenanthridine ($HC_{13}H_9N$). (a) Difference spectra after secondary photolysis at 425 nm (lower trace, from Figure S8d), and after secondary photolysis at 445 nm (upper trace, from Figure S8c, inverted); lines of groups A and F are indicated with pink and light blue dashed lines and labels. (b) Difference spectra after secondary photolysis at 360 nm (lower trace, from Figure S8h), and after secondary photolysis at 315 nm (Figure S8i); lines of groups B and E are indicated with blue and purple dashed lines and labels. (c) Difference spectra after secondary photolysis at 315 nm (lower trace, from Figure S8i), and after secondary photolysis at 380 nm (Figure S8g); lines of groups C and D are indicated with green and orange dashed lines and labels. (d) Difference spectra after secondary photolysis at 425 nm (lower trace, from Figure S8d), and after secondary photolysis at 315 nm (Figure S8i); lines of groups G and H are indicated with olive and red dashed lines and labels. IR stick spectra of 3-$HC_{13}H_9N$ (e), 4-$HC_{13}H_9N$ (f), 6-$HC_{13}H_9NH$ (g), 8-$HC_{13}H_9N$ (h), 4a-$HC_{13}H_9NH$ (i), 6a-$HC_{13}H_9N$ (j), 10a-$HC_{13}H_9N$ (k), and 10b-$HC_{13}H_9N$ (l) are simulated based on scaled harmonic vibrational wavenumbers and IR intensities predicted using the B3LYP/6-311++G(d,p) method. Spectral regions subjected to interference by the intense absorption of $C_{13}H_9N$ are shaded gray and those by absorption of HCl and HCl complexes are shaded yellow.

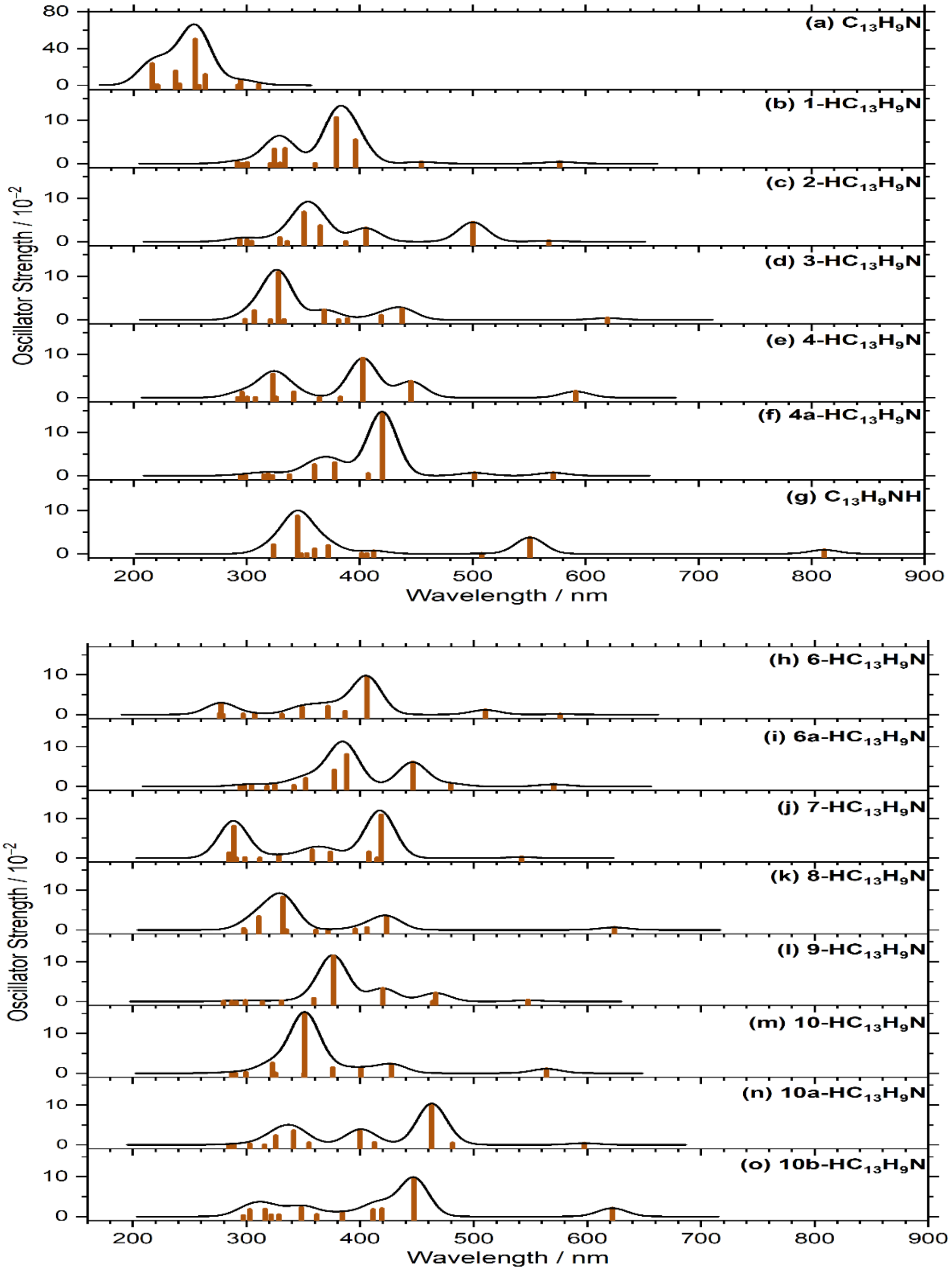

Figure S11. UV spectra of $C_{13}H_9N$ and various isomers of $HC_{13}H_9N$ predicted with the TD-B3LYP/6-311++G(d,p) method. (a) $C_{13}H_9N$, (b) 1-$HC_{13}H_9N$, (c) 2-$HC_{13}H_9N$, (d) 3-$HC_{13}H_9N$, (e) 4-$HC_{13}H_9N$, (f) 4a-$HC_{13}H_9N$, (g) $C_{13}H_9NH$, (h) 6-$HC_{13}H_9N$, (i) 6a-$HC_{13}H_9N$, (j) 7-$HC_{13}H_9N$, (k) 8-$HC_{13}H_9N$, (l) 9-$HC_{13}H_9N$, (m) 10-$HC_{13}H_9N$, (n) 10a-$HC_{13}H_9N$, and (o) 10b-$HC_{13}H_9N$. The spectra were convoluted with a full width 30 cm^{-1} at half maximum.

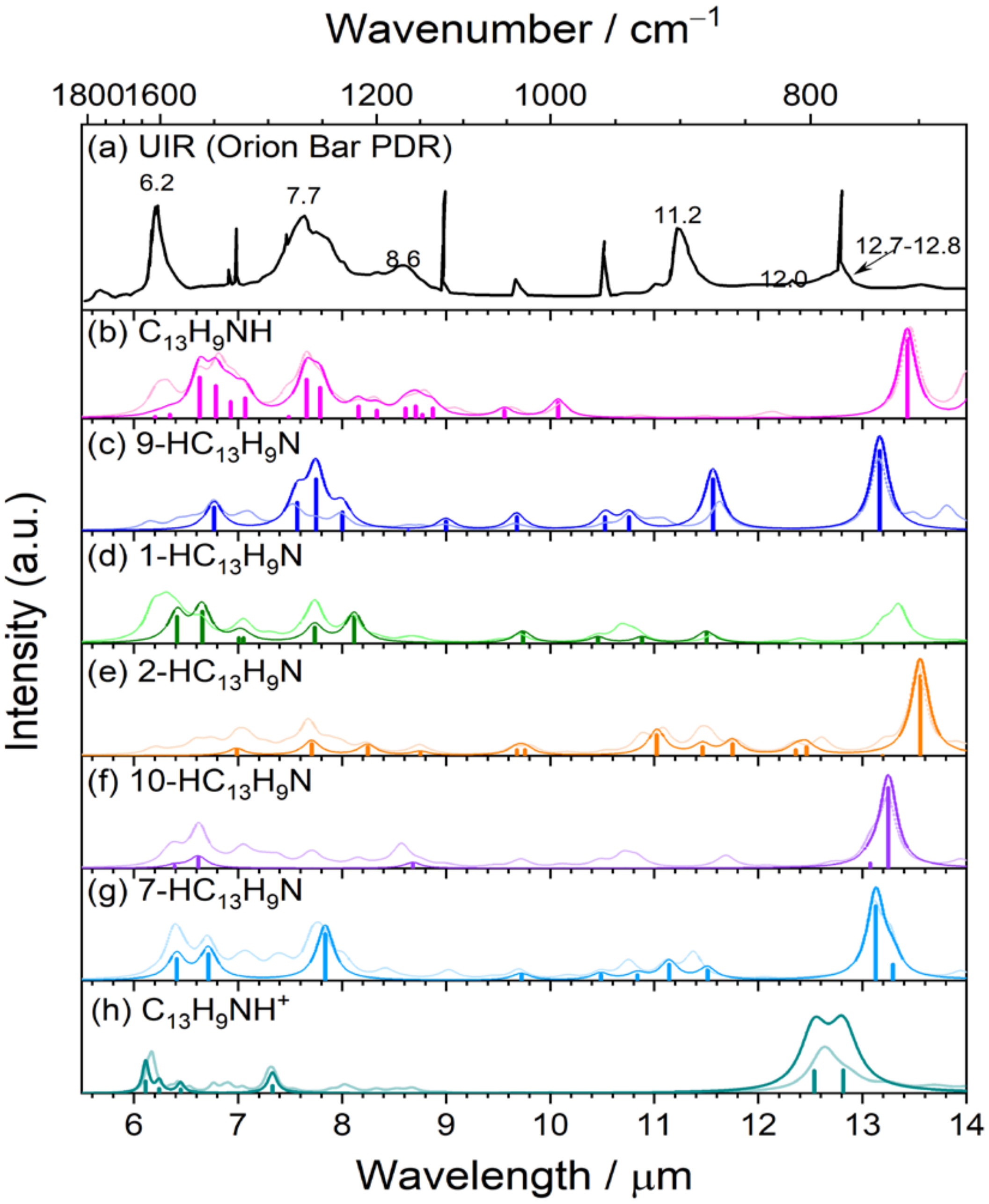

Figure S12. Comparison of UIR bands with observed spectra of protonated and hydrogenated phenanthridine. (a) The UIR emission spectrum from Orion Bar PDR. Reproduced from Peeters, E.; Mackie, C.; Candian, A.; Tielens, A. G. G. M. A Spectroscopic View on Cosmic PAH Emission. *Acc. Chem. Res.* **2021**, *54* (8), 1921–1933. Copyright [2021] American Chemical Society. Experimental spectra of (b) $C_{13}H_9NH$, (c) 9-$HC_{13}H_9N$, (d) 1-$HC_{13}H_9N$, (e) 2-$HC_{13}H_9N$, (f) 10-$HC_{13}H_9N$, (g) 7-$HC_{13}H_9N$, and (h) $C_{13}H_9NH^+$ are presented with sticks of which the heights represent integrated intensities. Dark lines represent experimental spectra convoluted with FWHM 10 cm^{-1}. Light lines represent the spectra simulated according to scaled harmonic vibrational wavenumbers and harmonic IR intensities predicted with the B3LYP/6-311++G(d,p) method, convoluted with FWHM 10 cm^{-1}.
